# *E*‐selective Semi‐hydrogenation of Alkynes under Mild Conditions by a Diruthenium Hydride Complex

**DOI:** 10.1002/chem.202202527

**Published:** 2022-10-17

**Authors:** Cody B. van Beek, Lars Killian, Martin Lutz, Markus Weingarth, Arun S. Asundi, Ritimukta Sarangi, Robertus J. M. Klein Gebbink, Daniël L. J. Broere

**Affiliations:** ^1^ Organic Chemistry and Catalysis Debye Institute for Nanomaterials Science Faculty of Science Utrecht University Universiteitsweg 99 3584 CG Utrecht (The Netherlands; ^2^ Structural Biochemistry Bijvoet Centre for Biomolecular Research Faculty of Science Utrecht University Universiteitsweg 99 3584 CG Utrecht (The Netherlands; ^3^ NMR Spectroscopy Bijvoet Centre for Biomolecular Research Department of Chemistry, Faculty of Science Utrecht University Padualaan 8 3584 CH Utrecht (The Netherlands; ^4^ Stanford Synchrotron Radiation Lightsource SLAC National Accelerator Laboratory Stanford University 94025 Menlo Park California USA

**Keywords:** alkyne semi-hydrogenation, bimetallic compounds, metal-ligand cooperativity, expanded pincer ligands, polyhydride complex

## Abstract

The synthesis, characterization and catalytic activity of a new class of diruthenium hydrido carbonyl complexes bound to the ^***t*** **Bu**^ **PNNP** expanded pincer ligand is described. Reacting ^***t*** **Bu**^ **PNNP** with two equiv of RuHCl(PPh_3_)_3_(CO) at 140 °C produces an insoluble air‐stable complex, which was structurally characterized as [Ru_2_(^*t*Bu^PNNP)H(μ‐H)Cl(μ‐Cl)(CO)_2_] (**1**) using solid‐state NMR, IR and X‐ray absorption spectroscopies and follow‐up reactivity. A reaction with KO*t*Bu results in deprotonation of a methylene linker to produce [Ru_2_(^*t*Bu^PNNP^*^)H(μ‐H)(μ‐O*t*Bu)(CO)_2_] (**3**) featuring a partially dearomatized naphthyridine core. This enables metal‐ligand cooperative activation of H_2_ analogous to the mononuclear analogue, [Ru(^*t*Bu^PNP*)H(CO)]. In contrast to the mononuclear system, the bimetallic analogue **3** catalyzes the *E*‐selective semi‐hydrogenation of alkynes at ambient temperature and atmospheric H_2_ pressure with good functional group tolerance. Monitoring the semi‐hydrogenation of diphenylacetylene by ^1^H NMR spectroscopy shows the intermediacy of *Z*‐stilbene, which is subsequently isomerized to the *E*‐isomer. Initial findings into the mode of action of this system are provided, including the spectroscopic characterization of a polyhydride intermediate and the isolation of a deactivated species with a partially hydrogenated naphthyridine backbone.

## Graphical abstract

**Two do the job**: Diruthenium hydrido carbonyl complexes bound to a PNNP expanded pincer ligand are described. Deprotonation of the methylene linkers enables cooperative activation of H_2_ allowing for the *E*‐selective semi‐hydrogenation of internal alkynes under mild conditions. Initial insights into the mechanism and the isolation of a deactivated species with an unusual structure are provided.

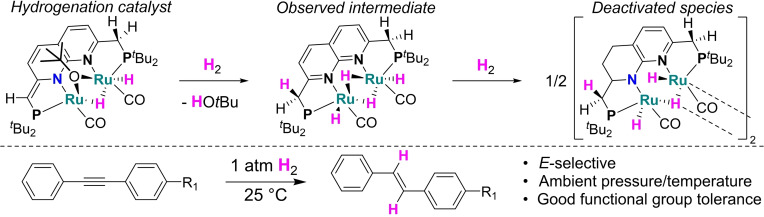

## Introduction

The selective semi‐hydrogenation of alkynes to alkenes is an important reaction in organic synthesis.^[1]^ Selective conversion of alkynes to *Z*‐alkenes is well established using either the heterogeneous Lindlar catalyst (poisoned Pd‐based)^[2]^ or homogeneous catalyst systems such as the Wilkinson or Schrock/Osborn catalysts.[^3^, ^4^] In contrast, *E*‐alkenes are commonly prepared from alkynes by a Na/NH_3_ based stoichiometric reduction, which suffers from poor functional group tolerance and produces stoichiometric amounts of waste.^[5]^ As such, the atom‐economical *E*‐selective semi‐hydrogenation of alkynes using H_2_ is still considered as a challenging reaction for the preparation of *E*‐alkenes.^[6]^ In the last decade several homogeneous catalysts have been reported for the *E‐*selective semi‐hydrogenation of alkynes[^7^, ^8^] based on platinum group metal systems (Pd,^[9]^ Ir,^[10]^ Ru,[^7^, ^11^] heterobimetallic Ag‐Ru[^8e^, ^8f^] and Ru‐Ir^[12]^) as well as first row transition metal systems (Mn,^[13]^ Fe,^[8a]^ Co,^[8b]^ Ni[^8d^, ^14^, ^15^]). However, there are few systems[^7a^, ^14^, ^15^] that enable the *E*‐selective semi‐hydrogenation at ambient temperatures and pressures of compounds that contain other functional groups that are susceptible to hydrogenation.

Inspired by natural metalloenzymes, chemists have developed various platforms that mediate making and breaking of chemical bonds through the cooperative interplay between a metal and a bound ancillary ligand.^[16]^ Such metal‐ligand cooperativity (MLC) has been exploited to catalyze various chemical transformations under milder conditions or with increased selectivity.

**Scheme 1:**
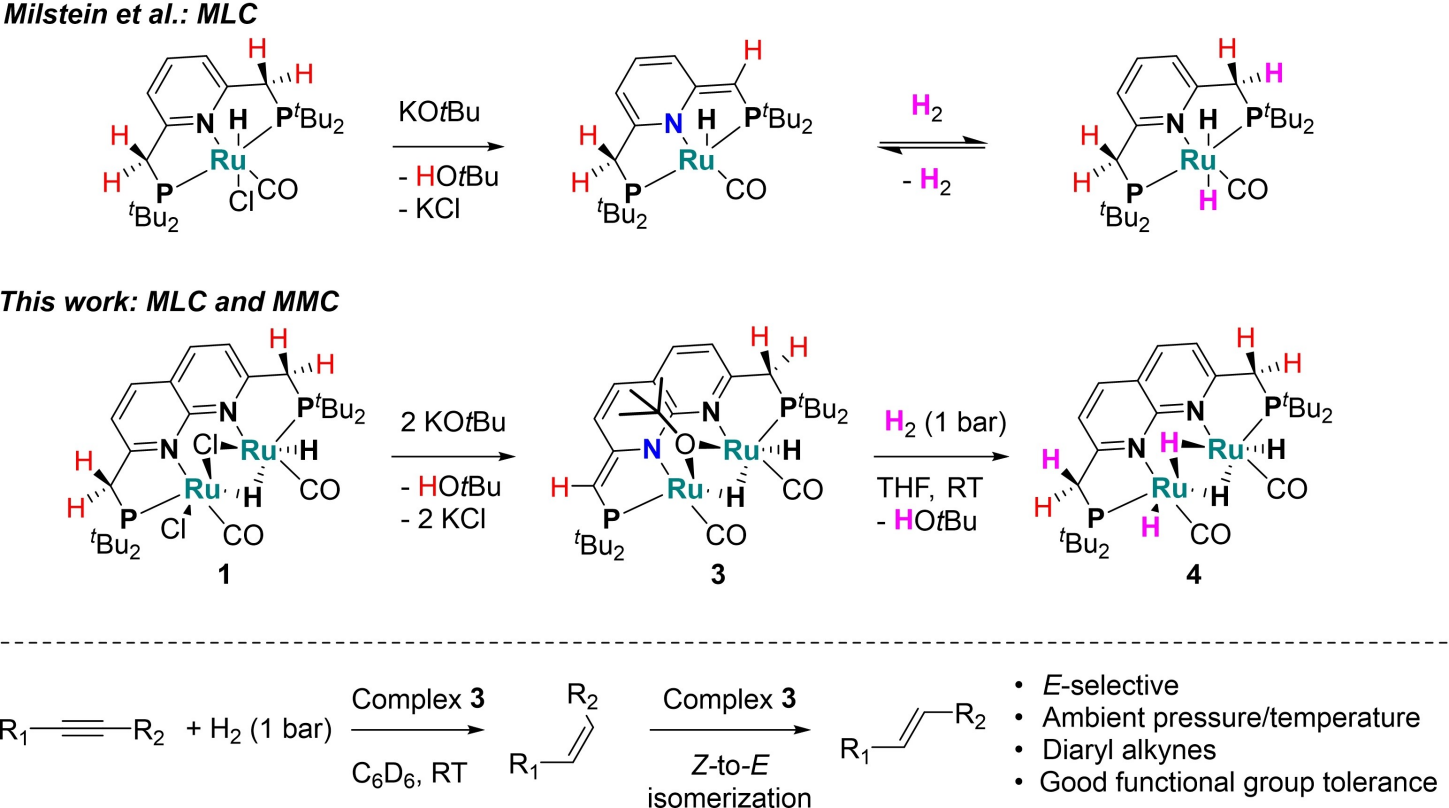
Comparison between Milstein's PNP Ru system and the PNNP diruthenium system for cooperative H_2_ activation and application for *E*‐selective alkyne semi‐hydrogenation.

A prominent platform in this field is Milstein's ruthenium hydrido carbonyl complex containing a pyridine‐based PNX (X=P, N) pincer ligand, which enables cooperative bond activation through reversible dearomatization of the ligand backbone (Scheme 1, top). These Ru based systems have found wide application in a variety of (de)hydrogenative transformations of polar bonds,^[17]^ but have not been reported for the selective semi‐hydrogenation of alkynes to the best of our knowledge.^[18]^

Another avenue in the field of cooperative bond activation and catalysis that is gaining increased attention involves well‐defined systems that utilize the cooperative action of multiple metal atoms in close proximity.^[19]^ In such systems metal‐metal cooperativity (MMC) enables distinct reactivity or selectivity from mononuclear analogues.^[20]^ Dinucleating ligands containing a 1,8‐naphthyridine core are well suited for the preparation of bimetallic complexes, as shown by the wide range of ligand scaffolds that have recently been disclosed.^[21]^ In our group, we have developed PNNP ‘expanded pincer’ ligands that can bind two metal atoms in close proximity to allow for MMC.^[22]^ Moreover, we have demonstrated that reversible dearomatization of the naphthyridine backbone allows for heterolytic bond activation through MLC. Inspired by these findings and the pivotal role of Milstein's pincer bound ruthenium hydrido carbonyl complexes in hydrogenation catalysis, we set out to synthesize the related bimetallic complex and investigate its reactivity in hydrogenative transformations.

Herein, we describe the synthesis and characterization of several diruthenium(II) hydrido carbonyl complexes supported by the ^***t*** **Bu**^ **PNNP** expanded pincer ligand (Scheme 1). Deprotonation of the methylene linkers in these complexes enables metal‐ligand cooperative activation of dihydrogen to give an active catalyst for the *E*‐selective semi‐hydrogenation of internal alkynes. We show how the diruthenium catalyst enables this transformation with good functional group tolerance under exceptionally mild conditions. Finally, we provide initial insights into the operating mechanism of this transformation, including the spectroscopic observation of a key intermediate and the isolation of a deactivated species with an unusual structure.

## Synthesis and characterization

**Scheme 2:**
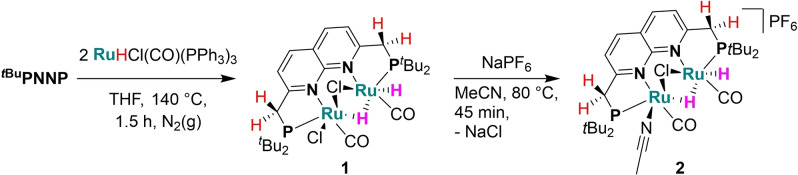
The synthesis of complex **1** from ^***t*** **Bu**^ **PNNP** and RuHCl(PPh_3_)_3_(CO) and its conversion into complex **2** by a reaction with NaPF_6_.

Reacting ^***t*** **Bu**^ **PNNP** with two equiv of RuHCl(PPh_3_)_3_(CO) in THF at 140 °C results in the precipitation of [Ru_2_(^*t*Bu^PNNP)H(μ‐H)Cl(μ‐Cl)(CO)_2_] (**1**) as an air‐stable orange powder in 75 % yield. Its insolubility in organic solvents prohibited the formation of good‐quality crystals and characterization by solution NMR spectroscopy. However, based on extensive analysis and follow‐up reactivity of complex **1** (see below) we propose a structure as depicted in Scheme 2.

**Figure 1:**
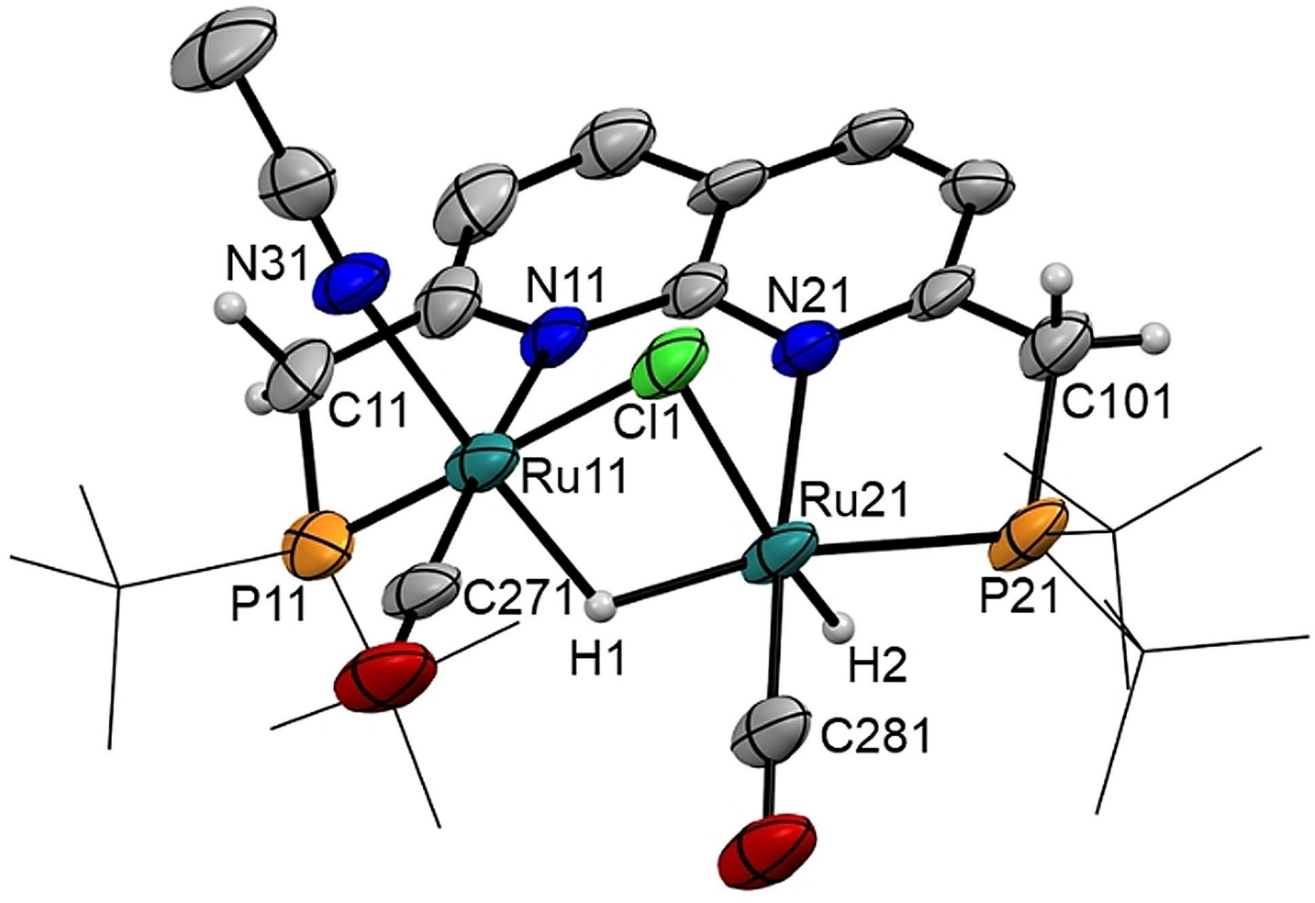
Displacement ellipsoid plot (50 % probability) of the asymmetric unit of cationic complex **2** with the ‐*t*Bu groups on P depicted as wireframe for clarity. The PF_6_^−^ anion, benzene molecules, and most hydrogen atoms are omitted. The hydride ligands are introduced at calculated positions. Ru11−Ru21 2.8149(10) Å, Ru11−P11 2.277(4) Å, Ru11−N11 2.169(6) Å, Ru11−Cl1 2.482(3) Å, Ru11−N31 2.101(7) Å, Ru11−C271 1.844(9) Å, Ru21−P21 2.275(3) Å, Ru21−N21 2.214(6) Å, Ru21−Cl1 2.528(2) Å, Ru21−C281 1.807(9) Å.

Heating a suspension of complex **1** and NaPF_6_ in acetonitrile at 80 °C results in halide abstraction to give air‐stable [Ru_2_(^*t*Bu^PNNP)H(μ‐H)MeCN(μ‐Cl)(CO)_2_]PF_6_ (**2**) as an orange solid in 30 % yield (Scheme 2).^[23]^ The ATR‐IR spectrum of complex **2** (Fig S18) shows two strong bands at ν=1972 and 1938 cm^−1^ corresponding to two terminally bound CO ligands. Furthermore, a weak band at ν=2069 cm^−1^ shows the presence of a terminal hydride ligand.^[17c]^ The ^31^P{^1^H} NMR spectrum of **2** (Fig S9) in acetonitrile‐*d*_3_ at 298 K displays two signals at δ=109.4 and 107.1 ppm and the characteristic resonance for the PF_6_^−^ anion. The ^1^H NMR spectrum of **2** in acetonitrile‐*d*_3_ (Fig S6) at 298 K displays four doublets in the aromatic region, suggesting a lack of symmetry perpendicular to the naphthyridine plane. Additionally, two AB patterns (including additional phosphorous coupling) for the four methylene linker protons are observed, showing that the ligand environment is different above and below the naphthyridine plane. Furthermore, two resonances are found at δ=−14.52 (doublet) and −15.80 ppm (doublet of doublets) corresponding to a terminal and bridging hydride ligand, respectively, based on extensive 2D analysis (see Supporting Information). A singlet at δ=1.96 ppm that integrates for 3H is assigned to a molecule of acetonitrile, which was coordinated to ruthenium and is exchanged by acetonitrile‐*d*_3_. Single crystals of **2** suitable for X‐ray crystal structure determination were grown from a standing saturated benzene solution at ambient temperature. While being inconclusive about the hydrogen atoms, the overall molecular structure (Figure 1) in the crystal is consistent with the NMR results in solution (see Supporting Information). Two Ru centers are bound within the ^***t*** **Bu**^ **PNNP** ligand that have inequivalent coordination environments, which is in agreement with the NMR data. Ru11 has distorted octahedral geometry and shows a bound bridging chloride and a terminally bound acetonitrile and terminal carbonyl ligand, whereas Ru21 with distorted octahedral geometry has a bridging chloride ligand, a terminal carbonyl ligand and hydride ligands. The hydride ligands, one terminal and one bridging, were placed at calculated positions, resulting in an 18 valence electron (VE) count for both metal centers.^[24]^ Although the Ru−Ru distance of 2.8149(10) Å is shorter than the sum of the covalent radii of 2.92 Å,^[25]^ a Ru−Ru bond is unlikely to be present.^[26]^

The spectroscopic characterization and structural insights of complex **2** enables a more substantiated proposal for the structure of complex **1** through the comparison of the spectroscopic data. The ATR‐IR spectrum of complex **1** (Fig S4) displays two strong terminal CO bands at ν=1942 and 1905 cm^−1^, which are at similar energies as in **2**. Another similarity with complex **2** is the presence of a weak absorption at ν=2021 cm^−1^, which indicates the presence of a terminal Ru−H bond.^[17c]^ The ^1^H solid‐state NMR (ssNMR) spectrum (Fig S1) is dominated by a strong signal at δ=1.2 ppm, corresponding to the ‐*t*Bu substituents. Several aromatic signals up to 9.7 ppm and resonances between 2.8–4.6 ppm are observed. Notably, four distinct resonances in the hydride region are observed at δ=−14.3 (major), −15.1 (minor), −16.8 (major) and −18.8 (minor) ppm with an integral ratio 1 to 0.1–0.2 between the major and minor set of resonances, respectively. The 1 to 0.1–0.2 ratio of the integrals suggest that two populations are present, which can be assigned to the presence of two different isomers or by different polymorphs of complex **1**.^[27]^ The ^31^P ssNMR spectrum (Fig S2) displays two main peaks at δ=110.6 and 106.8 ppm that are not well resolved, but at comparable chemical shifts as the resonances observed in the solution ^31^P{^1^H} NMR spectrum of **2**. The ^13^C ssNMR spectrum (Figure S3) displays two major resonances in the range of δ=205–210 ppm and a minor resonance at δ=210 ppm. These features are at similar chemical shifts as the resonances assigned to the carbonyl ligands in the solution spectra of **2** at δ=206 and 201 ppm (Fig S8). The similar spectroscopic features in the NMR and IR spectra of **1** and **2** suggest that no major structural rearrangements occur upon chloride abstraction from **1**. CHN combustion analysis also is in agreement with the proposed structure of **1**.

To address the question whether different isomers are present in complex **1**, we evaluated the possibility of four isomers. These proposed isomers differ solely in the arrangement of their bridging and terminal chloride and hydride ligands and all have one terminal carbonyl ligand per ruthenium center in the plane of the naphthyridine ligand (Figure S54). The relative stability of these isomers was computationally assessed by comparison of the relative energies of their DFT optimized gas phase geometries (BP86/def2‐TZVP level) (Figure S54). This showed that the geometry of complex **1**, as depicted in Scheme 2 has the lowest energy. Alternative isomer 2, where the chlorides and hydrides are not both on the same side of the naphthyridine plane is 7.9 kcal⋅mol^−1^ higher in energy. In addition, an isomer that is not consistent with the spectroscopic data above, featuring two bridging hydrides and two terminal chlorides, was found to be 8.8 kcal⋅mol^−1^ higher in energy than complex **1** (see Fig S54 and Supporting Information).

**Figure 2:**
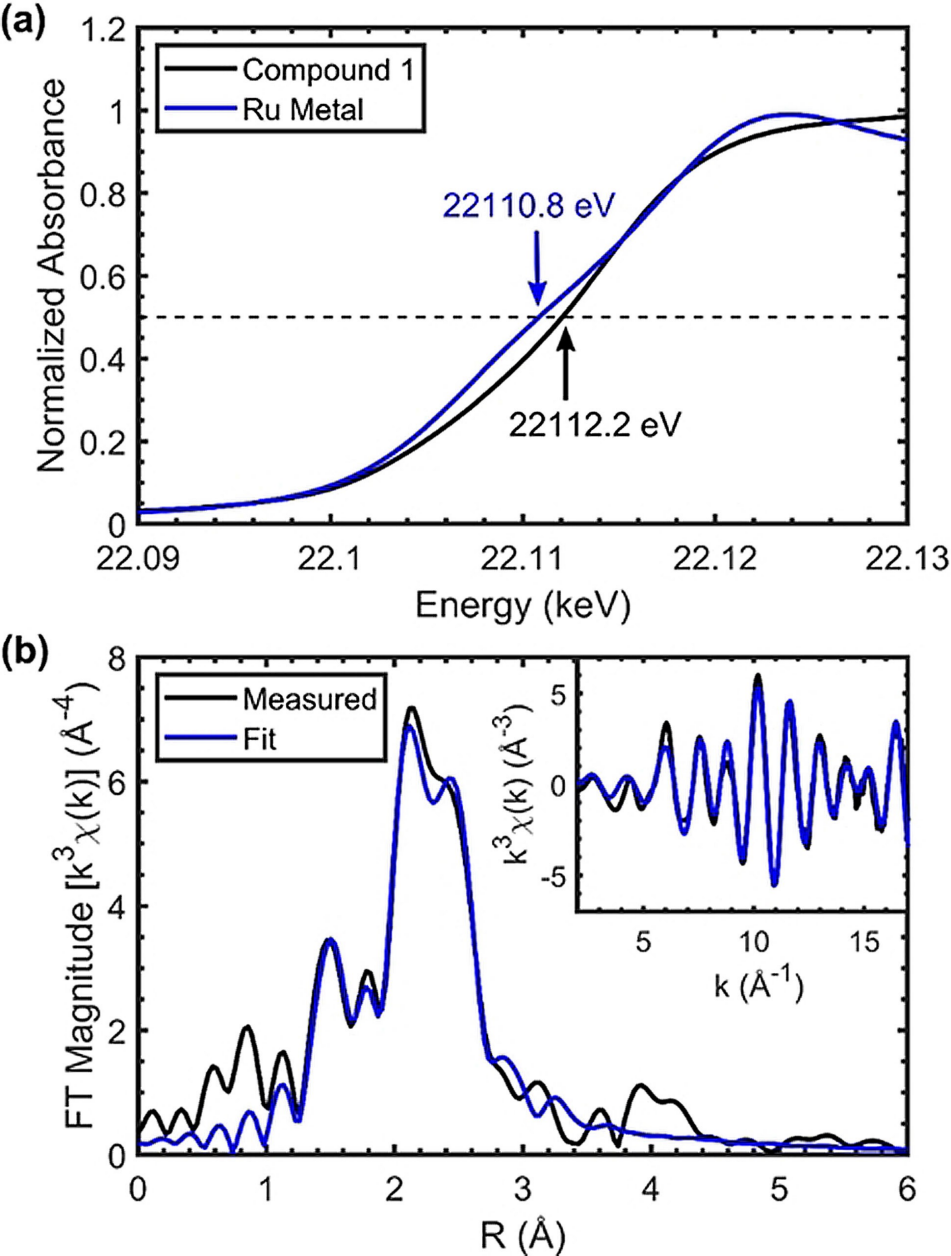
(a) Ru K‐edge XANES for **1** and a Ru metal reference. (b) Magnitude of the *k* ^3^‐weighted EXAFS Fourier Transform and model fit for **1**. Inset: EXAFS and fit in *k*‐space with *k* ^3^ weighting. Spectra were fit in a *k*‐range of 2–17 Å^‐1^ and an R‐range of 1–3 Å. Corresponding table of EXAFS fitting results is shown in Table S1.

In the absence of metrical parameters and to better assess the isomers of **1** we sought to obtain more insights through X‐ray absorption near edge structure (XANES) and extended X‐ray absorption fine structure (EXAFS) analysis. Ru K‐edge X‐ray absorption spectra were measured on a solid pellet sample of **1** and are presented in Figure 2. The rising edge energy at a normalized absorbance of 0.5 is 22112.2 eV for **1** compared to 22110.8 eV for the reference Ru metal, consistent with an increase in oxidation state of Ru in **1** (Figure 2a). The model of the EXAFS using the FEFF6 software^[28]^ (Figure 2b, Table S1) supports the DFT‐optimized structure (BP86/def2‐TZVP, see Figure S53), with 1 Ru−C at 1.82 Å, 1 Ru−N at 2.16 Å, 1 Ru−P at 2.28 Å, and, 1.5 Ru−Cl at 2.48 Å. The EXAFS fit yielded a Ru−Ru distance of 2.81 Å. These metric parameters are similar to those found for **2** by single‐crystal X‐ray structure analysis. Two Ru−C−O paths were required to fit the data well. The necessity to include these paths indicates a linearly bound CO ligand on each Ru atom, which significantly contributes to the Ru EXAFS. The EXAFS measurement provides experimental support for the structure of **1** obtained from DFT. The DFT bond lengths of isomer 2 are very similar to those of complex **1**, and thus the potential presence of isomer 2, as indicated by NMR, cannot be distinguished from a pure sample of complex **1** by this EXAFS measurement. In contrast, the computed Ru−Ru bond length in isomer 1 is 2.68 Å, significantly shorter than that measured by EXAFS. Together, the NMR, DFT, and EXAFS results show that the major product obtained when ^***t*** **Bu**^ **PNNP** is reacted with two equiv of RuHCl(PPh_3_)_3_(CO) has the structure of complex **1** and the minor product of isomer 2. Given that we do not observe a different isomer in the synthesis of **2** we reason that isomerization is facile upon halide abstraction or other reactivity (see below).

**Scheme 3:**
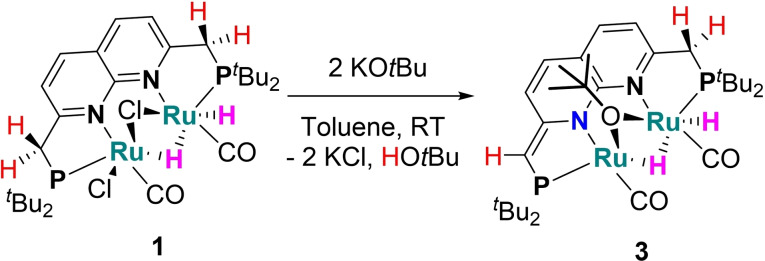
The synthesis of complex **3** by reaction of **1** with two equiv KO*t*Bu.

Given the key role of MLC through reversible dearomatization of ligand backbones in catalytic transformations with mononuclear (PNP)Ru complexes, – a feature also possible with ^***t*** **Bu**^ **PNNP** – we investigated the reactivity of complex **1** towards bases. The addition of two equiv of KO*t*Bu^[29]^ to an orange suspension of **1** in a mixture of THF/toluene/benzene results in rapid formation of a dark red suspension from which [Ru_2_(^*t*Bu^PNNP^*^)H(μ‐H)(μ‐O*t*Bu)(CO)_2_] (**3**) can be isolated as a red solid in 72 % yield (Scheme 2). In contrast to **1**, complex **3** is air‐sensitive and gradually decomposes when stored as a solid under inert atmosphere after several weeks or in a matter of days in solution based on NMR analysis. Furthermore, **3** is highly soluble in both apolar and polar solvents. The ATR‐IR spectrum of **3** (Fig S28) shows a strong terminal CO band at ν=1923 cm^−1^ and a shoulder at ν=1890 cm^−1^. In comparison to **1** and **2** these bands are at significantly lower energies showing that there is increased π‐backdonation to the CO ligands in complex **3**. The ^31^P{^1^H} NMR spectrum of **3** in THF‐*d*_8_ at 298 K (Fig S21) features two resonances at δ=104.1 and 91.4 ppm that both are magnetically coupled to the hydride ligands.^[30]^ Four aromatic resonances assigned to the naphthyridine backbone are observed at δ=6.97, 6.53, 6.46 and 6.38 ppm in the ^1^H NMR spectrum (Fig S19). The upfield shifts of these resonances and that of one of the P atoms in comparison with **2** is consistent with partial dearomatization of the ^***t*** **Bu**^ **PNNP** ligand.^[22]^ This is further supported by the observation of the three resonances at δ=4.20, 3.45 and 3.30 ppm corresponding to the methine C*H* and diastereotopic methylene C*H*_2_ hydrogens, respectively. The presence of a *tert*‐butoxide ligand is confirmed by the observation of a singlet at δ=1.13 ppm that integrates for nine protons. Furthermore, complex **3** displays two hydride signals (δ=−15.76 ppm, doublet, and −20.97 ppm, doublet of doublets) with the same coupling patterns as observed for complex **2**, implying that both a terminal and a bridging hydride ligand are present. Extensive 2D NMR analysis is also consistent with the structure of complex **3** depicted in Scheme 3 featuring a 16 VE Ru center in the dearomatized PN pocket and an 18 VE Ru center featuring a terminal hydride in the neutral PN binding pocket (see Supporting Information for detailed analysis and discussion). Despite numerous attempts, a satisfactory combustion analysis or single‐crystals of complex **3** suitable for X‐ray diffraction were not obtained.

**Figure 3:**
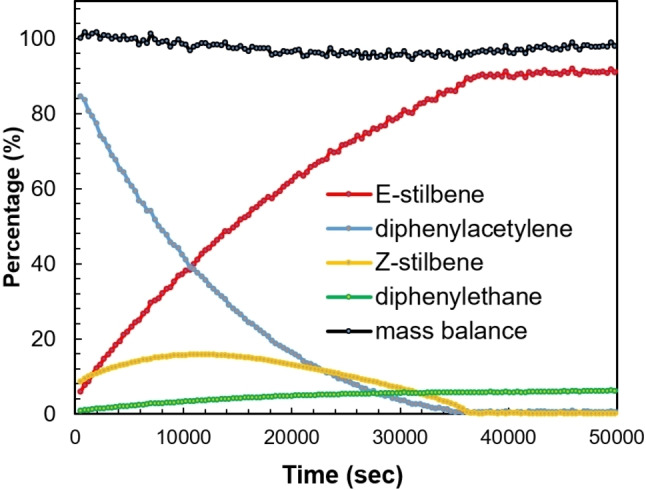
Kinetic plot of the hydrogenation of diphenylacetylene catalyzed by complex **3** showing the concentration of the various compounds based on integration of ^1^H NMR resonances vs. time.

A key feature of dearomatized 16 VE count Milstein‐type PNL−Ru systems (L=N or P) is their cooperative activation of H_2_ to form 18 VE dihydride complexes, which are involved in a variety of catalytic (de)hydrogenative chemical transformations.[^16e^, ^31^] Hence, we envisioned that the partially dearomatized ligand in complex **3** could enable similar cooperative H_2_ activation to mediate the catalytic hydrogenation of unsaturated bonds. To this end, toluene solutions of complex **3** with various unsaturated substrates were exposed to a H_2_ atmosphere (1 atm) at ambient temperature for 20 h. Under these conditions, poor conversion was observed for ketones, imines, internal alkenes and terminal alkynes. In contrast, we found that diphenylacetylene was fully consumed under these mild reaction conditions. GC‐MS and NMR analysis of the resulting mixture showed selective conversion to *E*‐stilbene, with ∼10 % of 1,2‐diphenylethane. Monitoring the semi‐hydrogenation of diphenylacetylene in C_6_D_6_ by ^1^H NMR spectroscopy using 5 mol% **3** under 1 atm of H_2_ atmosphere at 25 °C showed full conversion of diphenylacetylene after 10 h into a mixture of 94 % *E*‐stilbene and 6 % 1,2‐diphenylethane (Figure 3). The concentration of *E*‐stilbene steadily increases from the start of the experiment, but with a slower rate than the consumption of diphenylacetylene. This agrees with the observed formation of significant amounts of *Z*‐stilbene in the first hours of the reaction. The *Z*‐stilbene concentration peaks after approximately 3 h, after which its concentration decreases slowly over time. Finally, throughout the experiment a steady, yet minor, increase of the concentration of 1,2‐diphenylethane is observed. These observations show that complex **3** catalyzes both the semi‐hydrogenation of diphenylacetylene and the *Z*‐to‐*E* isomerization under mild conditions (see below). As **3** is air and moisture sensitive, we also investigated its *in situ* preparation from air‐stable **1** and KO*t*Bu. Gratifyingly, we found similar activity and selectivity using the *in situ* prepared catalyst for the semi‐hydrogenation of diphenylacetylene as with isolated **3**.

Given our findings that **3** is a poor hydrogenation catalyst for ketones, imines, internal alkenes and terminal alkynes ^[32]^ under the explored reaction conditions (see above), we set out to investigate the functional group tolerance of the *E*‐selective semi‐hydrogenation of alkynes catalyzed by **3**. Towards this end, we exposed a series of substituted alkynes to a H_2_ atmosphere (1 atm) at 25 °C in the presence of 5 mol% **3** for 24 h (Table 1). These substrates contain fragments that are prone to be reduced in metal‐catalyzed hydrogenation reactions. Note that these conditions are not optimized for a particular substrate but were chosen to enable comparison of how the various functional groups affect the conversion, selectivity, and mass balance of the reactions.

**Table 1:** Hydrogenation of diphenylacetylenes.^[a]^

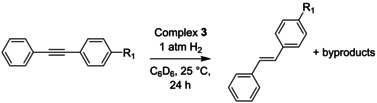							
Entry	R_1_	Conversion	*Z*‐alkene	*E*‐alkene	alkane	byproducts	Mass balance
1	H	100 %	0 %	88 %	11 %	0 %	99 %
2	COOMe	100 %	0 %	85 %	11 %	0 %	96 %
3	CN	45 %	32 %	0 %	3 %	0 %	90 %
4^[b]^	CN	100 %	0 %	91 %	6 %	0 %	97 %
5	HC=O	85 %	52 %	27 %	2 %	3 % (alcohol product)	99 %
6^[c]^	HC=O	99 %	52 %	31 %	2 %	5 % (alcohol product)	89 %
7^[b]^	HC=O	100 %	0 %	29 %	1 %	53 % *(E‐*alkene alcohol)	83 %^[d]^
8	HC=N‐Ph	100 %	0 %	65 %	6 %	11 % (amine)	82 %^[d]^
9	OMe	86 %	8 %	67 %	8 %	0 %	97 %
10^[c]^	OMe	100 %	0 %	83 %	9 %	0 %	92 %

[a] Reaction conditions: Substrate (50 μmol), complex **3** (5 mol%) were dissolved in 0.60 mL C_6_D_6_ at 25 °C under 1 atm of H_2_ for the time described. Spectroscopic yields were determined with mesitylene (3 μL) as internal standard using ^1^H NMR spectroscopy. [b] Reaction at 40 °C. [c] Reaction for 48 h. [d] The poor mass balance is due to product precipitation.

Diphenylacetylene (entry 1) was fully consumed after 24 h to the *E*‐alkene and 11 % of the alkane product under these conditions. This is in line with our findings described above, albeit with more alkane formation due to the longer reaction time. Similar conversion and selectivity is observed when an electron withdrawing methyl ester is placed on the *para* position of one of the phenyl groups (entry 2). In contrast, only 45 % conversion was observed for the substrate with a nitrile group in this position (entry 3). With this substrate we also found predominant formation of the *Z*‐alkene, similar to what was observed recently by Turculet and coworkers.^[14b]^ However, we found that performing the same reaction at 40 °C (entry 4) results in full conversion of the substrate and 91 % selectivity for the *E*‐alkene. The aldehyde bearing substrate showed slightly higher conversion under the standard conditions, but also predominantly gave the *Z*‐alkene (entry 5). Longer reaction times (entry 6) led to full conversion, but no significant change in *E*/*Z* selectivity. Performing the same reaction at 40 °C (entry 7) resulted in full isomerization to the *E*‐alkene, but also resulted in significant formation of the alkene product wherein the aldehyde was reduced to the alcohol. This product also partially precipitated resulting in a lower observed mass balance. Interestingly, the substrate substituted with an imine group (entry 8) showed full conversion under the standard conditions with decent selectivity towards the *E*‐alkene. The main side product in this reaction was the alkene product wherein the imine was hydrogenated to the amine, which also partially precipitated from the reaction mixture. The substrate containing an electron donating methoxy group (entry 9) was also found to react slower than the parent diphenylacetylene, but was fully converted after longer reaction times with comparable selectivity (entry 10). Overall, relatively minor formation of the alkane product is observed (∼5‐10 %) for all these diaryl substrates, and good functional group tolerance is observed.

Additionally, we explored the semi‐hydrogenation of 1‐phenyl‐1‐propyne and 3‐hexyne, which suffered from over‐reduction and poor *Z*‐to‐*E* isomerization similar to previous reports (see Supporting Information for details).[^13^, ^14^]

Interestingly, for most substrates the metal complex observed in solution prior to and after H_2_ addition is complex **3**, indicating that an alkyne insertion product into a Ru−H bond is not the catalyst resting state. However, for both the aldehyde, the imine and nitrile substituted substrates we observed a color change upon mixing with the catalyst that was not observed for the other substrates. Moreover, no complex **3** was observed in the ^1^H NMR spectra of these mixtures. We propose that competition between the coordination of these functional groups and the alkyne moiety to ruthenium(II), results in a slower reaction.

**Scheme 4:**
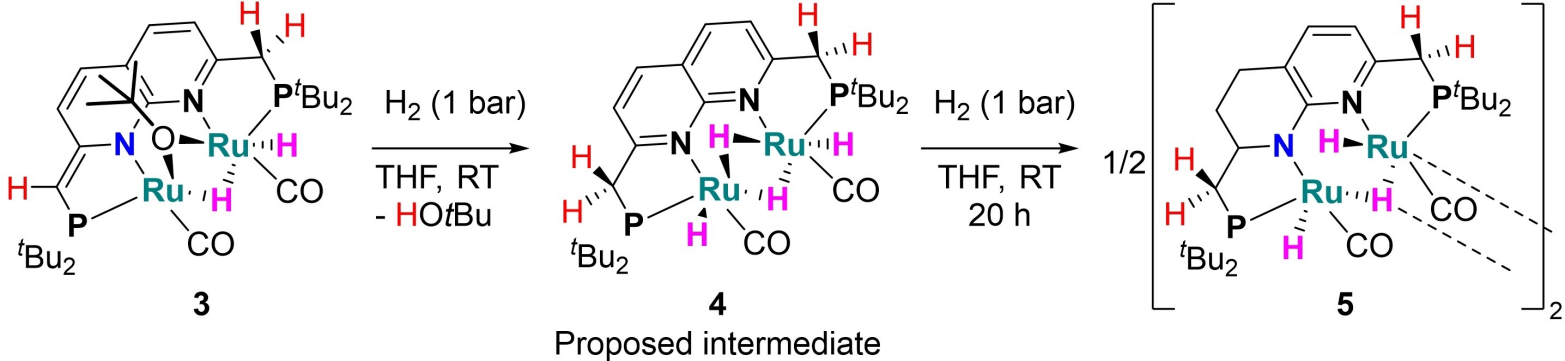
The cooperative H_2_ activation of complex **3** resulting in intermediate **4**, which subsequently is converted to complex **5**.

We observed that under the semi‐hydrogenation conditions in C_6_D_6_ with diphenylacetyene as substrate the color of the reaction mixture gradually changed from red to brown overnight. Accordingly, ^31^P{^1^H} and ^1^H NMR spectra of the resulting mixture showed the consumption of complex **3**. To gain insight into this process, more concentrated C_6_D_6_ and THF‐*h*_8_ solutions of **3** were subjected to a H_2_ atmosphere (1 atm) and analyzed by NMR spectroscopy. In the absence of substrate, a similar color change was observed in C_6_D_6_ after 24 h concomitant with the formation of an intractable mixture of species based on the ^31^P and ^1^H NMR spectra. In contrast, in THF solution the color of the solution changes from red to red‐brown within 30 minutes, concomitant with full conversion of complex **3** to a new species (**4**), which displays a single resonance at δ=100.2 ppm in the ^31^P{^1^H} NMR spectrum in THF‐*h*_8_ at 298 K (Figure S30). The ^1^H NMR spectrum (Fig S29) features two doublets at δ=8.30 (*J*_H,H_=8.2 Hz) and 7.77 ppm (*J*_H,H_=8.2 Hz) and two different resonances assigned to the methylene linker protons at δ=3.94 and 3.18 ppm, which all integrate equally. In addition, two doublets at δ=1.48 and 1.18 ppm are assigned to the ‐*t*Bu substituents. These spectroscopic features are characteristic signs of a *C*_2_‐symmetric species containing an aromatic PNNP backbone.[^22a^, ^22b^] Additionally, two hydride resonances are observed at δ=−8.85 (doublet) and −12.72 ppm (doublet of doublets), that together integrate for 4H, showing that four hydride ligands are present. These observations imply the formation of a diruthenium tetrahydride complex as depicted in Scheme 4. Its formation is proposed to involve metal‐ligand cooperative H_2_ activation and hydrogenolysis of the *tert*‐butoxide ligand, which is in agreement with the observed formation of HO*t*Bu in the ^1^H NMR spectrum. Further characterization of complex **4** was hampered due to its instability under H_2_ or N_2_ atmosphere (see Supporting Information for details).

Notably, longer reaction times (24–48 h) in either C_6_D_6_, toluene or THF gives yellow‐brown solutions concomitant with the formation of a new non‐symmetric major species [(Ru_2_(^*t*Bu^PNNP*)H_2_(μ‐H)(CO)_2_)_2_] (**5**), which was isolated as a dark brown, air‐sensitive powder in 41 % yield (Scheme 4). The ATR‐IR spectrum of **5** (Fig S52) displays three terminal carbonyl bands at ν=1950, 1925 and 1897 cm^−1^. The ^31^P{^1^H} NMR spectrum of complex **5** in DCM‐*d*_2_ (Fig S37) at 298 K displays two resonances at δ=106.7 and 105.0 ppm. In the ^1^H NMR spectrum (Fig S33), only two aromatic resonances are observed for complex **5** at δ=6.33 and 5.84 ppm together with six resonances with complex coupling patterns. In addition, three resonances are observed at δ=−13.40 (doublet of doublets), −20.25 (multiplet) and −20.46 ppm (doublet of doublets), showing that **5** contains three hydride signals of which two display mutual coupling (see Supporting Information). Together these findings indicate that the two ruthenium centers have distinct geometries.

**Figure 4:**
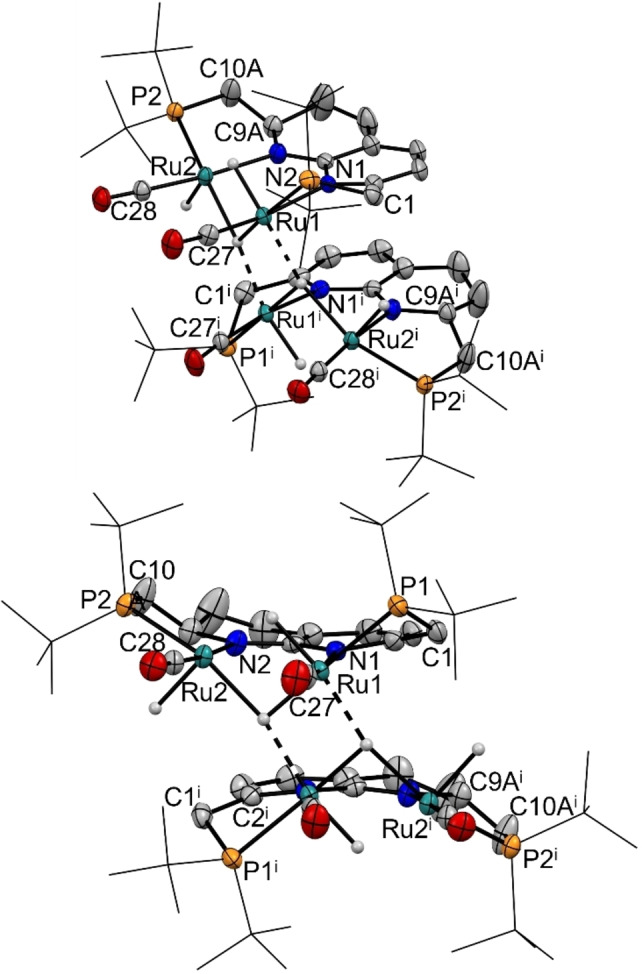
Displacement ellipsoid plots (50 % probability) of the major disorder component of the molecular structure of **5** in the crystal with the ‐*t*Bu groups on P depicted as wireframe for clarity (symmetry code i: 1‐x, 1‐y, z). Most hydrogen atoms are omitted, the hydride ligands are tentative. Ru1−Ru2 2.7364(3) Å, Ru1−Ru1^i^ 2.8627(4) Å, Ru1−P1 2.3016(8) Å, Ru1−N1 2.165(3) Å, Ru1−C27 1.828(4) Å, Ru2−P2 2.2466(8) Å, Ru2−N2 2.149(3) Å, Ru2−C28 1.833(3) Å, C1−C2 1.500(5) Å, C9A−C10A 1.414(9) Å.

Single crystals of complex **5** suitable for X‐ray diffraction were grown by layering a concentrated THF solution of **5** with hexane. The solid‐state structure (Figure 4) revealed a dimeric species which is located on a twofold rotation axis, comprised out of two (PNNP)Ru_2_(CO)_2_H_3_ fragments that are proposed to share two hydride ligands. The hydride ligands are only weakly supported by the X‐ray intensities. Furthermore, as indicated by NMR analysis, **5** contains a partially hydrogenated ligand backbone to form a 1,2,3,4‐tetrahydro‐1,8‐naphthyridine ligand backbone (see Scheme 4). The hydrogenation of *N*‐heterocyclic backbones in mononucleating pincer ligands has been observed previously, but typically requires high temperatures and H_2_ pressures.^[33]^ In contrast, the observed partial backbone hydrogenation in **5** occurs at room temperature and atmospheric pressure. We reason that facile hydrogenation is inherent to the combination of a naphthyridine‐based ligand combined with a diruthenium core given a previous report wherein similar reactivity was observed for a 2,7‐bis(2‐pyridinyl)‐1,8‐naphthyridine ligand with Ru_3_(CO)_12_.^[34]^ Complex **5** was found to be stable as a solid under an N_2_ atmosphere and no reversible H_2_ loss was observed in boiling toluene solution.

Although both Ru−Ru distances of 2.7364(3) Å (Ru1−Ru2) and 2.8627(4) Å (Ru1−Ru1^i^ between the different monomers) are shorter than the sum of the covalent radii, a formal Ru−Ru bond is not expected, just as for complex **2**. Aided by the solid‐state structure, extensive 2D NMR analysis enabled assignment of the resonances in the NMR spectra and suggests that the dimeric nature of **5** is retained in solution (see Supporting Information). This is likely due to delocalized 4c2e bonding of the hydrides to give an 18 VE configuration for all Ru centers in the dimer.

**Figure 5:**
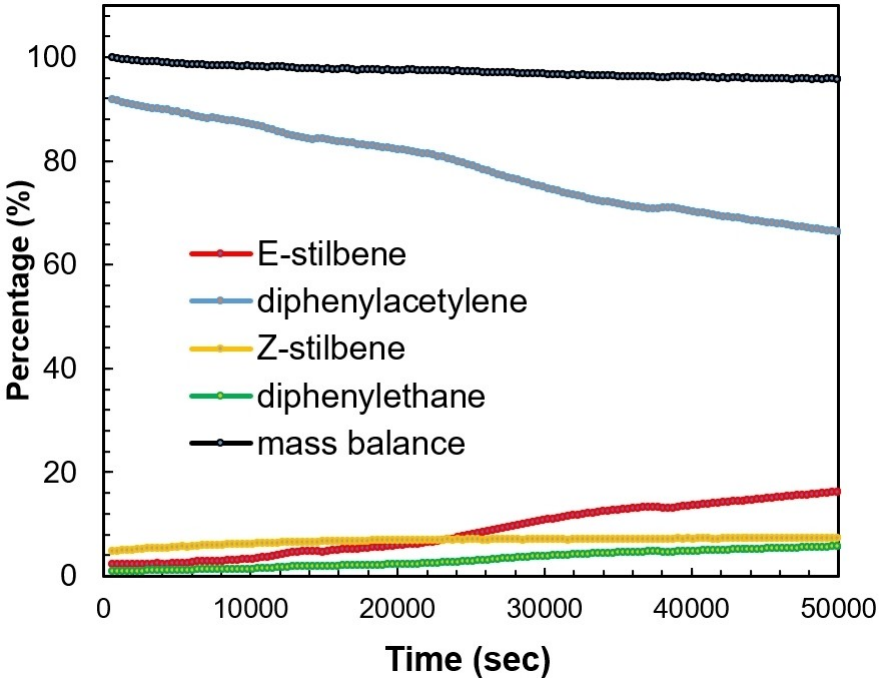
Kinetic plot of the hydrogenation of diphenylacetylene catalyzed by complex **5** showing the concentration of the various compounds based on integration of ^1^H NMR resonances vs. time.

With these insights into the fate of the complex **3** under the semi‐hydrogenation conditions, we reanalyzed the NMR spectra from the hydrogenation experiments of diphenylacetylene and found that complex **5** is progressively formed during the hydrogenation experiments. Hence, we investigated the activity and selectivity of complex **5** as a catalyst for the hydrogenation of diphenylacetylene under identical conditions (Figure 5). Interestingly, the kinetic profile of complex **5** is markedly different compared to that of complex **3**. A significantly slower conversion of diphenylacetylene is observed as after 14 h, only 31 % of the diphenylacetylene is converted and all three expected hydrogenation products, *Z*‐stilbene, *E*‐stilbene and 1,2‐diphenylethane are observed. Although the *E*‐stilbene is the major product (17 %) after 14 h, 7 % *Z*‐stilbene and 6 % of 1,2‐diphenylethane are also formed. This shows that **5** is a significantly less active and selective catalyst than complex **3** under these conditions.

For comparison we also investigated the catalytic activity and product selectivity of Milstein's monometallic 16 VE count [Ru(^*t*Bu^PNP*)H(CO)] complex^[17c]^ as a catalyst for the semi‐hydrogenation of diphenylacetylene under identical conditions (Figure S62). Notably, the rate of diphenylacetylene conversion is lower than that observed for both complexes **3** and **5** and 1,2‐diphenylethane is the major product throughout the course of the reaction, and only minor amounts of *E*‐stilbene and *Z*‐stilbene are observed.

The kinetic profile in Figure 3 suggests that initially diphenylacetylene is hydrogenated to give a mixture of both stilbenes, but that *Z*‐stilbene is isomerized to *E*‐stilbene. A control experiment wherein *Z*‐stilbene was added to a C_6_D_6_ solution containing complex **3** (5 mol%) showed full conversion to *E‐*stilbene within 10 minutes (Fig S63). This shows that the isomerization of *Z*‐stilbene already occurs with exclusion of a H_2_ atmosphere. Subsequent exposure of this mixture to a H_2_ atmosphere (1 bar), showed only minor conversion (∼5 %) to 1,2‐diphenylethane and conversion of **3** to **5** after 16 h (Fig S65). These results indicate that complex **3** is a potent catalyst for the *Z*‐to‐*E* isomerization of *Z*‐stilbene. Although complex **5** is not as active as complex **3** in the semi‐hydrogenation of diphenylacetylene, its catalytic activity for the isomerization of *Z*‐stilbene to *E*‐stilbene is comparable to that of **3** as full conversion is also observed within 10 min (using 5 mol% **5**, Fig S64). The observation that *Z*‐stilbene is present throughout the course of the semi‐hydrogenation of diphenylacetylene (Figure 3) shows that something inhibits the alkene isomerization by **3** and **5**. We found that the fast isomerization of *Z*‐stilbene to *E*‐stilbene catalyzed by **3** or **5** was unaffected by placing the reaction mixture under a H_2_ atmosphere. In contrast, when **3** or **5** (5 mol%) was added to an equimolar mixture of diphenylacetylene and *Z*‐stilbene no *Z*‐to‐*E* isomerization is observed (Fig S66‐S67). This illustrates that the alkyne substrate inhibits the isomerization of *Z*‐alkenes and explains the increased rate of consumption of *Z*‐stilbene in Figure 3 when diphenylacetylene is nearly consumed. It is clear that the *E*‐selective semi‐hydrogenation of diphenylacetylenes by **3** involves the intermediacy of the *Z*‐alkene and a subsequent isomerization step. However, the exact mechanism is still unclear and will be further investigated in our laboratories.

## Conclusion

In conclusion, the ^***t*** **Bu**^ **PNNP** ligand grants access to a new class of diruthenium(II) hydrido carbonyl complexes. These complexes show semblance to Milstein's monometallic PNP ruthenium(II) hydrido carbonyl complexes and display similar metal‐ligand cooperative activation of H_2_. However, unlike their mononuclear counterparts they are active catalysts for the *E*‐selective semi‐hydrogenation of diphenylacetylenes. The reported diruthenium system is highly active for this transformation, which can be performed at room temperature and 1 atm of H_2_ within 24 h. Moreover, a variety of functional groups that are sensitive to hydrogenation are tolerated with only aldehydes and imines showing partial conversion to the respective alcohol and amine. Preliminary mechanistic investigations suggest a mechanism that involves hydrogenation to the *Z*‐alkene followed by catalytic *Z*‐to‐*E* isomerization, which is inhibited by the alkyne substrate. Moreover, a key polyhydride species was spectroscopically observed and a deactivated species was characterized, which features a partially hydrogenated ligand backbone.

In addition to further optimization of the reaction conditions and functional group tolerance, future research in our group will focus on obtaining insights into the mechanism of the *E*‐selective semi‐hydrogenation of alkynes catalyzed by the described diruthenium complexes. We will particularly focus on the role of cooperative interplay of the two ruthenium centers in this and other (de)hydrogenative transformations.

## Experimental Section

Experimental procedures and details, NMR spectra, and computational and crystallographic details can be found in the Supporting Information. In addition, NMR and computational data files can be obtained from the 4TU database under DOI: 10.4121/20015018.

Deposition Number(s) 2160732 (for **2**) and CCDC 2160733 (for **5**) contain the supplementary crystallographic data for this paper. These data are provided free of charge by the joint Cambridge Crystallographic Data Centre and Fachinformationszentrum Karlsruhe Access Structures service.

**Procedure for the semi‐hydrogenation of internal alkynes**: 0.60 mL of a stock solution of complex **3** (2.5 μmol/0.60 mL) in C_6_D_6_ was added to the alkyne substrate (50 μmol, 20 equiv). The solutions were then transferred to J. Young valved NMR tubes and mesitylene (internal standard, 3.0 μL) was added. The mixtures were degassed by three freeze‐pump‐thaw cycles and filled with H_2_ (1 atm). The mixtures were placed in an oil bath at 25 °C (or 40 °C for some experiments) and the quantitative NMR spectra were collected after 24 h or 48 h (using an acquisition time of 5 sec and a relaxation time between scans set at 20 sec). Spectroscopic yields were determined with mesitylene as the internal standard. NMR data was recorded on an Agilent MRF 400 equipped with a OneNMR probe and Optima Tune system or a Varian VNMR−S‐400 equipped with a PFG probe at 298 K.

## Author contributions

Synthesis, characterization and catalytic experiments were performed by L. K. and C. B. B. DFT calculations were done by C. B. B. All crystallographic measurements and the corresponding data refinement was performed by M. L. ssNMR measurements were performed by M. W. X‐ray absorption experiments including data collection, fitting and interpretation were performed by A. S. A. and R. S. Project design, funding acquisition, administration and oversight were done by D. L. J. B. Supervision was done by D. L. J. B and R. J. M. K. G. The original draft was written by L. K. and C. B. B., and reviewing and editing was done by D. L. J. B. with contributions by all authors.

## Conflict of interest

The authors declare no conflict of interest.

1

## Supporting information

As a service to our authors and readers, this journal provides supporting information supplied by the authors. Such materials are peer reviewed and may be re‐organized for online delivery, but are not copy‐edited or typeset. Technical support issues arising from supporting information (other than missing files) should be addressed to the authors.

Supporting Information

## Data Availability

The data that support the findings of this study are openly available in 4TU.ResearchData at 10.4121/20015018, reference number 1.
