# Supplementary material for: E‐selective Semi‐hydrogenation of Alkynes under Mild Conditions by a Diruthenium Hydride Complex
Source: Chemistry. 2022 Oct 17;28(69):e202202527. doi: 10.1002/chem.202202527 (PMC10092327; doi:10.1002/chem.202202527)
Supplement: Supplementary file 1 — Supporting Information [file CHEM-28-0-s001.pdf]

Chemistry–A European Journal

Supporting Information

E-selective Semi-hydrogenation of Alkynes under Mild Conditions by a Diruthenium Hydride Complex

Cody B. van Beek, Lars Killian, Martin Lutz, Markus Weingarth, Arun S. Asundi, Ritimukta Sarangi, Robertus J. M. Klein Gebbink, and Daniël L. J. Broere*

Table of Contents

General Considerations 2

Syntheses and Characterization 3

Assignment of spectroscopic characterization.......... 36

X-ray absorption spectroscopy of complex 1 36

X-ray crystal structure determinations 43

Monitoring the diphenylacetylene hydrogenation by ^{1}H NMR spectroscopy 45

Isomerization catalysis 46

Semi-hydrogenation of diphenylacetylenes.......... 49

Semi-hydrogenation of alkyl substituted alkynes 60

Computational methods 65

Comparison of selected parameters for complexes 1, 2, 3 and 5 65

References 66

General Considerations

All manipulations were performed under inert atmosphere using standard Schlenk techniques or inside of a N_2-filled M. Braun glovebox using dry solvents and reagents, unless stated otherwise. Glassware was dried at 130 °C in an oven or with a heat gun under a dynamic vacuum, unless noted otherwise. Hexane, Et_2O, acetonitrile and toluene were collected from an M. Braun MB-SPS-800 solvent purification system and degassed and stored over 4 Å molecular sieves, except for acetonitrile which was dried over 3 Å molecular sieves followed by filtration over activated alumina. THF was dried over benzophenone/sodium, distilled and degassed, subsequently followed by storage over 4 Å molecular sieves. Benzene (Scharlab, >99%) and pentane (technical, VWR chemicals) were degassed, then dried and stored over 4 Å molecular sieves. All non-deuterated solvents were degassed by bubbling N_2(g) through the solvent for at least 30 min. The solvents (1.0 mL) were tested with a standard purple solution of sodium benzophenone ketyl in THF to confirm effective oxygen and water removal (max 1-2 drops for most solvents, max 4 drops for THF and Et_2O). All solvents were checked for water content by Karl-Fischer titration and should be well below 5 ppm for all solvents. Deuterated solvents were obtained from Cambridge Isotope Laboratories except for THF-d_8, which was obtained from ABCR, degassed by the standard freeze-pump-thaw procedure and stored over 4 Å molecular sieves. All commercial reagents were used as received and were obtained from Sigma Aldrich, Acros and Strem. [Ru(^{tBu}PNP)HCl(CO)],[1] methyl 4-(phenylethynyl)benzoate,[2] 4-(2-phenylethynyl)benzonitrile,[3] 4-(phenylethynyl)anisole[4] and *N*-phenyl-1-(4-(phenylethynyl)phenyl)methanimine[5] were prepared according to literature procedures. NMR data was recorded on an Agilent MRF 400 equipped with a OneNMR probe and Optima Tune system or a Varian VNMR-S-400 equipped with a PFG probe at 298 K. All chemical shifts are reported in the standard δ notation of parts per million, referenced to the residual solvent peak. All resonances in ^{1}H NMR and ^{13}C NMR spectra were referenced to residual solvent peaks (^{1}H NMR: 7.26 for $CDCl_3$, 7.16 for C_6D_6, 3.58 for THF-d_8, 5.32 for CD_2Cl_2, 1.94 for acetonitrile-d_3, ^{13}C NMR: 128.06 for C_6D_6, 67.57 for THF-d_8, 53.84 for CD_2Cl_2, 118.26 for acetonitrile-d_3). The resonances in the ^{31}P NMR spectra are referenced using the absolute reference method from a correctly referenced ^{1}H NMR spectrum of the same sample. The assignment of peaks is based on relative integration, chemical shift, and 2D NMR analysis (COSY and HMBC experiments). For ^{1}H NMR spectra in non-deuterated solvents, solvent suppression is used (Presat).

IR-data was recorded on a PerkinElmer SpectrumTwo Infrared Spectrophotometer equipped with an ATR-probe. IR-analysis of air-sensitive compounds was performed by dropcasting a THF solution onto the ATR crystal, which was covered by a continues N_2(g) flow. Elemental analysis was performed by MEDAC Ltd. based in the United Kingdom. Electrospray Ionization (ESI) measurements were performed using an Advion Expression CMS mass spectrometer. Solid state NMR measurements were performed at the NMR Spectroscopy Group (Bijvoet Center for Biomolecular Research).

Syntheses and Characterization

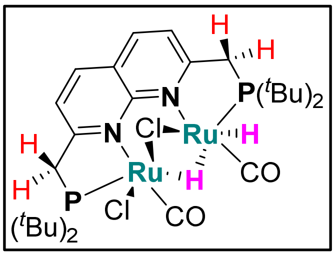

[Ru_2(^{tBu}PNNP)H(μ-H)Cl(μ-Cl)$(CO)_2$] (1) A 50 mL pressure tube was charged with **^{tBu}PNNP** (175.4 mg, 0.39 mmol) and RuHCl$(PPh_3)_3$(CO) (749.8 mg, 0.79 mmol) in THF (30 mL) and heated in an oil bath at 65 °C for 15 minutes. The resulting green solution was then heated further to 140 °C for an additional 90 minutes yielding an orange precipitate and a red supernatant. After cooling to room temperature, the orange suspension was filtered through a glass filter and washed with THF (10 mL) and DCM (20 mL). The residue was dried *in vacuo* to give complex **1** as an orange, air-stable powder (230.6 mg, 75%).

^{1}H NMR (solid state): δ = 9.70 (CH_{arom}), 8.41 (CH_{arom}), 7.23 (CH_{arom}) 4.61 (CH_2), 3.65 (CH_2), 2.85 (CH_2), 1.24 (CH_3), -14.25 (Ru*H*), -15.14 (Ru*H*), -16.84 (Ru*H*), -18.78 (Ru*H*).

^{31}P{^{1}H} NMR (solid state): δ = 110.6, 106.8 (overlapping signals).

ATR-IR (solid, under air): v = 3062 (w), 3006 (w), 2981 (w), 2961 (w), 2942 (w), 2894 (w), 2868 (w), 2021 (w), 1942 (s), 1905 (s), 1863 (w), 1698 (w), 1650 (w), 1611 (w), 1603 (w), 1543 (w), 1512 (w), 1475 (w), 1458 (w), 1435 (w), 1395 (w), 1370 (w), 1302 (w), 1275 (w), 1236 (w), 1180 (w), 1146 (w), 1020 (w), 935 (w), 856 (w), 822 (w), 810 (w), 786 (w), 659 (w), 614 (w), 601 (w), 559 (w), 537 (w), 521 (w), 483 (w), 437 (w) cm^{-1}.

Anal. Calcd. For $C_{28}H_{46}Cl_2N_2O_2P_2Ru_2$: C, 43.25; H, 5.96; N, 3.60. Found: C, 43.50; H, 5.81; N, 3.69.

Melting point: Decomposition at 350 °C.

Note: The reaction can also be performed at lower temperature in a closed system (e.g. 80 °C in a Schlenk bomb). This protocol takes longer (10 days under these conditions). The yield was 82% and IR spectroscopy (**Figure**) as well as further reactivity suggested the same product.

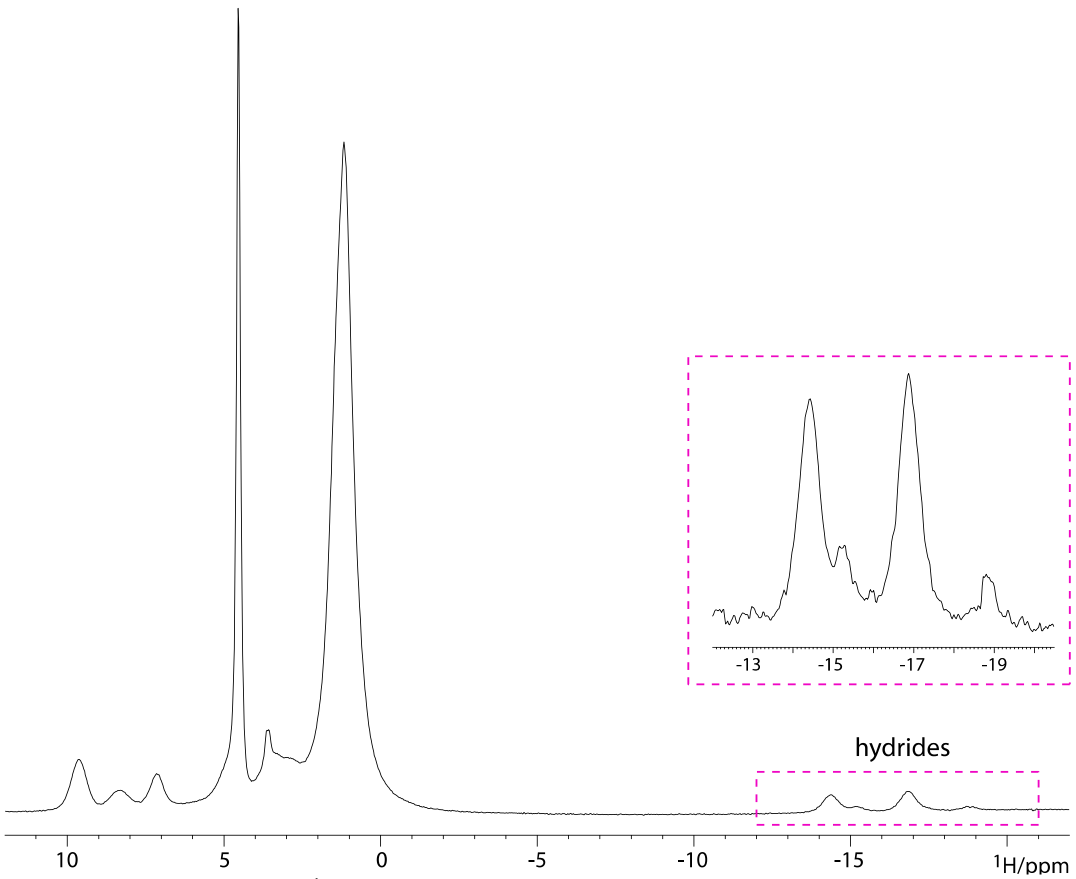

Figure S1: The solid-state ^{1}H NMR spectrum of complex **1** acquired at 800 MHz using 60 kHz magic angle spinning (MAS).

In Figure S1, two sets of signals are observed for the hydrides in complex **1**. The second set of signals has ~10-20% of the signal intensity of the major set of signals. Given that only one set of signals is observed for the remainder of complex, these data suggest that the second set of hydride signals relates to a minor difference at the metal center, e.g. different geometry and different bond lengths and bond angles. Grinding complex **1** with cellulose (like is performed during sample preparation before X-ray absorption spectroscopy) does not lead to differences of the signals corresponding to complex **1**.

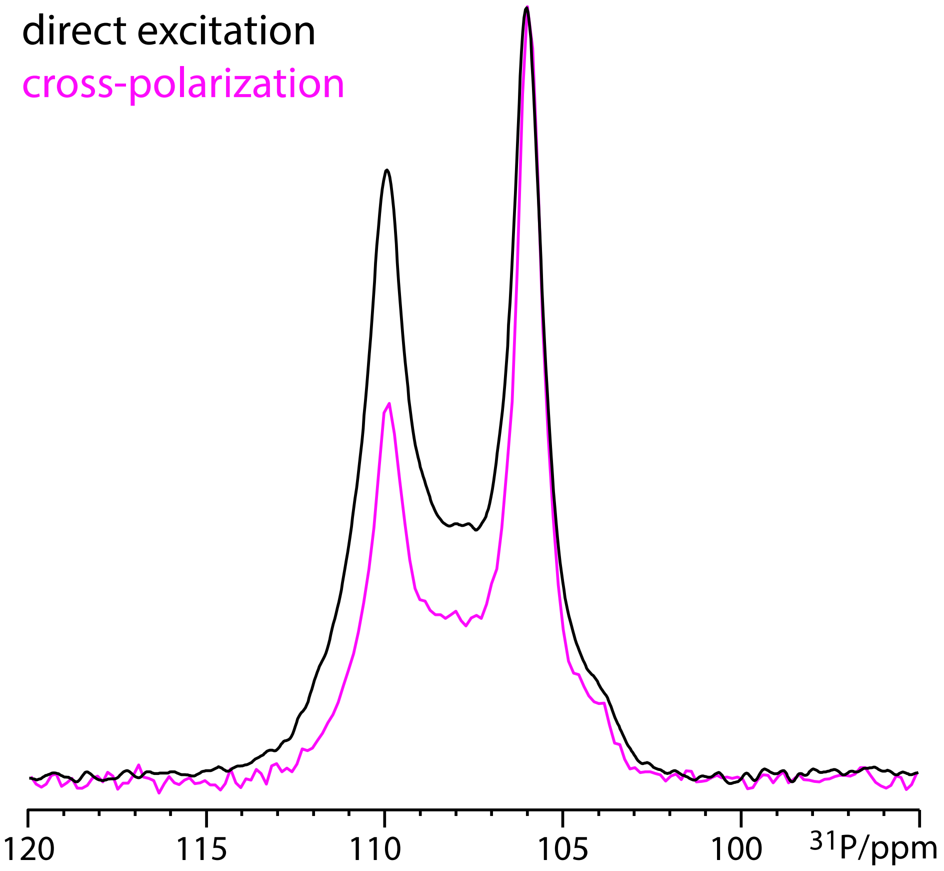

Figure S2: The solid-state $^{31}P\{^{1}H\}$ NMR spectrum of complex **1** acquired at 500 MHz (^{1}H-frequency) and 12 kHz MAS using ^{31}P excitation (in black) and ^{1}H-^{31}P cross-polarization transfer. Both spectra were acquired with 80 kHz ^{1}H-decoupling during acquisition and a very long interscan delay (120 s) to assure complete signal relaxation. The spectra are normalized.

As the intensity of the signal at 111 ppm in Figure S2 in the cross-polarization spectrum is relatively lower compared to the direct excitation spectrum, one can deduce that this ^{31}P atom is more distal to protons than the ^{31}P atom that resonates at 106 ppm. In other words, the ^{31}P atom that resonates at 106 ppm is assigned to the ^{31}P atom bonded to the ruthenium center having both a terminal and bridging hydride (see the proposed structure of complex **1**). The ^{31}P atom resonating at 111 ppm is assigned to the ^{31}P atom bonded to the ruthenium having both a terminal and bridging chloride ligand. The spectra display signal intensity between the two major signals, suggesting the presence of a second set of signals between these signals, in agreement with the ^{1}H NMR spectrum (Figure S1).

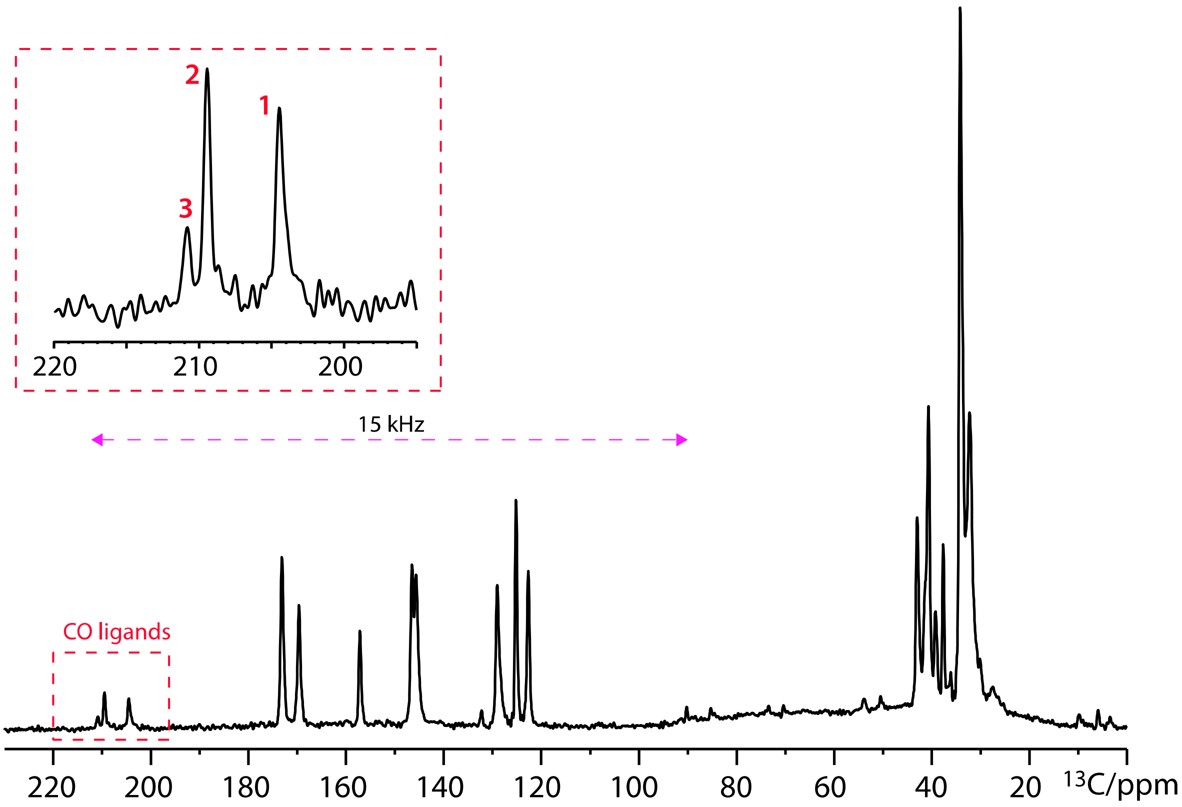

Figure S3: The solid-state ^{13}C cross-polarization NMR spectrum of complex **1** acquired at 500 MHz (^{1}H-frequency) and 15 kHz MAS, using 3 ms proton to carbon magnetization transfer time (contact time).

In Figure S3, weak but clear signals of the CO ligands appear at 210-205 ppm. Next to two major signals at 205 and 209 ppm, a third minor signal resonates at 210 ppm. While the signal intensity cannot be quantified due to the dependency on the proton environment, the population of the minor conformations observed in the ^{13}C and ^{1}H ssNMR spectra seems to match. Note that the minor CO signal is not a spinning sideband, as indicated by the magenta arrow that shows that there is no signal at 15 kHz spectral distance. Thus, all ssNMR spectra of complex **1** show the presence of a second isomer/conformation.

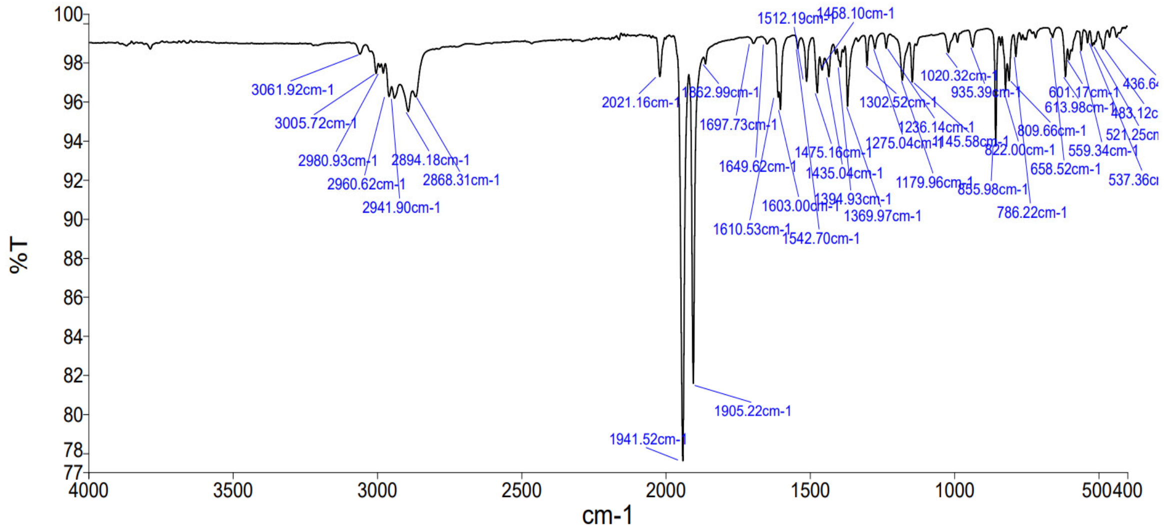

Figure S4: The ATR-IR spectrum of complex **1** measured as a solid under air.

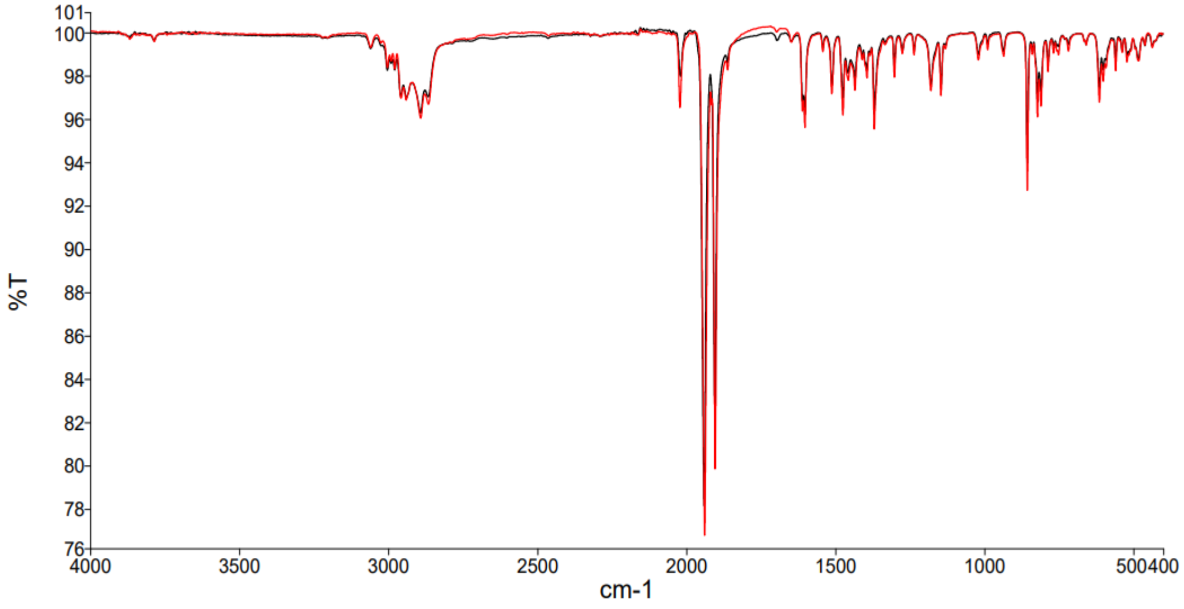

Figure S5: Overlay of the ATR-IR spectrum of complex **1** obtained from the method reported (140 °C, 90 minutes) in black and the method described in the note (80 °C, 10 days) in red.

The IR spectrum of complex **1** in Figure S4 displays a weak absorption at 1863 cm^{-1} corresponding to a carbonyl ligand vibration. This weak absorption is present in the isolated product obtained from either method described for the preparation of complex **1** (140 °C for 90 minutes or 80 °C for 10 days, see Figure S5). This weak absorption is ascribed to the carbonyl ligand vibration of the minor isomer/product present in complex **1** as described in the main text and for the ssNMR data here above.

[(^{tBu}PNNP)$Ru_2H_2Cl(CO)_2$MeCN][PF_6] (2) A Schlenk flask was charged with complex **1** (108.1 mg, 13.9 mmol) and $NaPF_6$ (30 mg, 19 mmol) in acetonitrile (3 mL). The suspension was heated in an oil bath to 45 °C. After an hour, the temperature was increased to 80 °C for 45 min yielding a green solution. From this moment, the procedure was carried out under air and without dried solvents and glassware at ambient temperature. The solution was filtered over a filter paper, and the filtrate was concentrated in vacuo. The mixture was dissolved in acetonitrile (3 mL), and diethyl ether (15 mL) was added to precipitate a dark colored solid. This solid was washed with benzene (5 mL), dissolved in acetonitrile (2 mL), and again precipitated with diethyl ether. The residue was collected yielding complex **2** as an orange, air-stable powder (38.1 mg, 30 %).

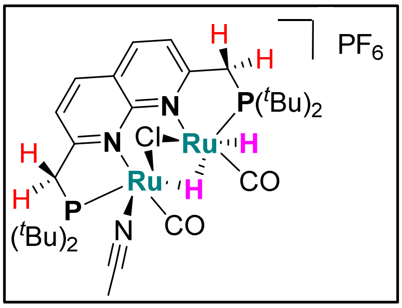

^{1}H NMR (400 MHz, acetonitrile-*d₃*, 298 K): δ = 8.50 (d, $^3J_{H,H}$ = 8.2 Hz, 1H), 8.46 (d, $^3J_{H,H}$ = 8.2 Hz, 1H), 7.85 (d, $^3J_{H,H}$ = 8.3 Hz, 1H), 7.80 (d, $^3J_{H,H}$ = 8.3 Hz, 1H), 4.27 (dd, $^2J_{H,H}$ = 18.6, $^2J_{H,P}$ = 5.9 Hz, 1H), 4.15 (dd, $^2J_{H,H}$ = 18.6, $^2J_{H,P}$ = 12.5 Hz, 1H), 3.98 (dd, $^2J_{H,H}$ = 16.5, $^2J_{H,P}$ = 10.2 Hz, 1H), 3.91 (dd, $^2J_{H,H}$ = 16.4, $^2J_{H,P}$ = 8.2 Hz, 1H), 1.99 (s, 3H), 1.56 (overlapping doublets (dd), $J_{apparent}$ = 17.6, 14.1 Hz, 18H), 1.21 (overlapping doublets (dd), $J_{apparent}$ = 22.4, 14.0 Hz, 18H), -14.52 (d, $^2J_{H,P}$ = 23.4 Hz, 1H), -15.80 (dd, $^2J_{H,P}$ = 22.2, $^2J_{H,P}$ = 14.6 Hz, 1H).

^{13}C{^{1}H} NMR (101 MHz, acetonitrile-*d₃* 298 K): δ = 206.8 (d, $^2J_{C,P}$ = 13.7 Hz), 201.4 (d, $^2J_{C,P}$ = 13.4 Hz), 170.7 (d, $^2J_{C,P}$ = 4.7 Hz), 169.9 (d, $^2J_{C,P}$ = 3.1 Hz), 155.7 (s), 142.2 (d, $^3J_{C,P}$ = 1.0 Hz), 141.1 (s), 123.8 (overlapping doublets (t), $J_{apparent}$ = 8.7 Hz), 123.2 (s), 39.28 – 38.7 (m), 38.2 – 37.3 (m), 36.3 (d, $^1J_{C,P}$ = 19.8 Hz), 30.2 (m), 28.9 (d, $^2J_{C,P}$ = 2.8 Hz), 28.7 (d, $^2J_{C,P}$ = 3.5 Hz).

^{31}P{^{1}H} NMR (162 MHz, acetonitrile-*d₃*, 298 K): δ = 109.4 (s, 1P), 107.1 (s, 1P), -144.6 (hept, $^1J_{P,F}$ = 706.4 Hz, 1P).

^{19}F NMR (376 MHz, acetonitrile-*d₃*): δ = -72.8 (d, $^1J_{F,P}$ = 706.5 Hz, 6H).

ATR-IR (solid, under air): v = 2943 (w), 2909 (w), 2873 (w), 2069 (w), 1972 (s), 1938 (s), 1608 (w), 1599 (w), 1511 (w), 1480 (w), 1463 (w), 1407 (w), 1373 (w), 1301 (w), 1183 (w), 1151 (w), 1025 (w), 936 (w), 856 (w), 835 (w), 783 (w), 615 (w), 557 (w) cm^{-1}.

Anal. Calcd. For $C_{30}H_{49}ClF_6N_3O_2P_3Ru_2$: C, 38.82; H, 5.32; N, 4.52. Found C, 38.94; H, 5.35; N, 4.35.

Note: The synthesis can also be carried out without first heating at 45 °C, as well as completely under air. By skipping the second precipitation step, a yield of 73% has been achieved with small loss of purity as observed in NMR (**Figure**). In a representative, separate experiment with a triphenylphosphine oxide internal standard, a spectroscopic yield of 87% was determined with ^{31}P NMR spectroscopy (**Figure**).

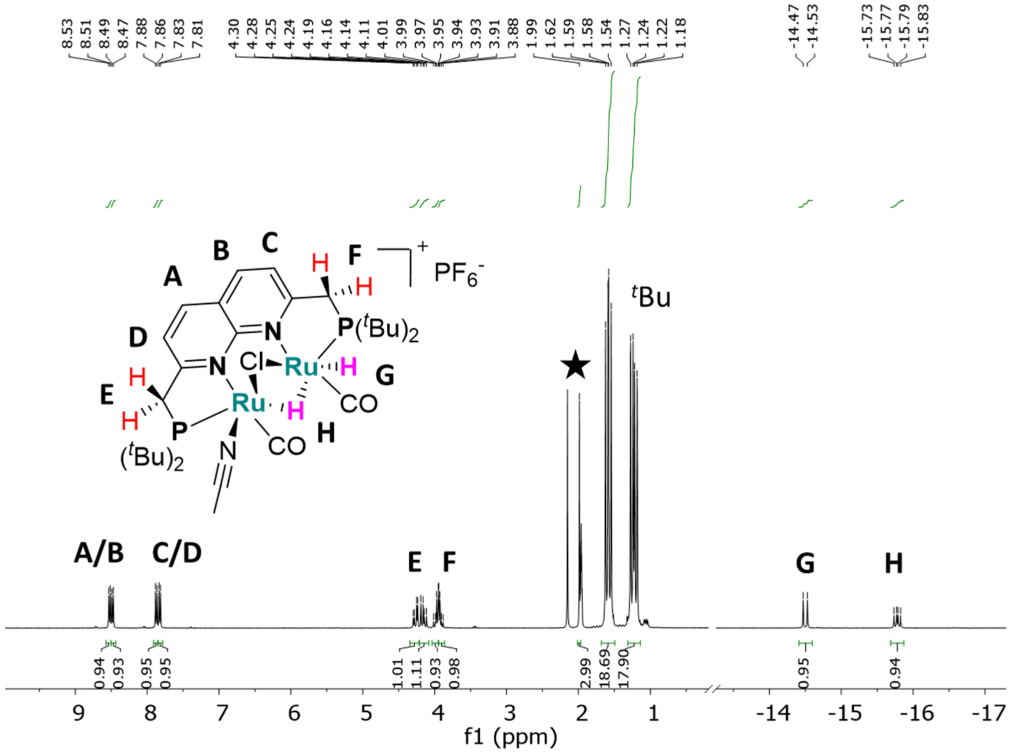

Figure S6: The ^{1}H NMR spectrum of compound **2** in acetonitrile-d_3 at 25 °C. Resonances marked with a star are assigned to acetonitrile-d_2, acetonitrile and water.

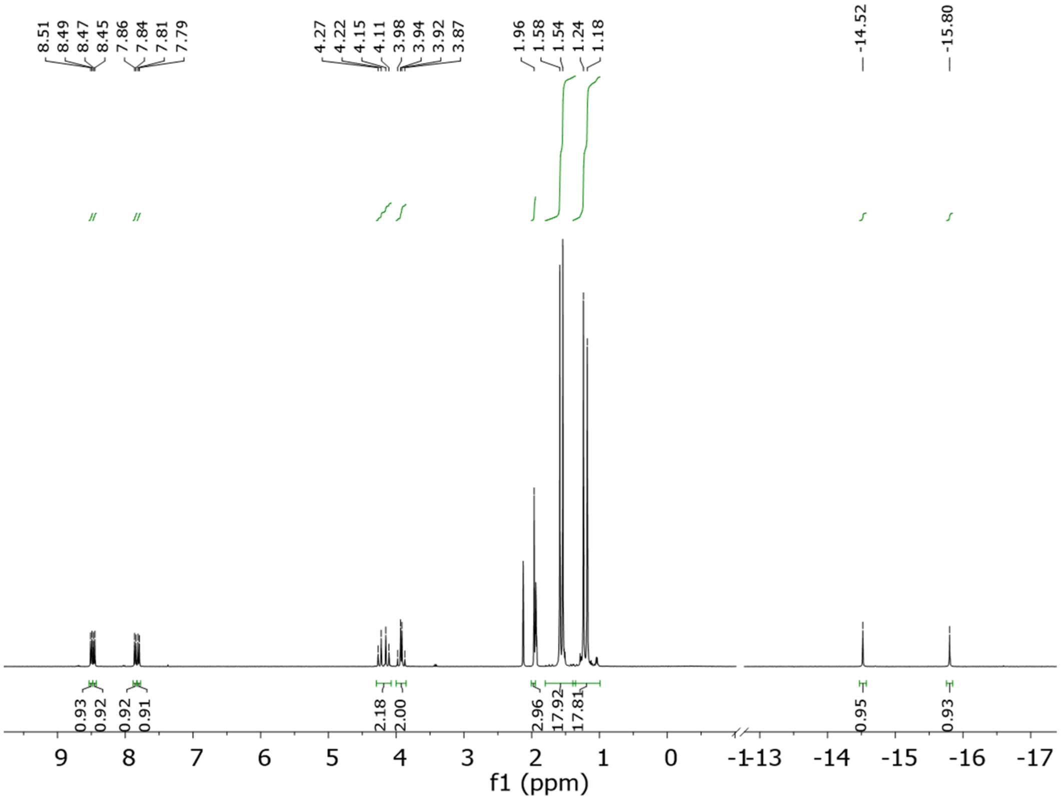

Figure S7: The ^{1}H{^{31}P} NMR spectrum of complex **2** in acetonitrile-d_3 at 25 °C.

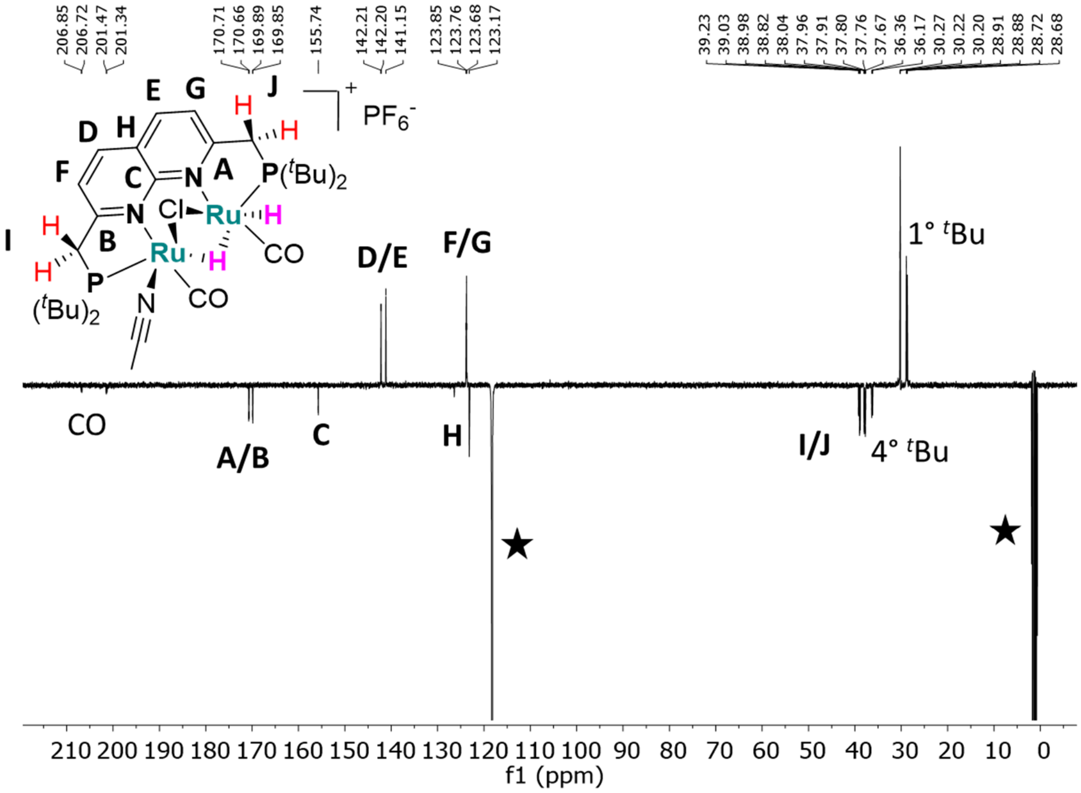

Figure S8: The ^{13}C-APT NMR spectrum of complex **2** in acetonitrile-d_3 at 25 °C. Signals with a star are assigned to acetonitrile-d_3.

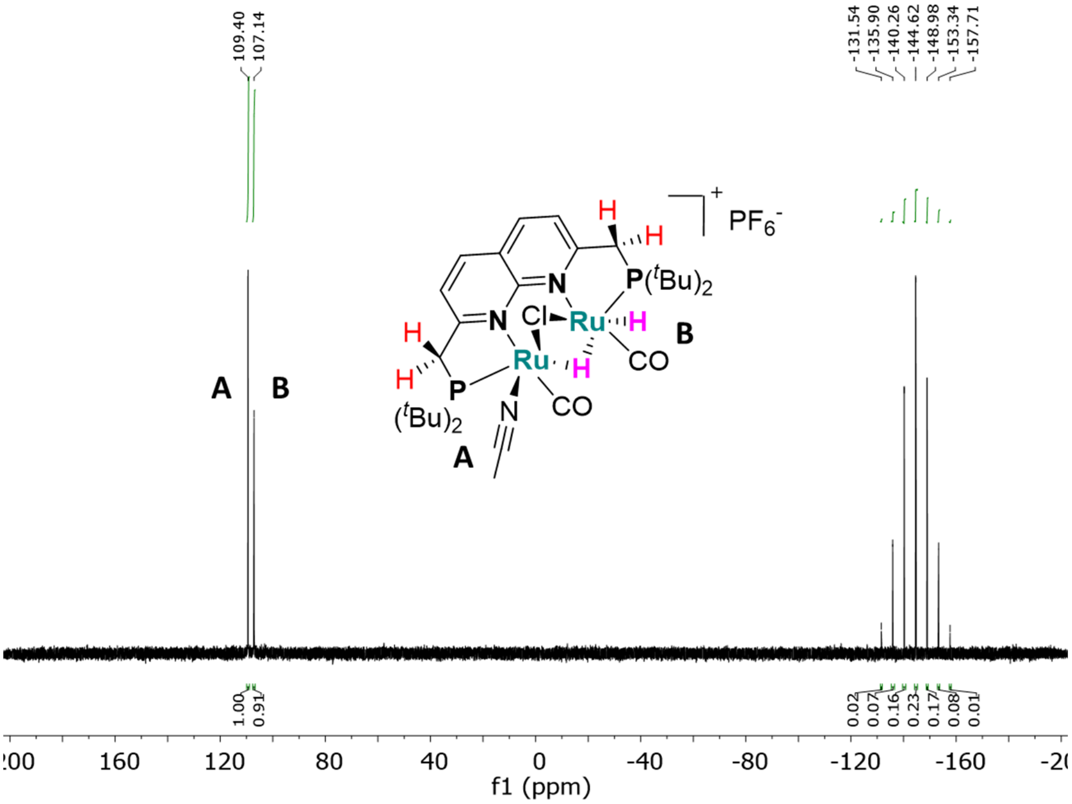

Figure S9: The ^{31}P{^{1}H} NMR spectrum of complex **2** in acetonitrile-d_3 at 25 °C.

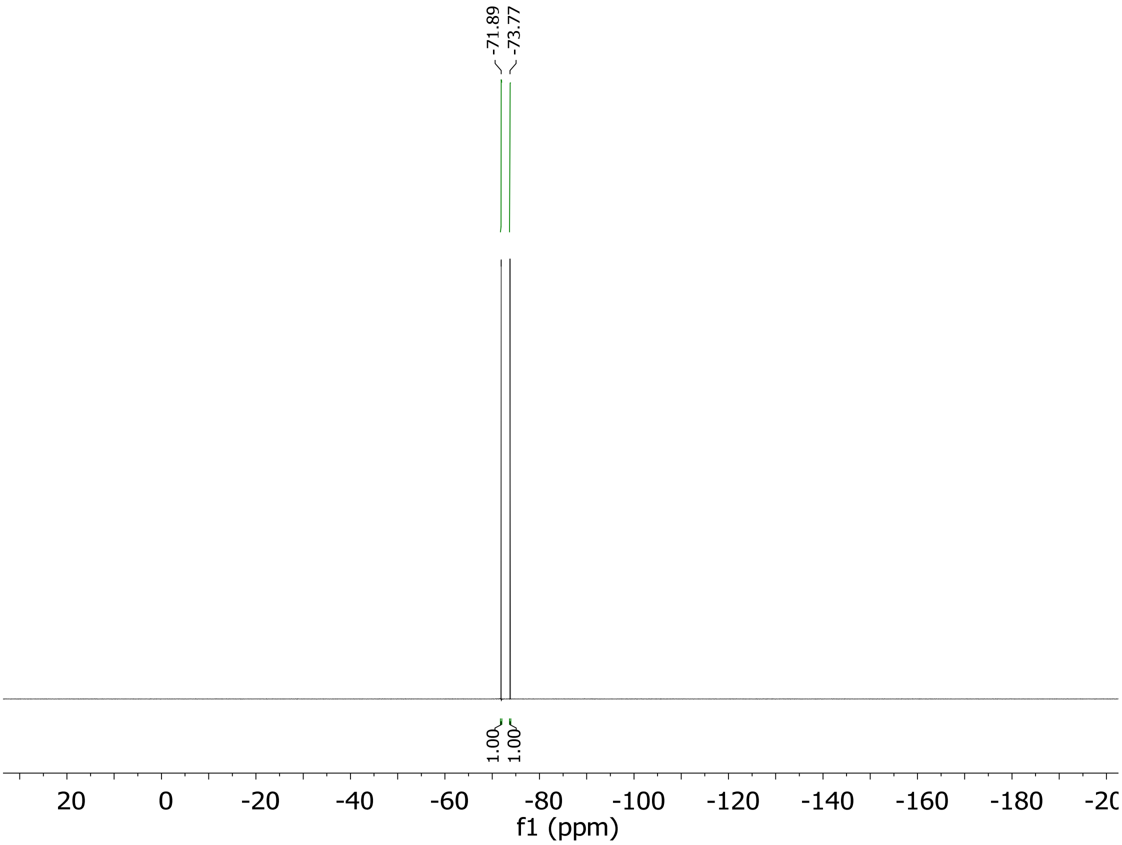

Figure S10: The ^{19}F NMR spectrum of complex **2** in acetonitrile-d_3 at 25 °C.

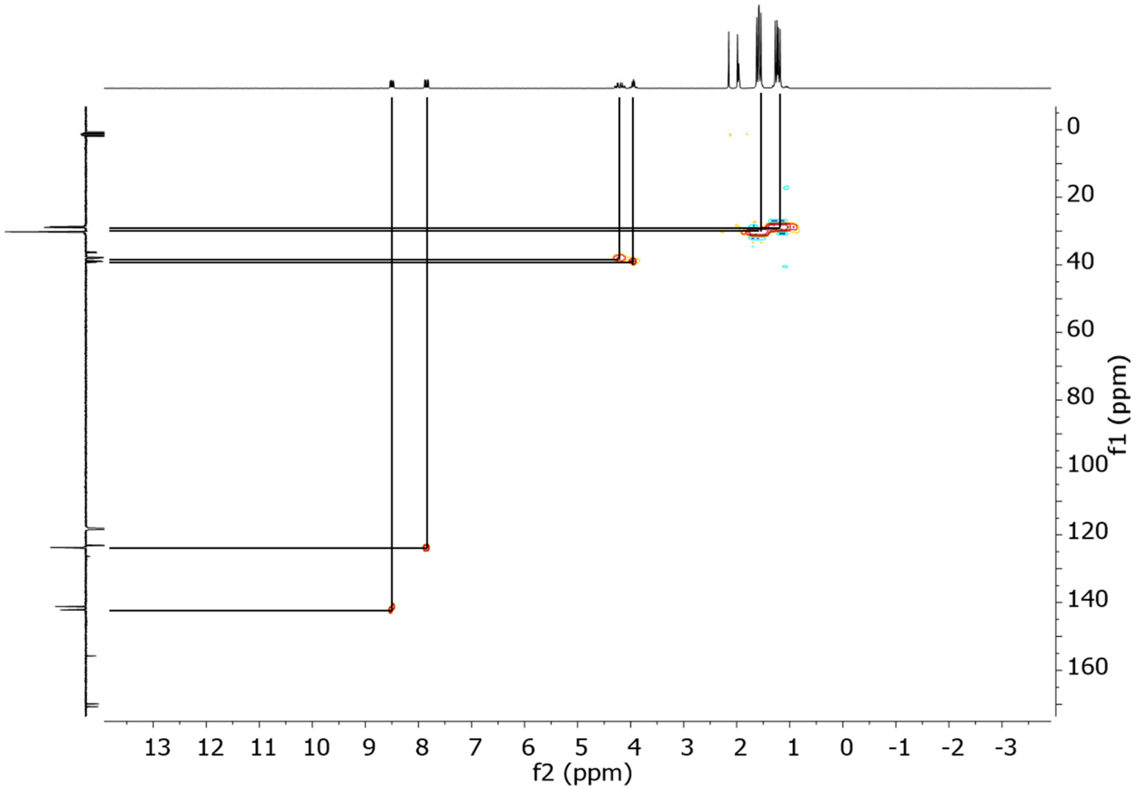

Figure S11: The ^{1}H-^{13}C HMQC NMR spectrum of complex **2** in acetonitrile-d_3 at 25 °C.

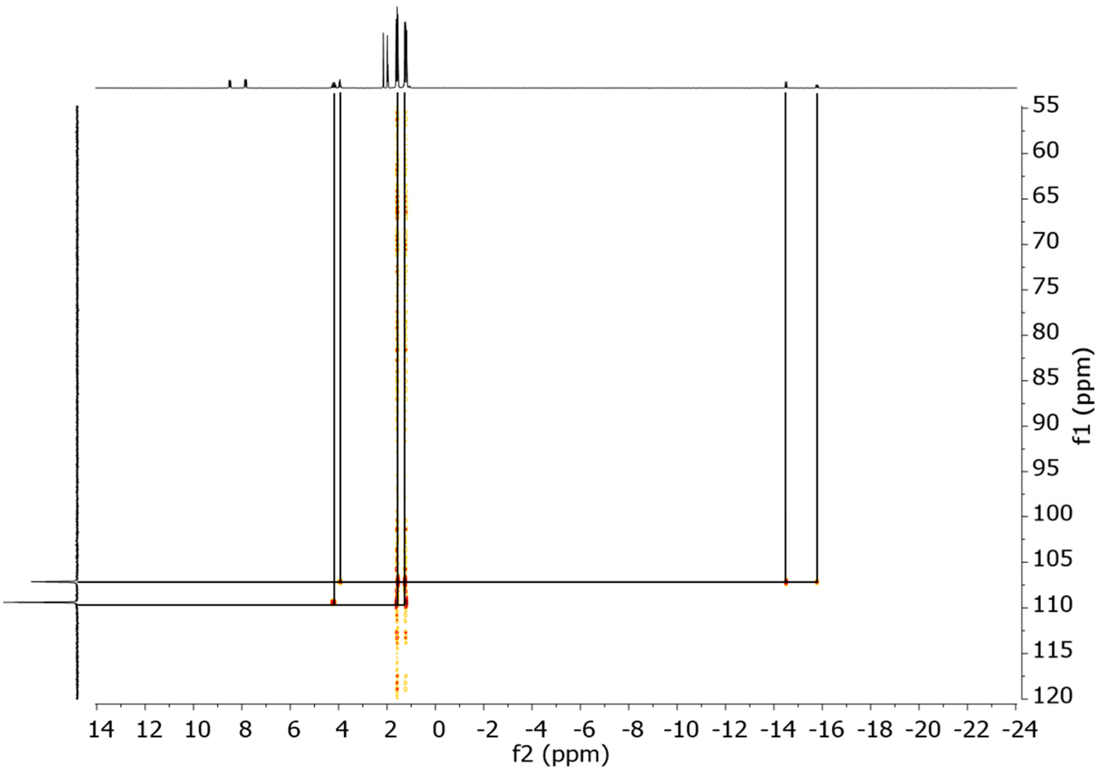

Figure S12: The ^{1}H-^{31}P HMBC NMR spectrum (multiple bond $J_{H,P}$-coupling = 8 Hz) of complex **2** in acetonitrile-d_3 at 25 °C.

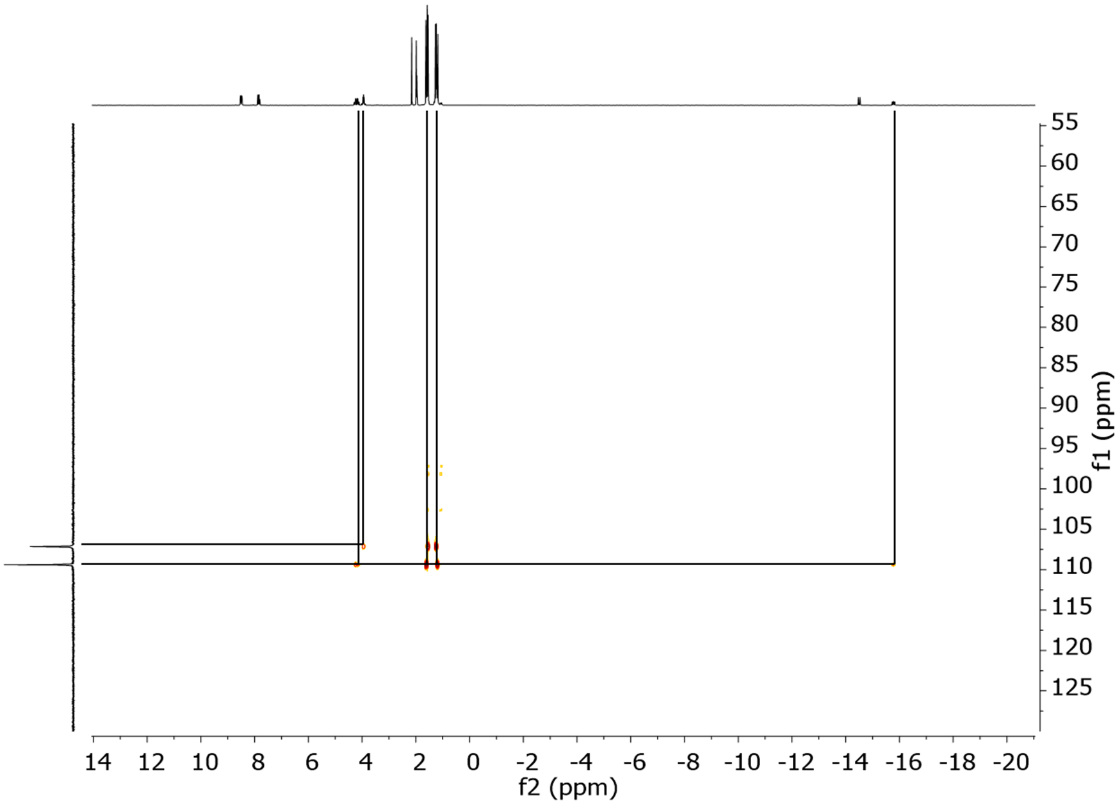

Figure S13: The ^{1}H-^{31}P HMBC NMR spectrum (multiple bond $J_{H,P}$-coupling =12 Hz) of complex **2** in acetonitrile-d_3 at 25 °C.

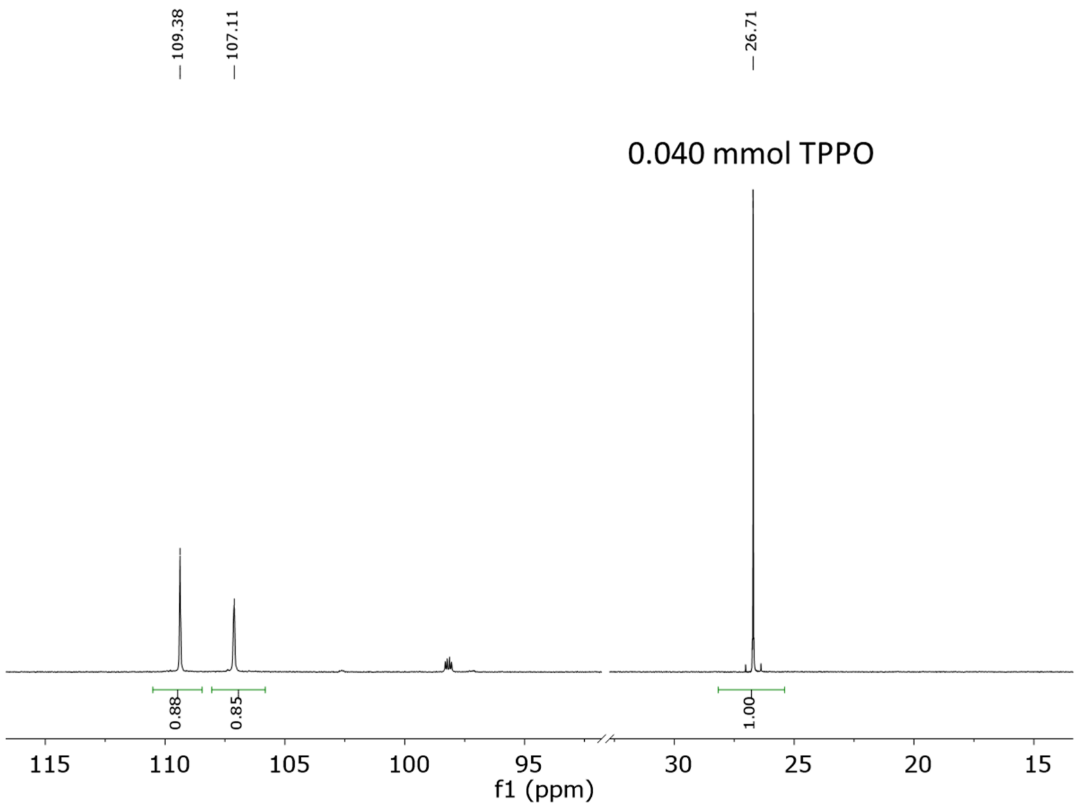

Figure S14: The $^{31}P\{^{1}H\}$ NMR spectrum in acetonitrile-d_3 at 25 °C of the crude reaction mixture of the reaction (45 minutes, 80 °C, N_2 atmosphere) of 0.040 mmol complex **1** with 0.056 mmol $NaPF_6$. A relaxation time of 5 seconds was used.

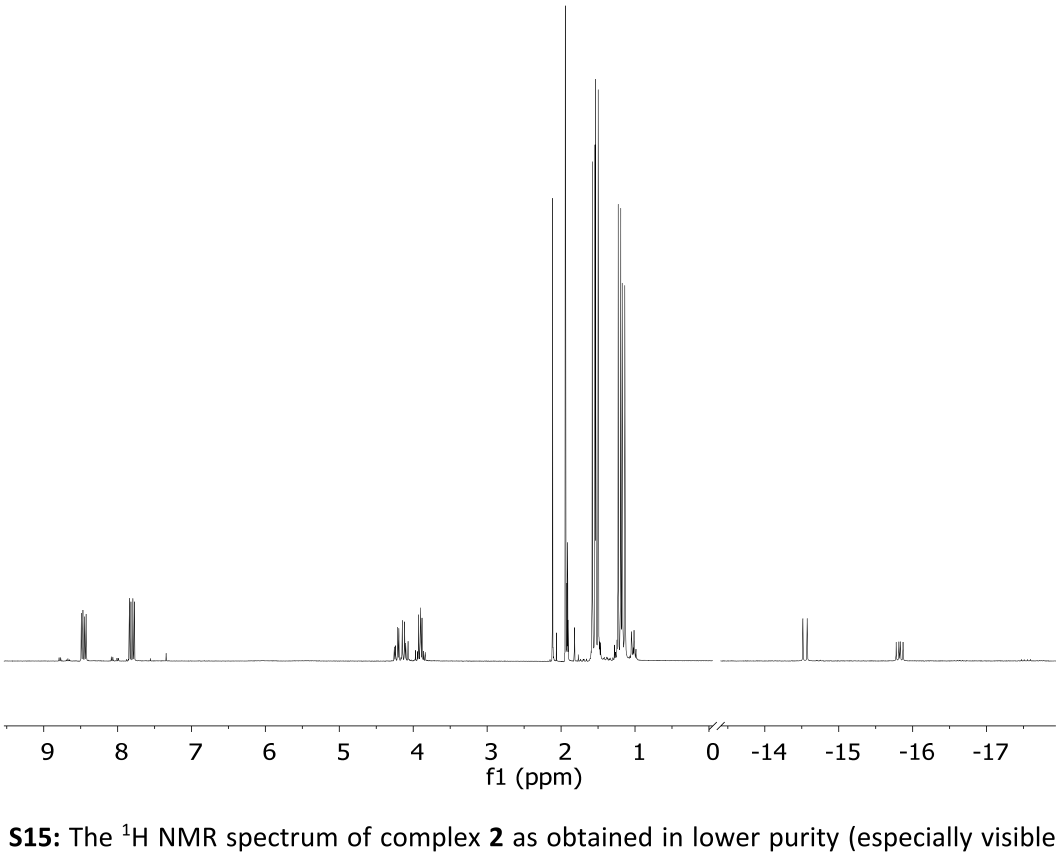

Figure S15: The ^{1}H NMR spectrum of complex **2** as obtained in lower purity (especially visible in the aromatic region) by skipping the second precipitation step.

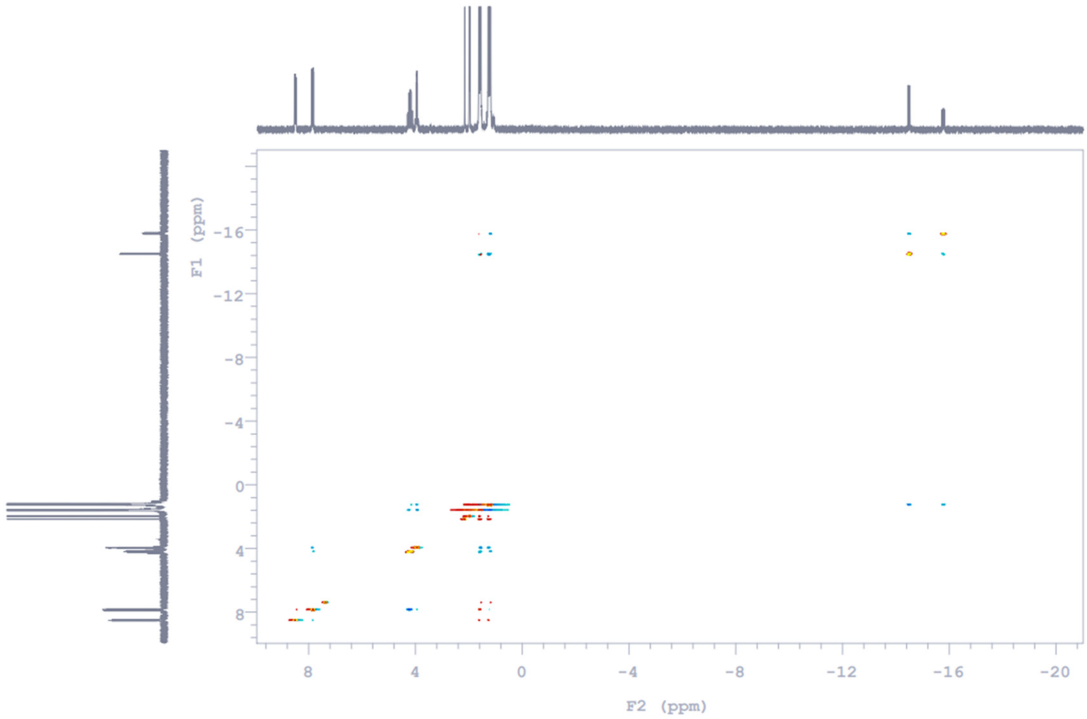

Figure S16: The Hadamard encoded NOESY NMR spectrum of complex **2** in acetonitrile-d_3 at 25 °C.

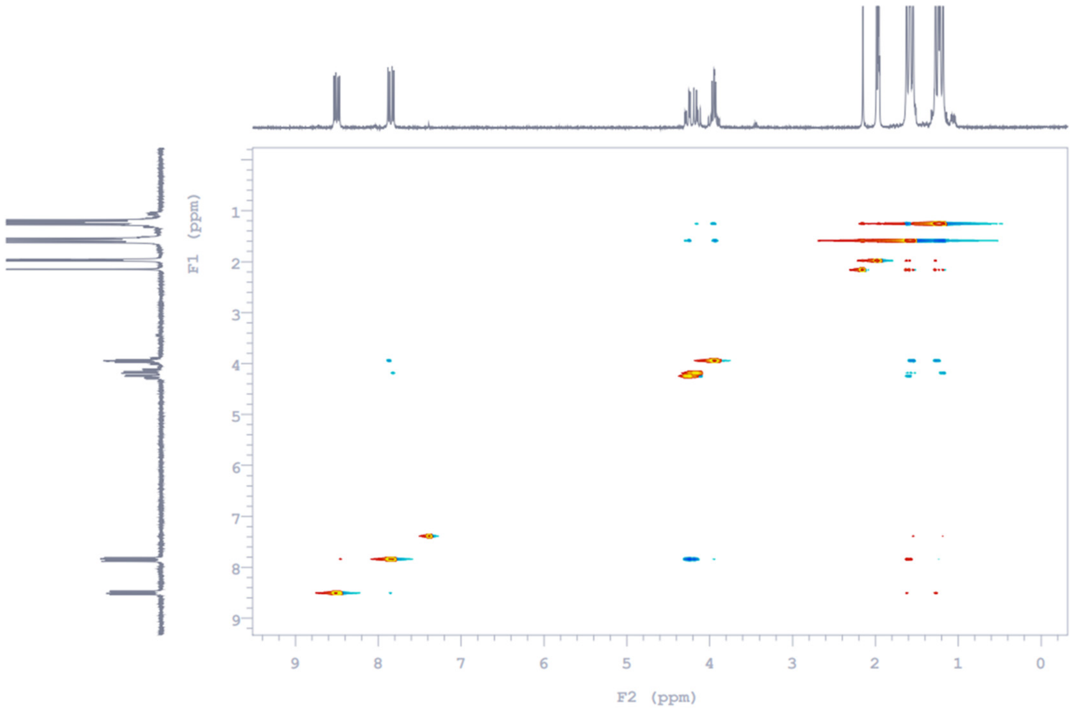

Figure S17: A zoom in of the Hadamard encoded NOESY NMR spectrum of complex **2** in acetonitrile-d_3 at 25 °C.

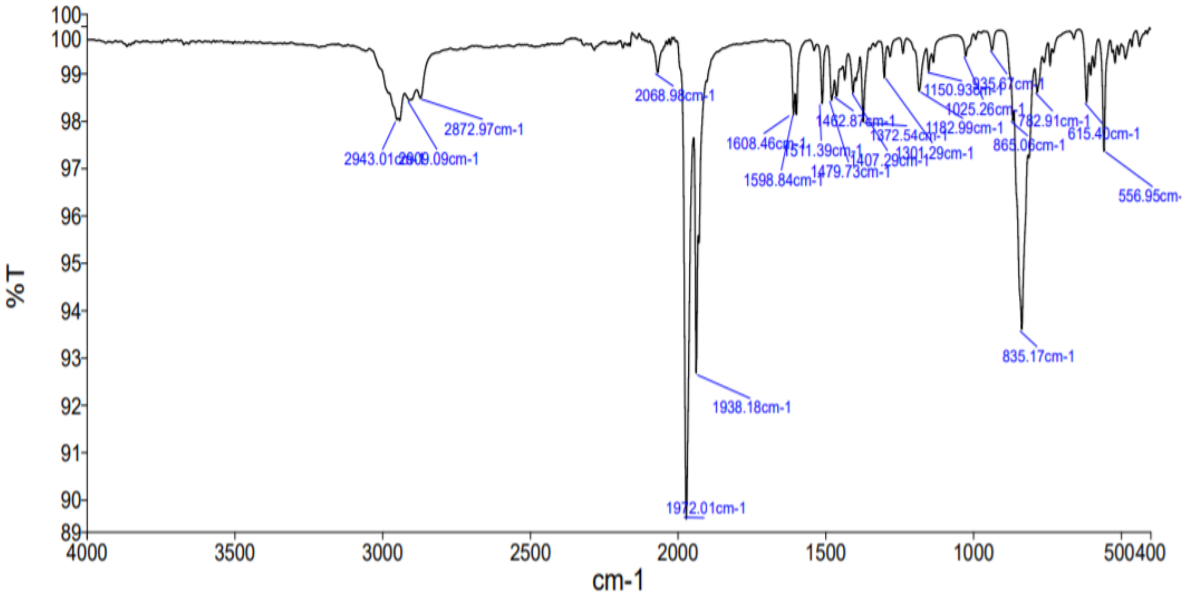

Figure S18: The ATR-IR spectrum of complex **2** measured as a solid under air.

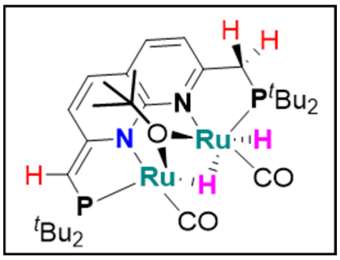

[Ru$_2$(^{tBu}PNNP*)H(μ-H)(μ-O*t*Bu)(CO)$_2$] (3) A suspension of complex **1** (99.4 mg, 0.128 mmol) in benzene (2.5 mL) was dropwise treated with a suspension of KO*t*Bu (28.8 mg, 0.257 mmol, 2 equiv) at ambient temperature under stirring. The resulting dark red/brown suspension was dried under a dynamic vacuum after stirring for 60 min. The resulting red solid was extracted with pentane (10 mL) and the red solution was then dried under a dynamic vacuum yielding complex **3** as a red solid (71.5 mg, 0.092 mmol, 72%, containing slight impurities by NMR analysis (see note)).

^{1}H NMR (400 MHz, THF-*d$_8$*, 298 K): δ = 6.97 (d, $^3J_{H,H}$ = 6.7 Hz, 1H), 6.53 (d, $^3J_{H,H}$ = 9.1 Hz, 1H), 6.46 (d, $^3J_{H,H}$ = 7.3 Hz, 1H), 6.38 (d, $^3J_{H,H}$ = 8.9 Hz, 1H), 4.20 (s, 1H), 3.45 (dd, $^2J_{H,H}$ = 16.8 Hz, $^2J_{H,P}$ = 8.7 Hz, 1H), 3.36 – 3.24 (m, 1H), 1.51 (d, $^3J_{H,P}$ = 13.3 Hz, 9H), 1.42 (d, $^3J_{H,P}$ = 13.0 Hz, 9H), 1.28 (overlapping doublets (dd), $J_{apparent}$ = 27.7 Hz, 13.5 Hz, 18H), 1.13 (s, 9H), -15.76 (d, $^2J_{H,P}$ = 31.0 Hz, 1H), -20.97 (dd, $^2J_{H,P}$ = 14.6 Hz, $^2J_{H,P}$ = 8.1 Hz, 1H).

^{13}C{^{1}H} NMR (101 MHz, THF-*d$_8$*, 298 K): δ = 205.1 (d, $^2J_{C,P}$ = 17.2 Hz), 203.5 (dd, $^2J_{C,P}$ = 14.2 Hz, $^4J_{C,P}$ = 7.8 Hz), 168.9 (d, $^2J_{C,P}$ = 15.9 Hz), 161.0 (s), 160.1 (d, $^2J_{C,P}$ = 3.9 Hz), 134.1 (s), 129.94 (s), 124.1 (d, $^3J_{C,P}$ = 15.6 Hz), 119.8 (s), 110.65 (d, $^3J_{C,P}$ = 9.7 Hz), 82.2 (d, $^1J_{C,P}$ = 43.0 Hz), 78.4 (s), 39.1 (d, $^1J_{C,P}$ = 20.4 Hz), 38.4 (d, $^1J_{C,P}$ = 26.9 Hz), 37.3 (d, $^1J_{C,P}$ = 16.7 Hz), 36.6 (d, $^1J_{C,P}$ = 19.1 Hz), 35.7 (d, $^1J_{C,P}$ = 29.4 Hz), 30.8 (s), 29.87 (d, $^2J_{C,P}$ = 3.9 Hz), 29.5 (d, $^2J_{C,P}$ = 4.7 Hz), 29.3 (d, $^2J_{C,P}$ = 3.0 Hz).

^{31}P{^{1}H} NMR (162 MHz, THF-*d$_8$*, 298 K): δ = 104.1 (dt, $J_{H,P}$ = 22.2 Hz, $J_{H,P}$ = 6.9 Hz), 91.4 (dd, J = 12.4 Hz, $J_{H,P}$ = 7.3 Hz).

ATR-IR (film, N$_2$ flow): v = 2960 (m), 2946 (m), 2897 (m), 2867 (m), 1923 (s), 1624 (w), 1546 (w), 1511 (w), 1414 (w), 1366 (w), 1319 (w), 1262 (w), 1181 (w), 1134 (w), 1020 (w), 866 (w), 834 (w), 613 (w) cm^{-1}.

Despite several attempts, due to its reactive nature we were unable to obtain satisfactory elemental analysis of complex **3**.

Note: Both conversion and purity in this reaction can vary with slight changes in reaction time and temperature. This reaction can also be performed in benzene, THF and toluene at ambient temperature using a 1:2 ratio of complex **1** and KO*t*Bu. An inseparable byproduct is observed in the experiments as can be seen in the ^{31}P{^{1}H} NMR spectrum (Figure S21). Complex **3** is unstable over time in (THF) solution (few days) and as a solid at ambient temperature in a matter of weeks. Therefore, complex **3** should be stored at -40 °C as a solid.

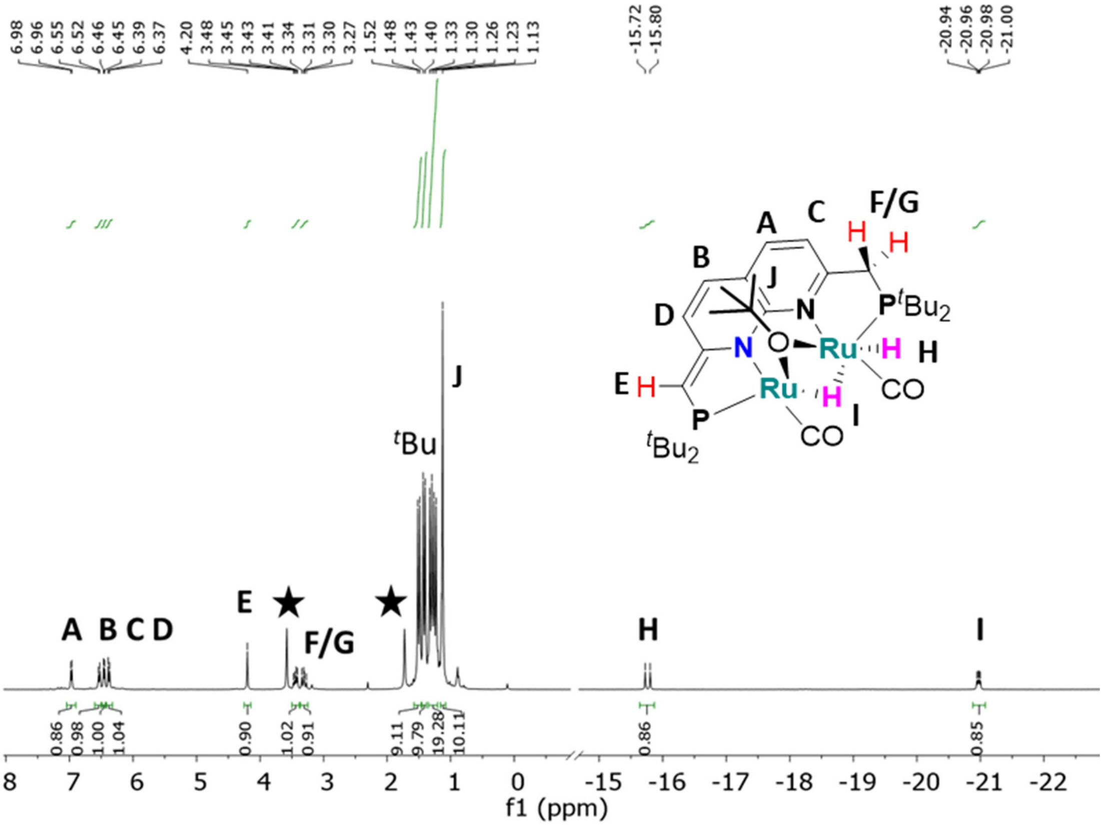

Figure S19: The ^{1}H NMR spectrum of complex **3** in THF-d_8 at 25 °C. Resonances marked with a star are assigned to THF-d_7.

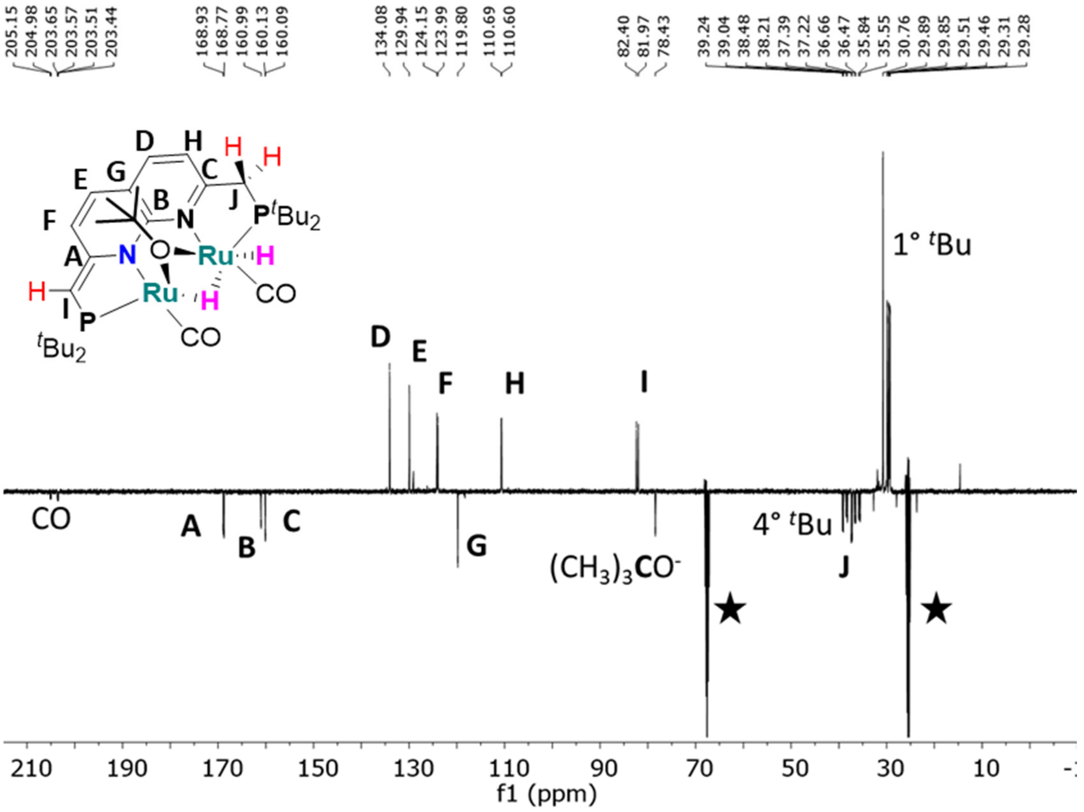

Figure S20: The ^{13}C-APT NMR spectrum of complex **3** in THF-d_8 at 25 °C. Resonances marked with a star are assigned to THF-d_8.

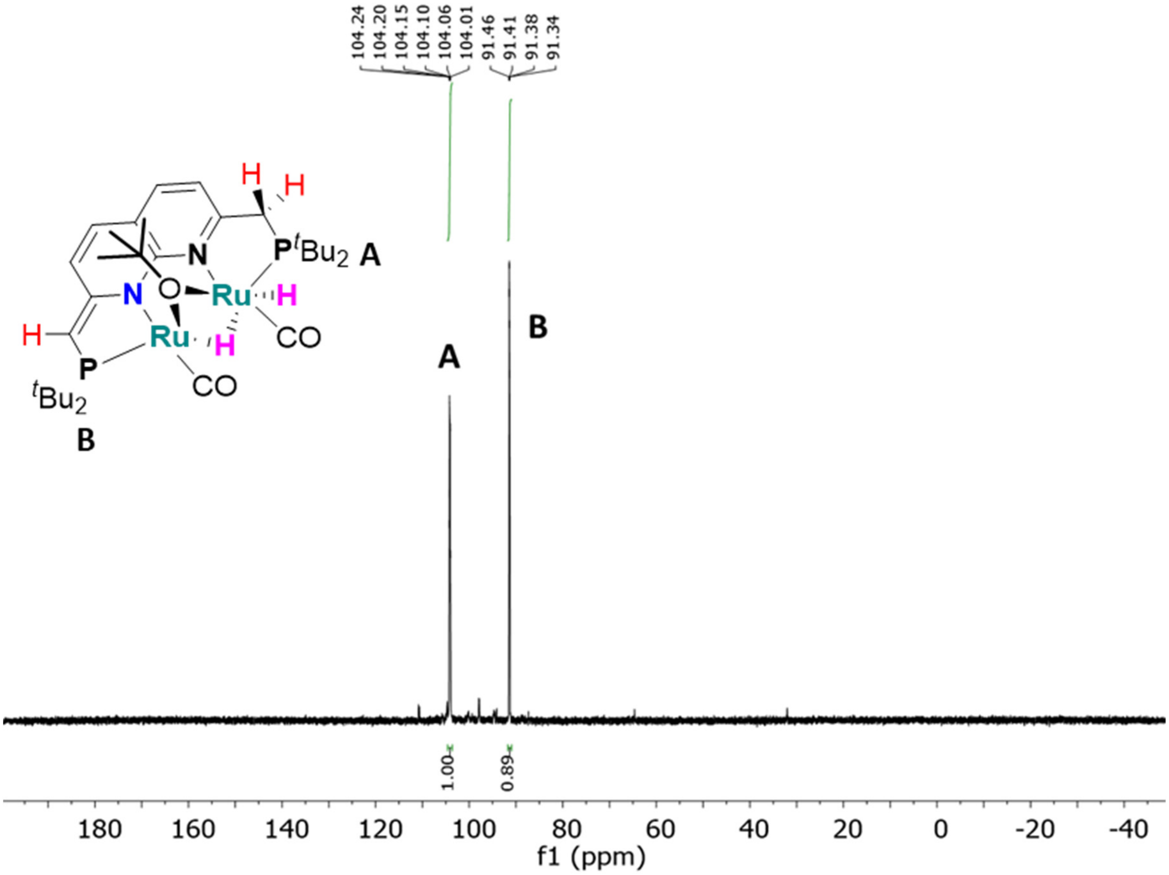

Figure S21: The $^{31}P\{^{1}H\}$ NMR spectrum of complex **3** in THF-d_8 at 25 °C.

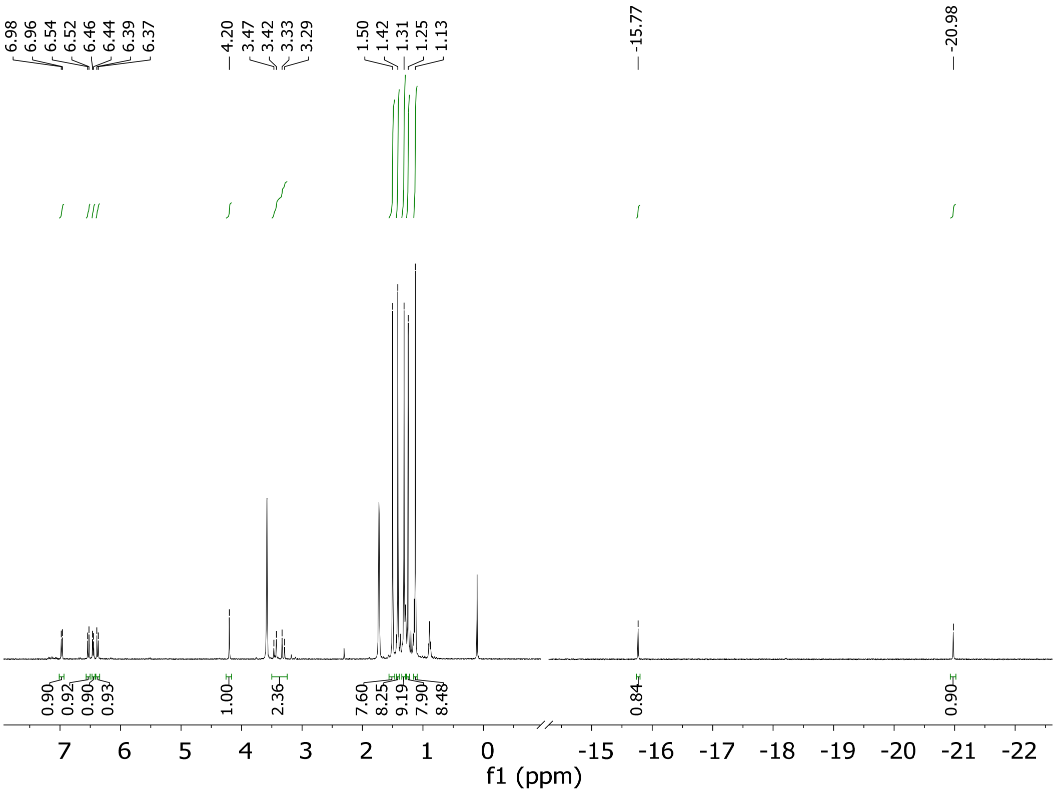

Figure S22: The $^{1}H\{^{31}P\}$ NMR spectrum of complex **3** in THF-d_8 at 25 °C.

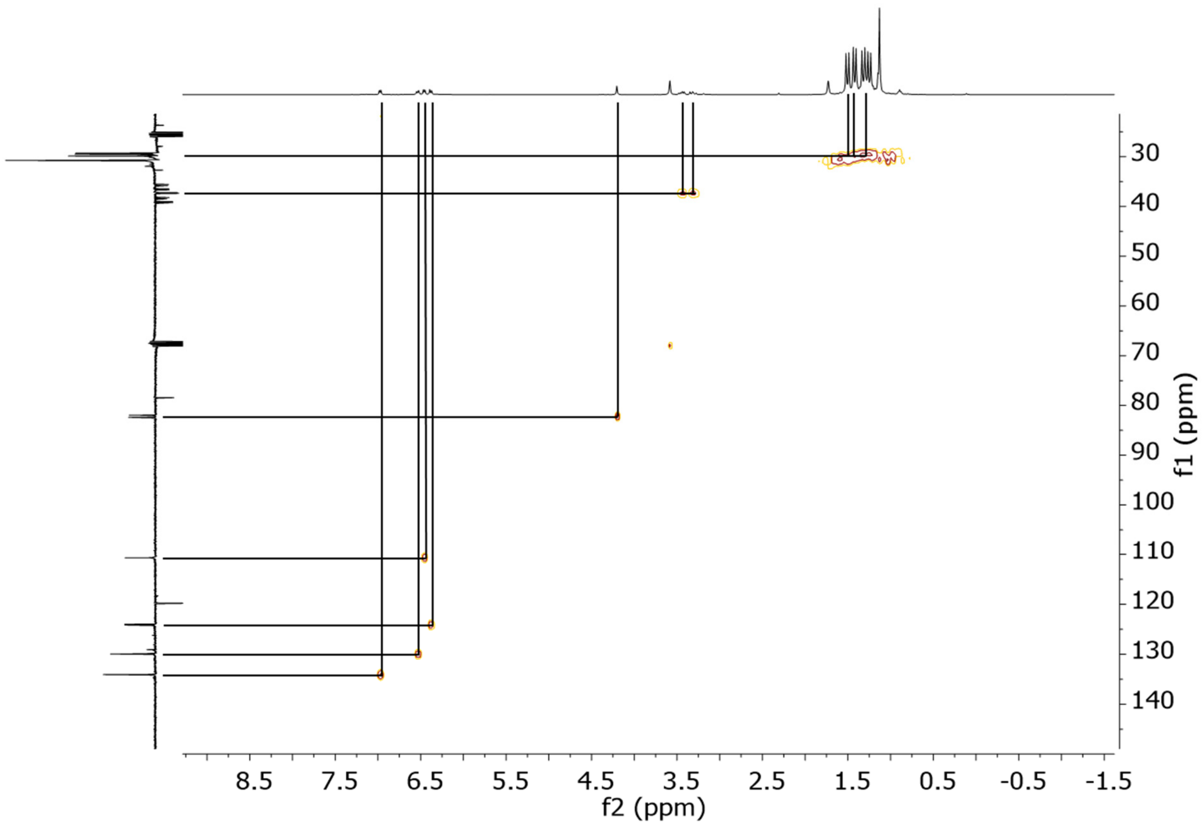

Figure S23: The ^{1}H-^{13}C HMQC NMR spectrum of complex **3** in THF-d_8 at 25 °C.

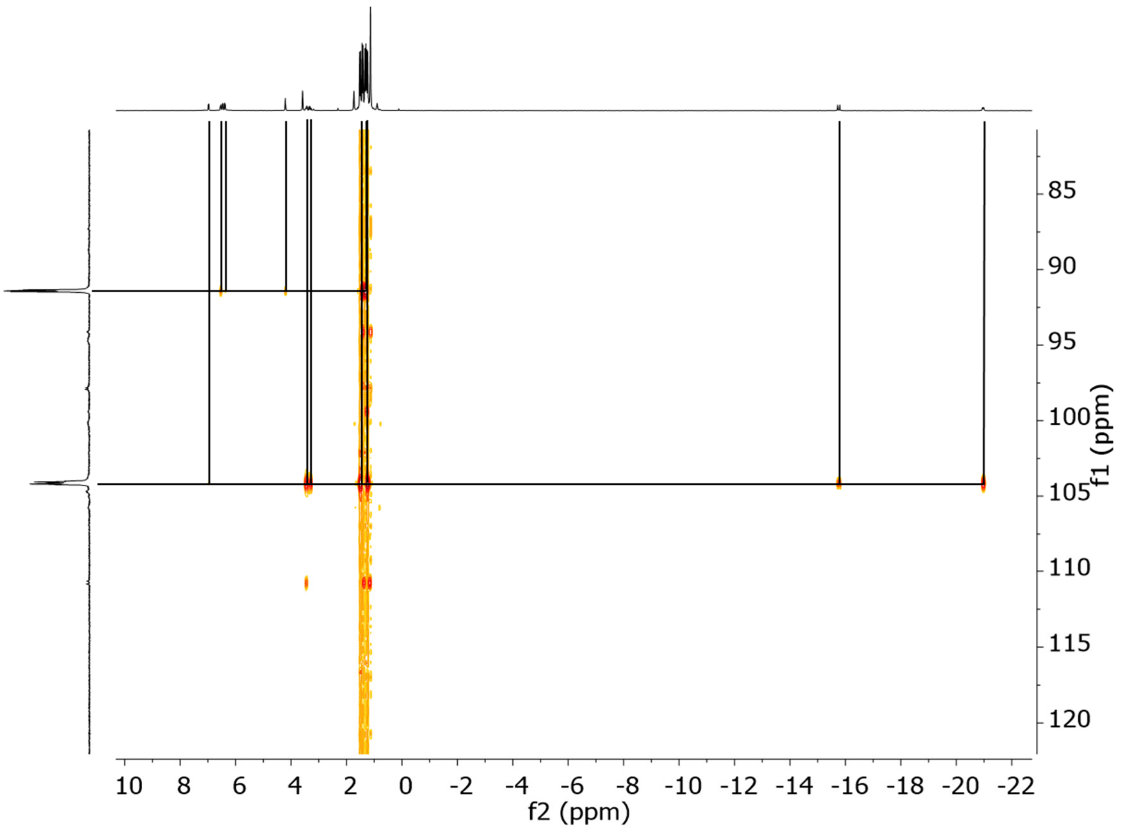

Figure S24: The ^{1}H-^{31}P HMBC NMR spectrum (multiple bond $J_{H,P}$-coupling = 8 Hz) of complex **3** in THF-d_8 at 25 °C.

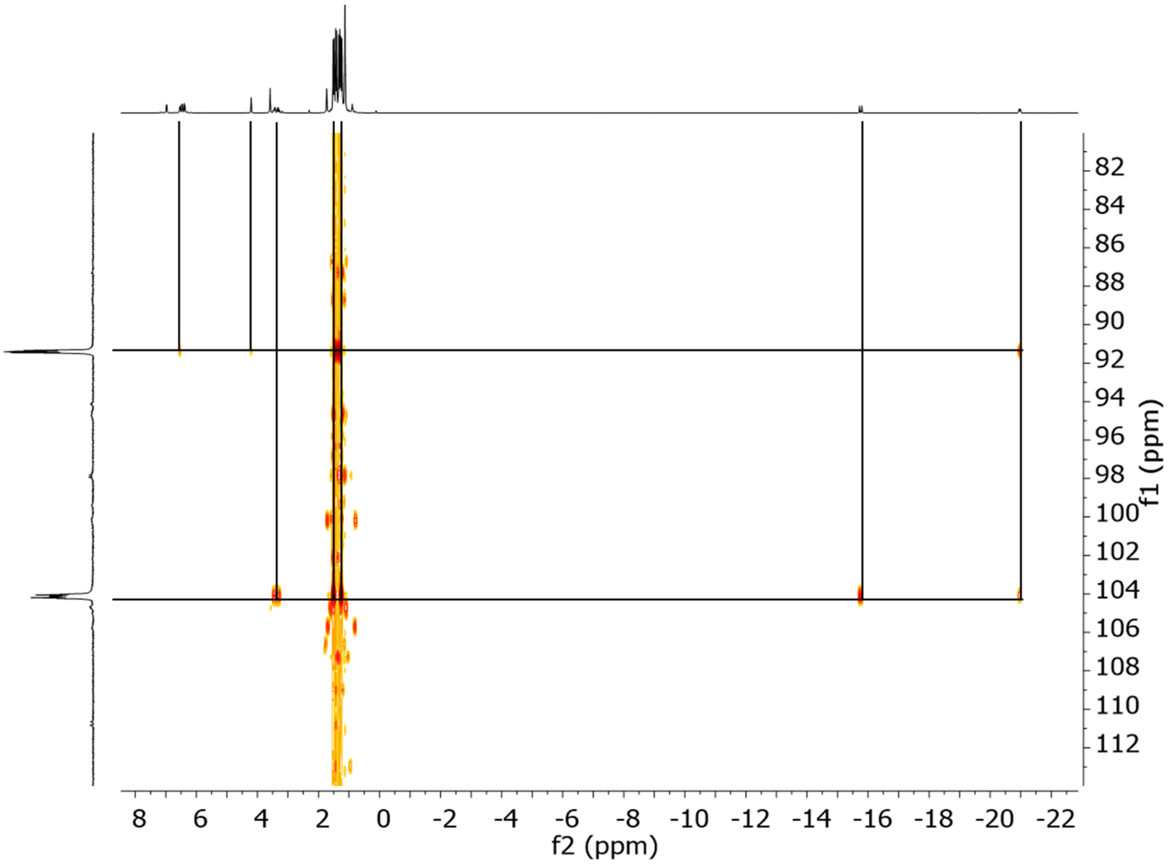

Figure S25: The ^{1}H-^{31}P HMBC NMR spectrum (multiple bond $J_{H,P}$-coupling = 12 Hz) of complex **3** in THF-d_8 at 25 °C.

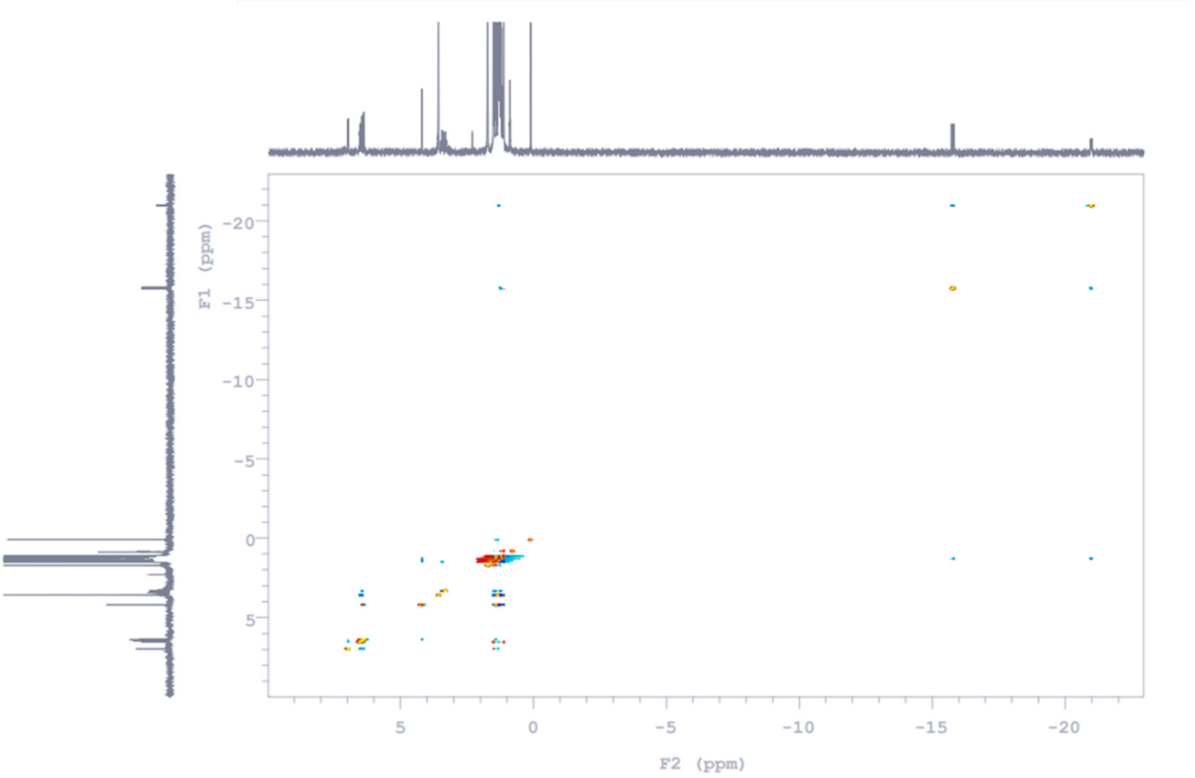

Figure S26: The Hadamard encoded NOESY NMR spectrum of complex **3** in THF-d_8 at 25 °C.

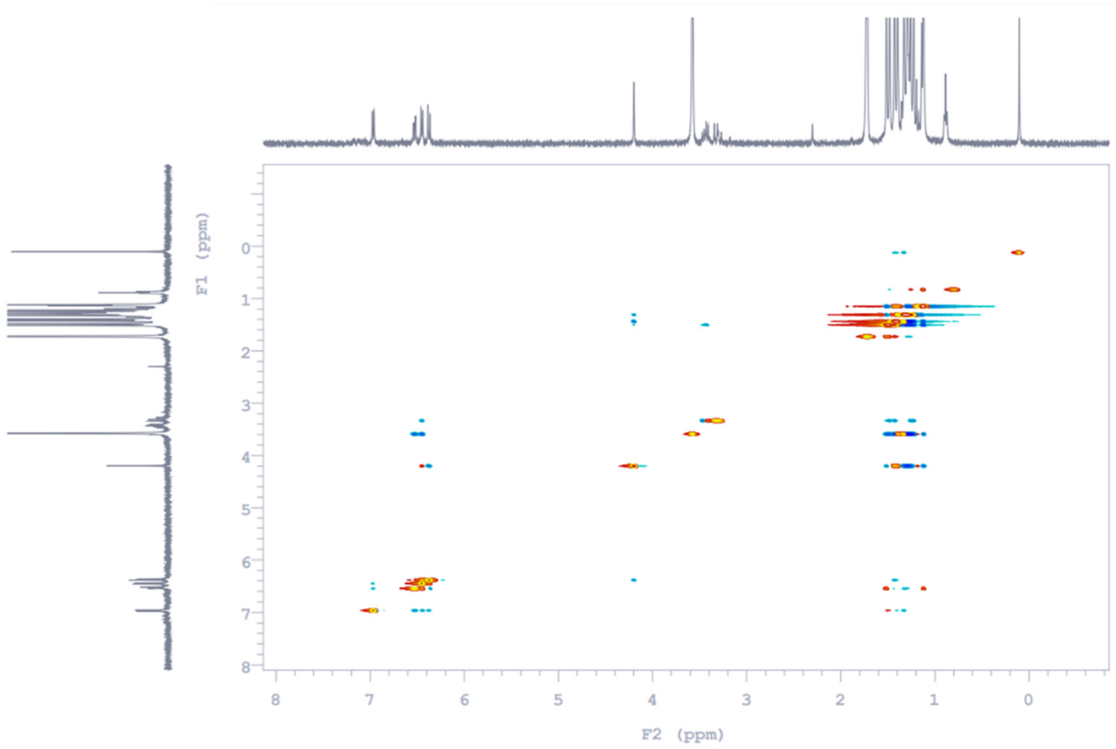

Figure S27: A zoom-in of the Hadamard encoded NOESY NMR spectrum of complex **3** in THF-d_8 at 25 °C.

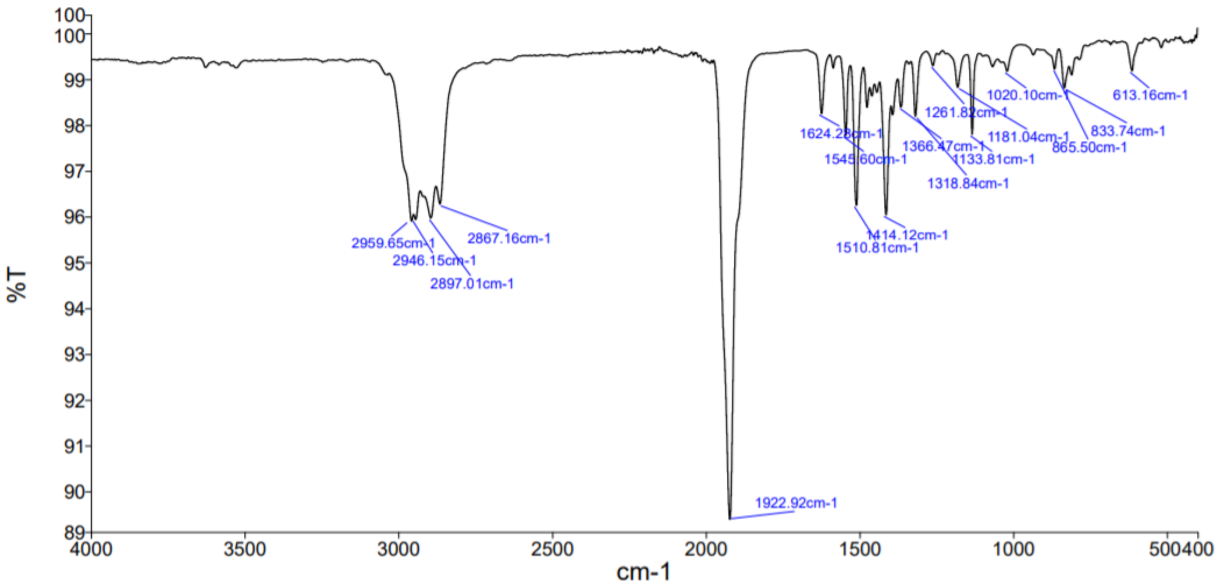

Figure S28: The ATR-IR spectrum of complex **3** measured as a film under N_2 flow.

In situ preparation of characterization of [Ru_2(^{tBu}PNNP)H_2(μ-H)$_2$(CO)$_2$] (4)

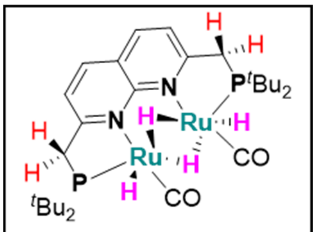

4
Proposed

A freshly prepared batch of complex **3** (11.6 mg, 14.9 µmol) was dissolved in THF (0.50 mL) and placed in a J-Young NMR tube. The red mixture was degassed by three freeze-pump-thaw cycles and filled with H_2 (1 atm) at ambient temperature. After 30 min, the solution had turned red brown and was analyzed by NMR spectroscopy, which showed full conversion of complex **3** and the formation of a single product based on ^{31}P and ^{1}H NMR spectroscopy. Complex **4** was not isolated*.

* **Note:** due to the reactive nature of complex **4**, we were unable to isolate and further characterize complex **4**. Solutions of complex **4** stored under a nitrogen or H_2 atmosphere are further converted over time (see Figure S31). Under a H_2 atmosphere complex **4** is converted to complex **5** through several unidentified intermediates. Under a nitrogen atmosphere complex **4** is partly converted to complex **5** and unidentified products. Benzene or toluene solutions of complex **3** placed under a H_2 atmosphere result in the formation of complex **5** after 24 or more hours. However, for these solutions intermediate complex **4** is not observed.

^{1}H NMR (400 MHz, THF-*H*$_8$, 298 K): δ = 8.30 (d, $^{3}J_{H,H}$ = 8.2 Hz, 2H), 7.77 (d, $^{3}J_{H,H}$ = 8.2 Hz, 2H), 3.94 (dd, $^{2}J_{H,H}$ = 17.3 Hz, $^{2}J_{H,P}$ = 9.5 Hz, 2H), 3.15 (s, 2H), 1.48 (d, $^{2}J_{H,P}$ = 12.7 Hz, 18H), 1.18 (d, $^{2}J_{H,P}$ = 12.4 Hz, 18H), -8.85 (d, $^{2}J_{H,H}$ = 60.6 Hz, 2H), -12.61 – -12.83 (m, 2H).

^{31}P{^{1}H} NMR (162 MHz, THF-*H*$_8$, 298 K): *δ* = 100.2 (s, 2P).

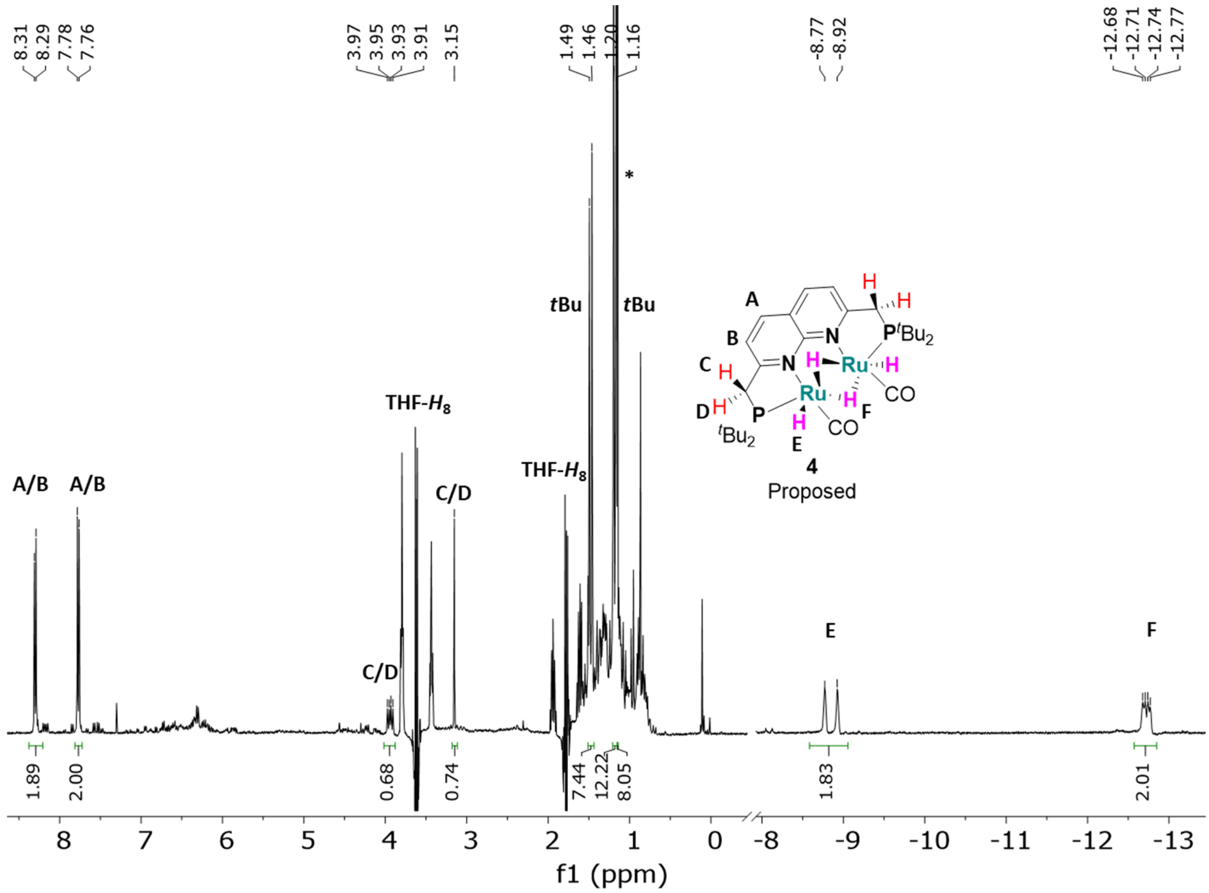

Figure 29: The ^{1}H NMR spectrum of complex **3** exposed to a H_2 atmosphere after 30 min in THF-H$_8$ at 25 °C. The resonance marked with a star is assigned to *tert*-butanol.

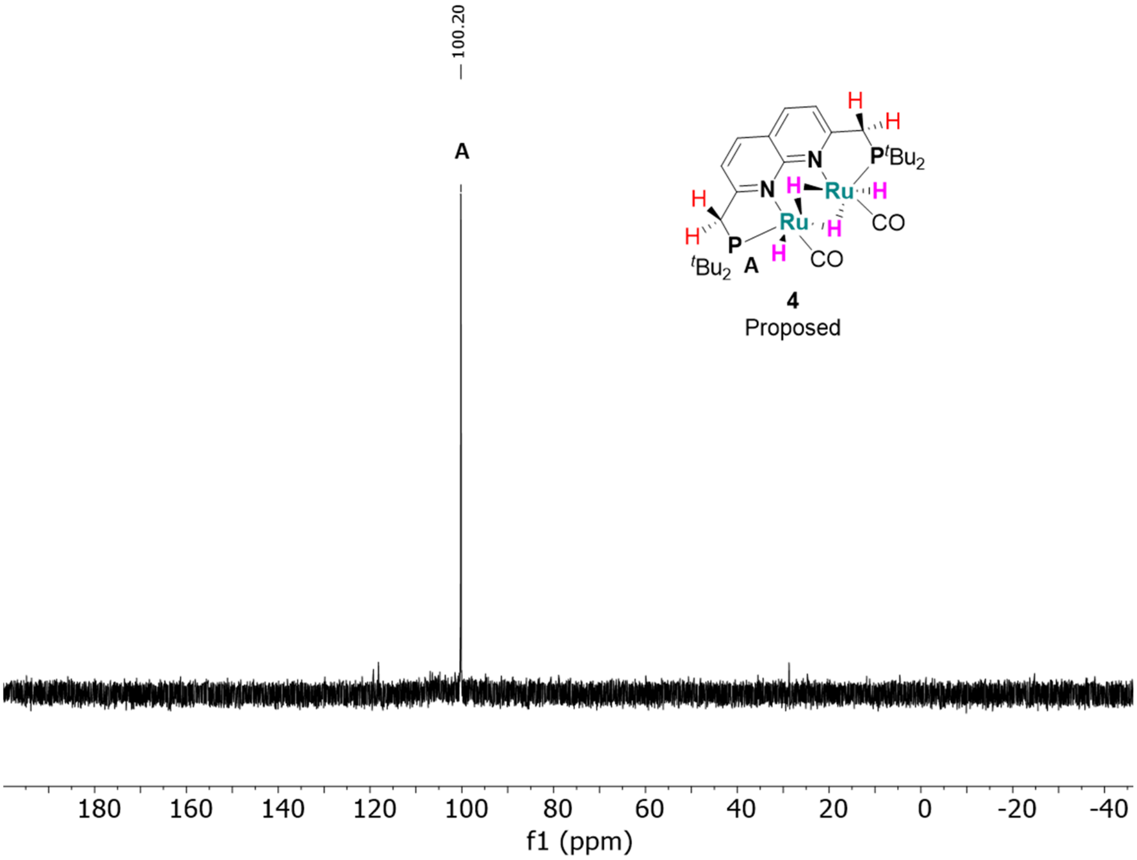

Figure 30: The $^{31}P\{^1H\}$ NMR spectrum of complex **3** exposed to a H_2 atmosphere after 30 min in THF-H_8 at 25 °C.

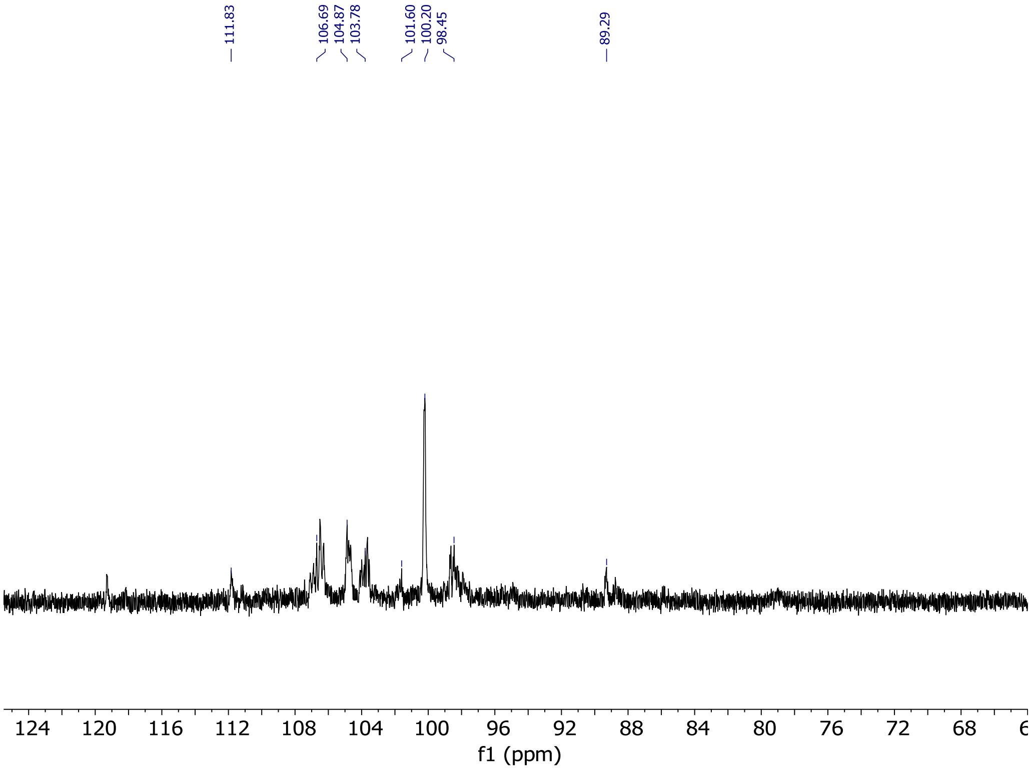

Figure S31: The $^{31}P\{^1H\}$ NMR spectrum of complex **3** exposed to a H_2 atmosphere after 120 min in THF-d_8 at 25 °C displaying the formation of multiple species.

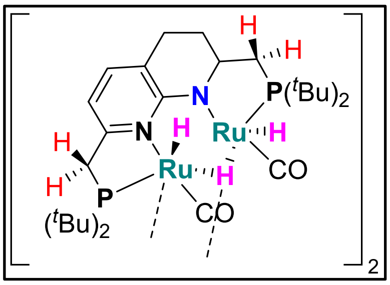

[(^{tBu}THPNNP)$Ru_2H_3(CO)_2]_2$ (5) A freshly prepared sample of complex **3** (40.4 mg, 0.047 mmol, small impurities as observed in NMR spectroscopy) was dissolved in THF (0.6 mL) and transferred to a J-Young NMR tube. The NMR tube was taken out of the glovebox and degassed using three freeze-pump-thaw cycles and backfilled with H_2 (1 atm). After storing the sample overnight, the sample was refilled with H_2 (1 atm). After 7 hours, the resulting brown solution was concentrated under a dynamic vacuum. The sample was again dissolved in THF (0.6 mL) and degassed using three freeze-pump-thaw cycles and backfilled with H_2 (1 atm). After storing the sample overnight, the resulting brown solution was concentrated under a dynamic vacuum. The mixture was dissolved in THF (0.3 mL) and hexane (10 mL) was added. The resulting solution was filtered and recrystallized in a freezer at -40 °C overnight. The supernatant was removed and the brown crystals were washed by hexane (2 mL) and then with pentane (5 x 3 mL) yielding complex **5** as a dark brown powder (13.6 mg, 41% from complex **3**).

^{1}H NMR (400 MHz, THF-*d$_8$*, 298 K): δ = 6.32 (d, $^3J_{H,H}$ = 6.6 Hz, 1H), 5.58 (d, $^3J_{H,H}$ = 6.8 Hz, 1H), 5.11 (dd, $^2J_{H,H}$ = 14.7 Hz, $^2J_{H,P}$ = 7.1 Hz, 1H), 3.80 – 3.71 (m, 1H), 3.26 (overlapping doublets (t), $J_{apparent}$ = 14.2 Hz, 1H), 2.30 – 2.21 (m, 2H), 1.55 – 1.46 (m, 2H), 1.32 (d, $^3J_{H,P}$ = 12.6 Hz, 9H), 1.12 (d, $^3J_{H,P}$ = 12.7 Hz, 9H), 0.86 (d, $^3J_{H,P}$ = 12.3 Hz, 9H), -13.39 (dd, $^2J_{H,P}$ = 46.6, $^2J_{H,P}$ = 24.2 Hz, 1H), -20.48 – 20.68 (overlapping signals (m), 2H).

^{1}H NMR (400 MHz, DCM-*d$_2$*, 298 K): δ = 6.33 (d, $^3J_{H,H}$ = 6.7 Hz, 1H), 5.84 (d, $^3J_{H,H}$ = 6.6 Hz, 1H), 5.00 (dd, $^2J_{H,H}$ = 14.9 Hz, $^2J_{H,P}$ = 7.1 Hz, 1H), 3.75 – 3.65 (m, 1H), 3.18 (overlapping doublets (t), $J_{apparent}$ = 14.0 Hz, 1H), 2.31 – 2.16 (m, 2H), 1.81 – 1.75 (m, 1H), 1.71 (d, $^3J_{H,P}$ = 13.3 Hz, 9H), 1.61 (dd, $^2J_{H,H}$ = 13.2 Hz, $^3J_{H,P}$ = 6.9 Hz, 1H), 1.51 – 1.43 (m, 2H), 1.30 (d, $^3J_{H,P}$ = 12.7 Hz, 9H), 1.09 (d, $^3J_{H,P}$ = 13.0 Hz, 9H), 0.82 (d, $^3J_{H,P}$ = 12.6 Hz, 9H), -13.40 (dd, $^2J_{H,P}$ = 47.7, $^2J_{H,P}$ = 24.8 Hz, 1H), -20.18 – -20.31 (m, 1H), -20.45 (dd, $^2J_{H,P}$ = 34.1, $^2J_{H,H}$ = 8.0 Hz, 1H).

^{13}C{^{1}H} NMR (101 MHz, THF-*d$_8$*, 298 K): δ = 206.7 (dd, J = 16.0 Hz, 7.0 Hz), 205.5 (dd, J = 13.6 Hz, 6.5 Hz), 165.1 (s), 157.7 (d, $^2J_{C,P}$ = 3.7 Hz), 132.8 (s), 116.72 (s), 103.7 (d, $^3J_{C,P}$ = 8.8 Hz), 64.4 (d, $^2J_{C,P}$ = 7.0 Hz), 38.8 – 38.2 (overlapping signals (m)), 36.8 (d, $^1J_{C,P}$ = 15.6 Hz), 36.5 – 35.9 (overlapping signals(m)), 34.0 (d, $^1J_{C,P}$ = 22.8 Hz), 32.6 (d, $^2J_{C,P}$ = 3.7 Hz), 30.4 (d, $^2J_{C,P}$ = 4.0 Hz), 29.7 (d, $^2J_{C,P}$ = 2.6 Hz), 29.4 (d, $^2J_{C,P}$ = 3.4 Hz), 27.5 (d, $^1J_{C,P}$ = 15.1 Hz), 26.3 (s).

^{13}C{^{1}H} NMR (101 MHz, DCM-*d$_2$*, 298 K): δ = 207.1 – 206.7 (m), 206.2 – 205.8 (m), 164.5 (s), 156.8 (d, $^2J_{C,P}$ = 3.6 Hz), 132.3 (s), 116.4 (s), 103.4 (d, $^3J_{C,P}$ = 8.7 Hz), 63.8 (d, $^2J_{C,P}$ = 6.5 Hz), 38.1 (dd, $^1J_{C,P}$ = 26.1 Hz, J = 3.3 Hz), 36.5 (d, $^1J_{C,P}$ = 15.0 Hz), 36.0 (d, $^1J_{C,P}$ = 17.0 Hz), 35.8 (s), 35.8 (d, $^1J_{C,P}$ = 19.4 Hz), 33.7 (d, $^1J_{C,P}$ = 22.9 Hz), 32.2 (d, $^2J_{C,P}$ = 3.7 Hz), 30.1 (d, $^2J_{C,P}$ = 3.9 Hz), 29.4 (d, $^2J_{C,P}$ = 2.6 Hz), 29.2 (d, $^2J_{C,P}$ = 3.3 Hz), 27.3 (d, $^1J_{C,P}$ = 15.1 Hz), 26.0 (s).

^{31}P{^{1}H} NMR (162 MHz, THF-*d$_8$*, 298 K): δ = 108.6 (d, $^2J_{H,P}$ = 31.3 Hz), 106.7 (d, J = 14.2 Hz).

^{31}P{^{1}H} NMR (162 MHz, DCM-*d$_2$*, 298 K): δ = 106.7 (d, J = 29.8 Hz), 105.0 (d, J = 13.9 Hz).

Despite several attempts, we were unable to obtain satisfactory elemental analysis of a spectroscopically clean sample of complex **5**.

ATR-IR (film, under N_2 flow): v = 2945 (m), 2925 (m), 2899 (m), 2866 (m), 2096 (w), 1950 (s), 1925 (m), 1897 (m), 1752 (w), 1608 (w), 1559 (w), 1469 (w), 1388 (w), 1366 (w), 1302 (w), 1251 (w), 1181 (w), 1097 (w), 1069 (w), 838 (w), 820 (w), 609 (w), 581 (w), 491 (w) cm^{-1}. A broad signal is present that could indicate a terminal hydride stretch at v = ~2100 cm^{-1}.

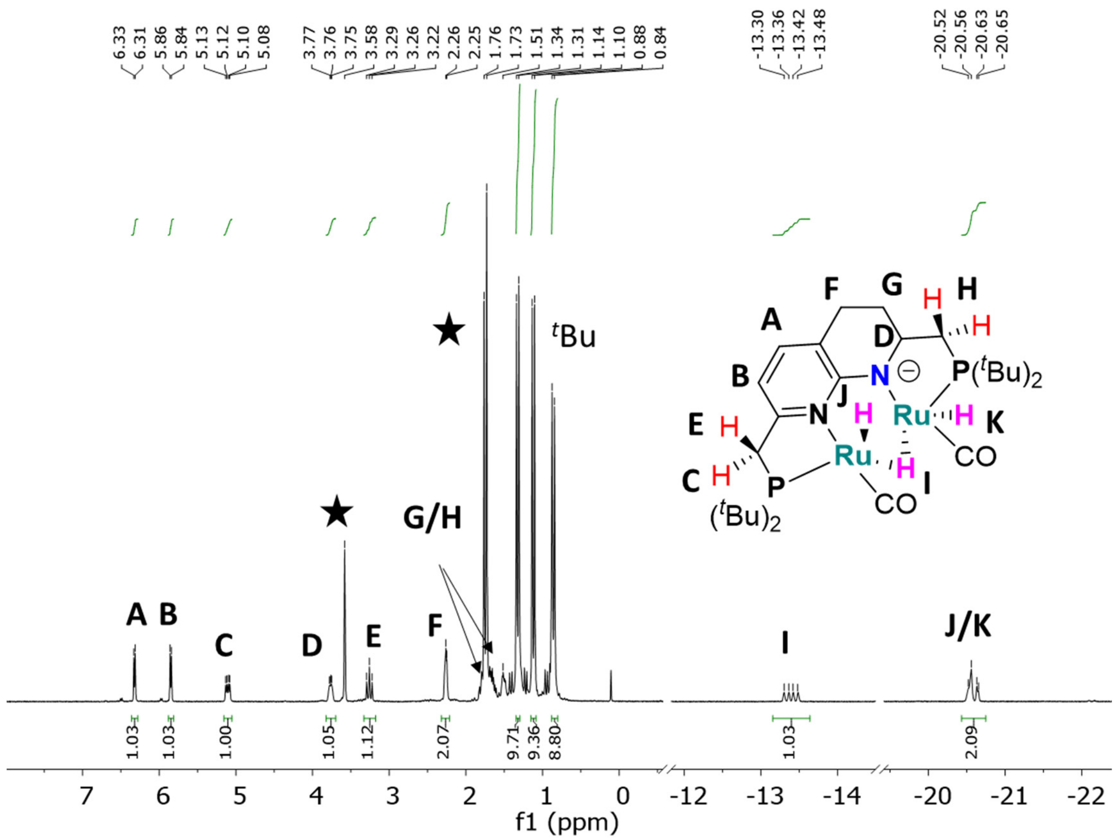

Figure S32: The ^{1}H NMR spectrum of complex **5** in THF-d_8 at 25 °C. Resonances marked with a star are assigned to THF-d_7.

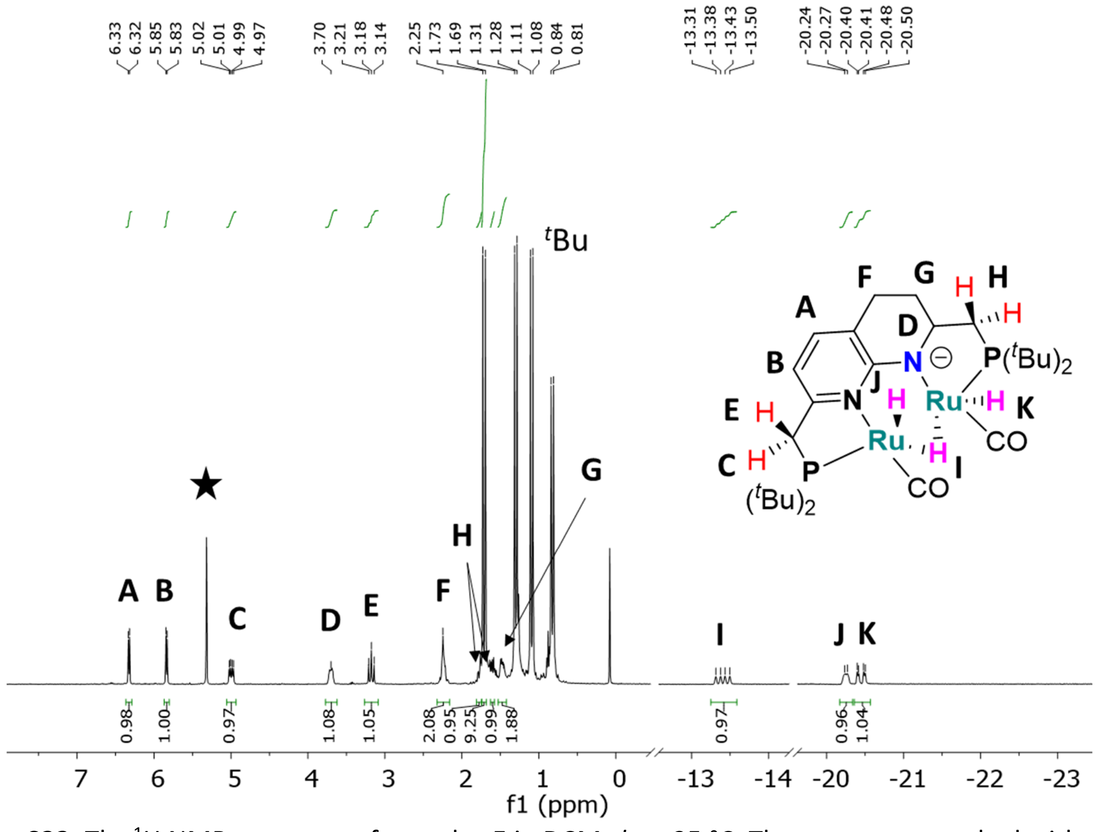

Figure S33: The ^{1}H NMR spectrum of complex **5** in DCM-d_2 at 25 °C. The resonance marked with a star is assigned to DCM-d_1.

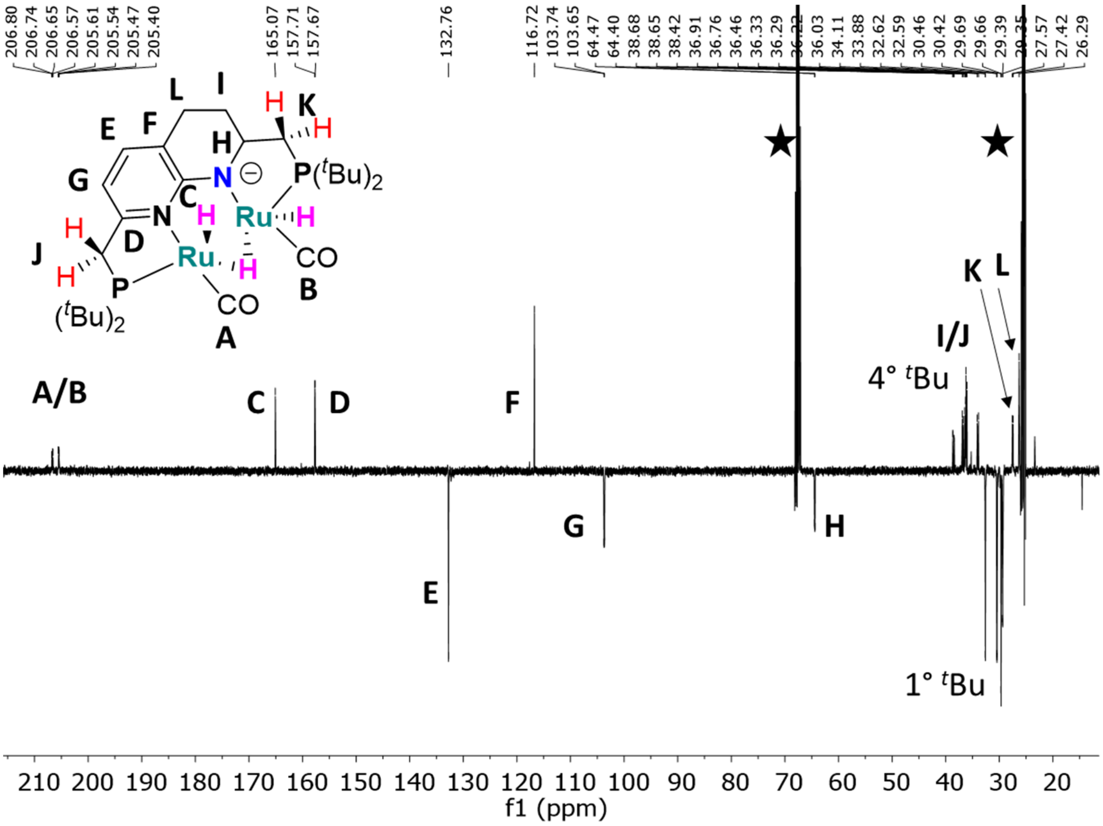

Figure S34: The ^{13}C-APT NMR spectrum of complex **5** in THF-d_8 at 25 °C. Resonances marked with a star are assigned to THF-d_8.

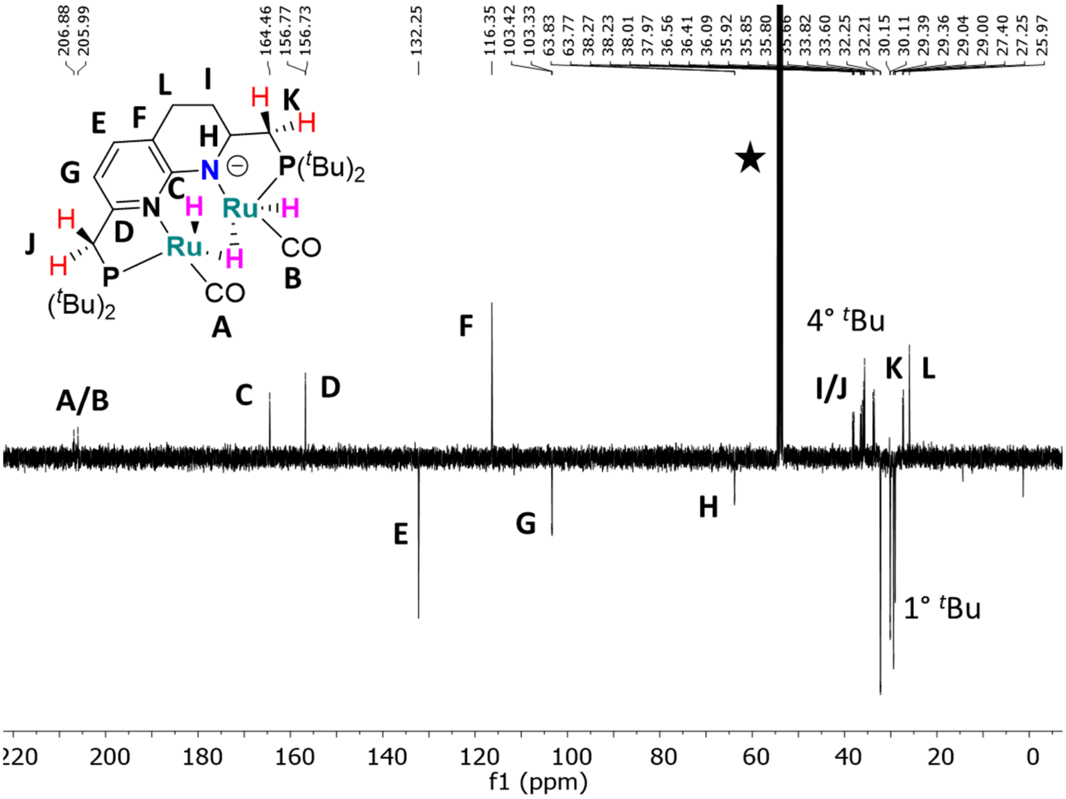

Figure S35: The ^{13}C-APT NMR spectrum of complex **5** in DCM-d_2 at 25 °C. The resonance marked with a star is assigned to DCM-d_2.

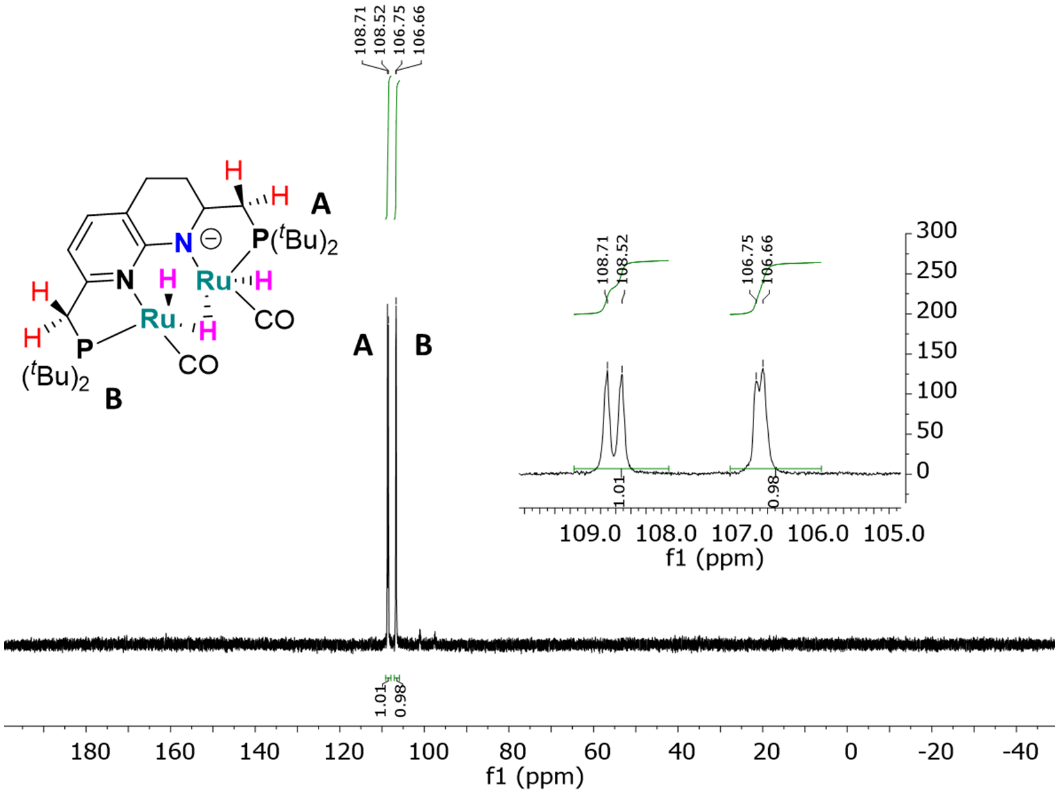

Figure S36: The $^{31}P\{^{1}H\}$ NMR spectrum of complex **5** in THF-d_8 at 25 °C.

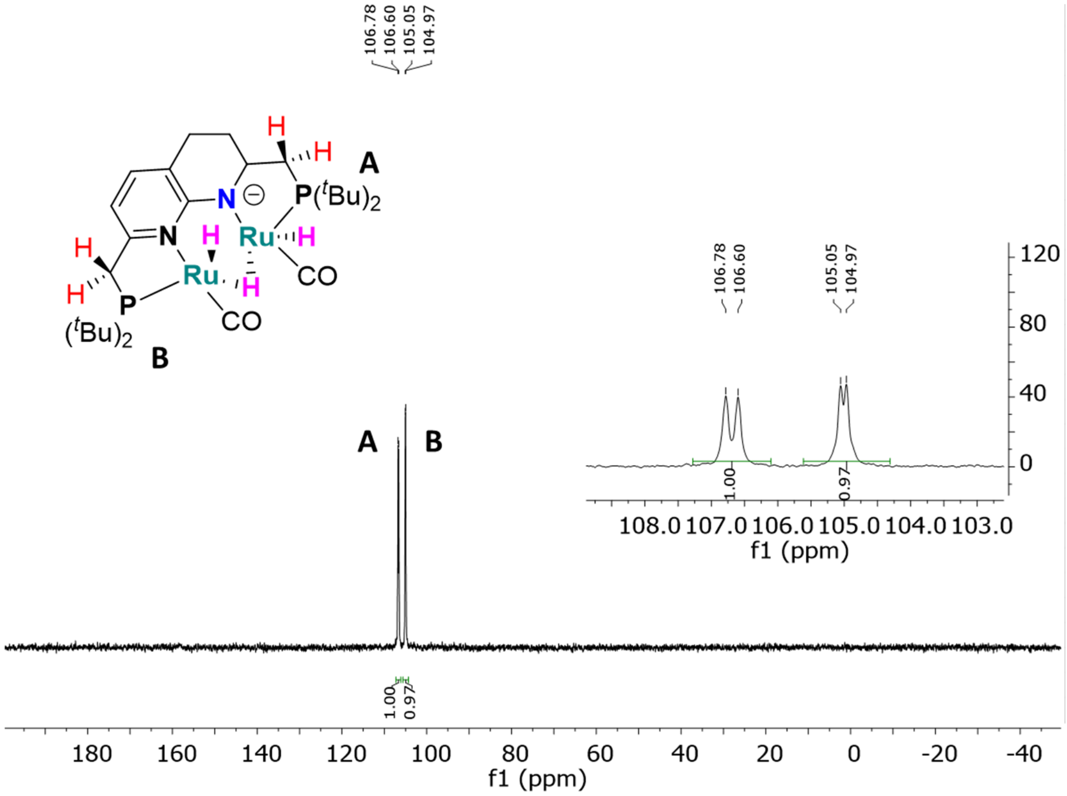

Figure S37: The $^{31}P\{^{1}H\}$ NMR spectrum of complex **5** in DCM-d_2 at 25 °C.

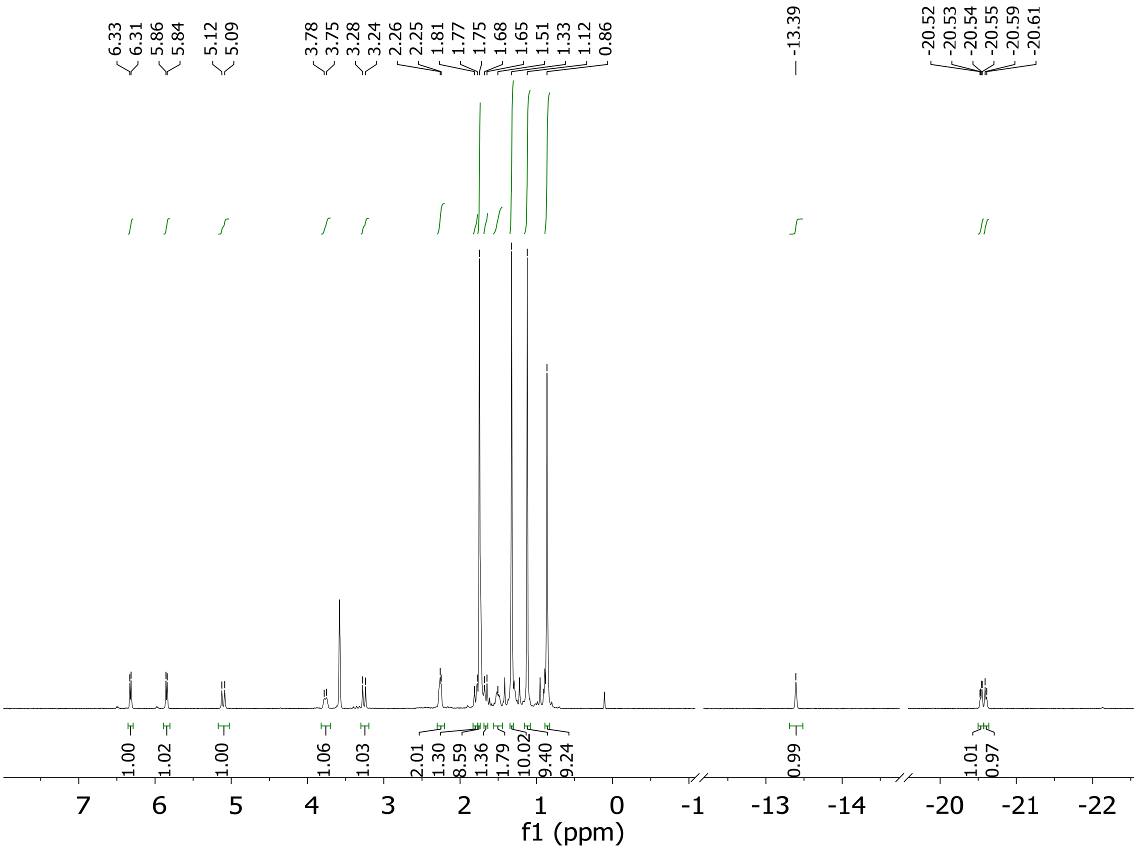

Figure S38: The $^{1}H\{^{31}P\}$ NMR spectrum of complex **5** in THF-d_8 at 25 °C.

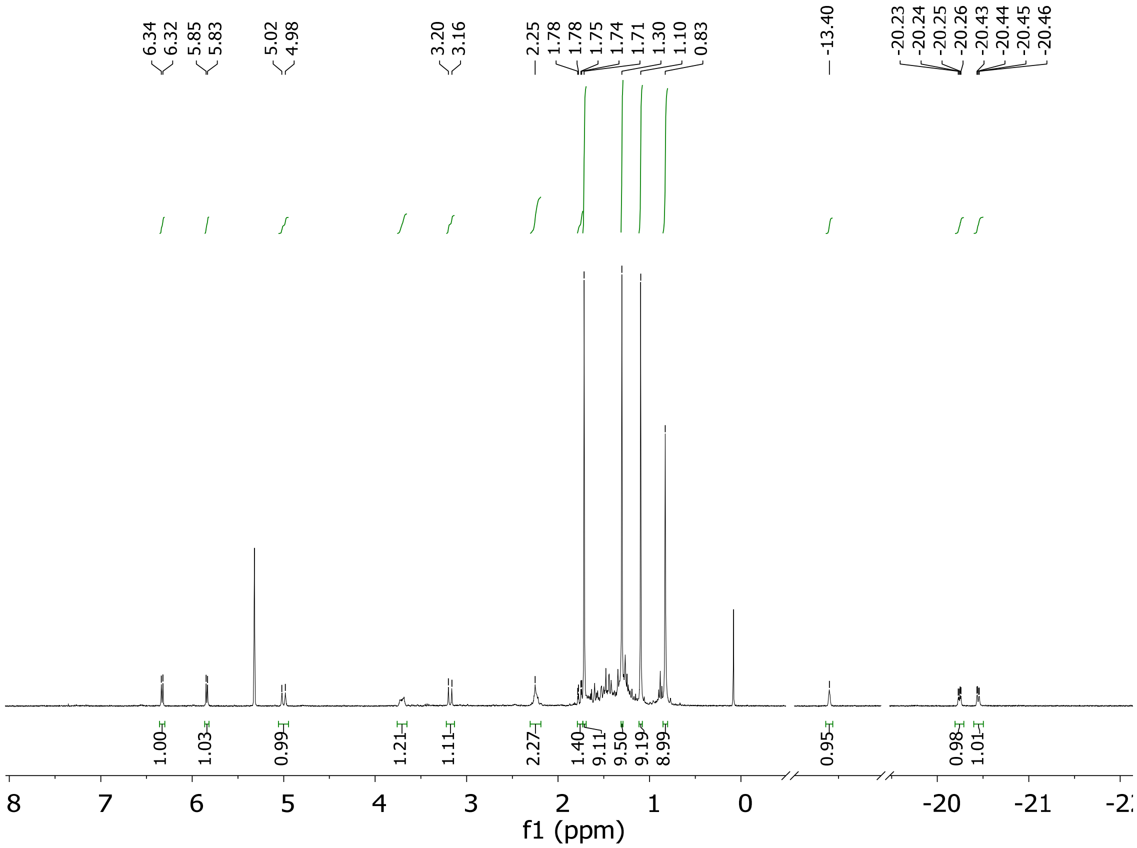

Figure S39: The $^{1}H\{^{31}P\}$ NMR spectrum of complex **5** in DCM-d_2 at 25 °C.

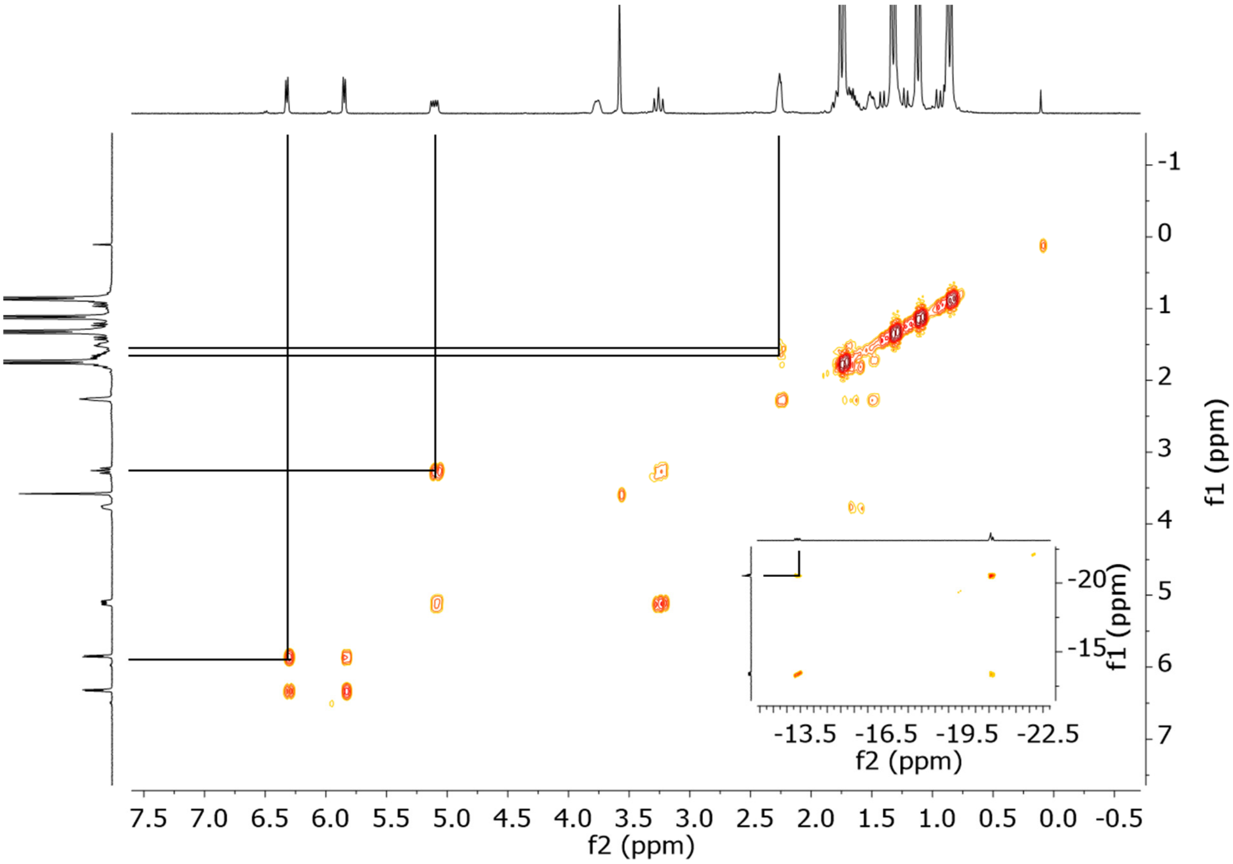

Figure S40: The COSY NMR spectrum of complex **5** in THF-d_8 at 25 °C.

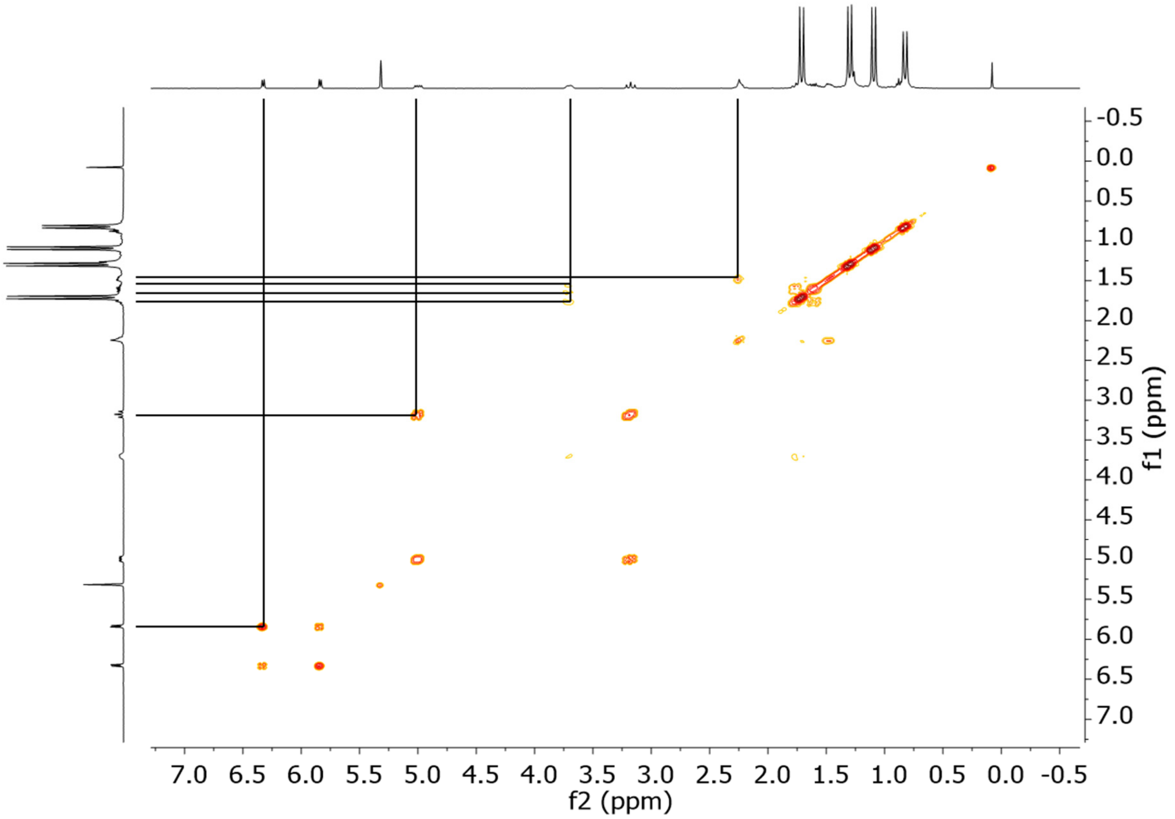

Figure S41: The COSY NMR spectrum of complex **5** in DCM-d_2 at 25 °C.

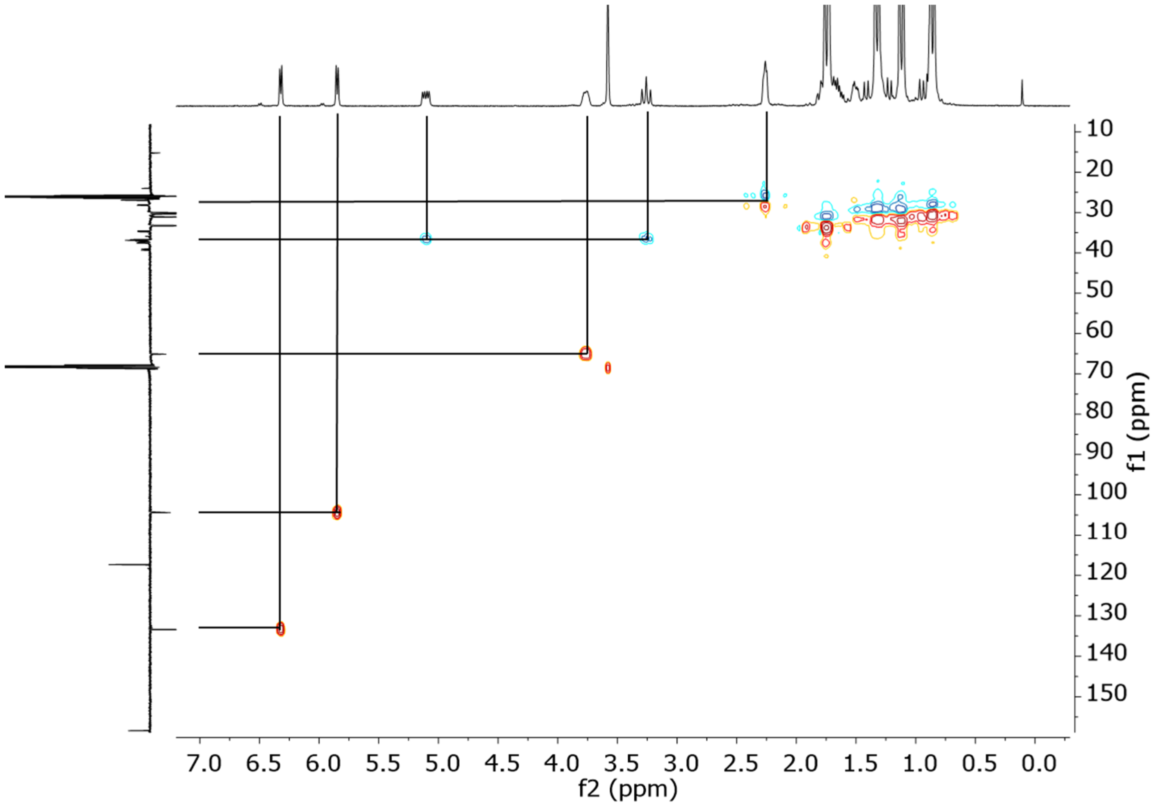

Figure S42: The ^{1}H-^{13}C HSQC NMR spectrum of complex **5** in THF-d_8 at 25 °C.

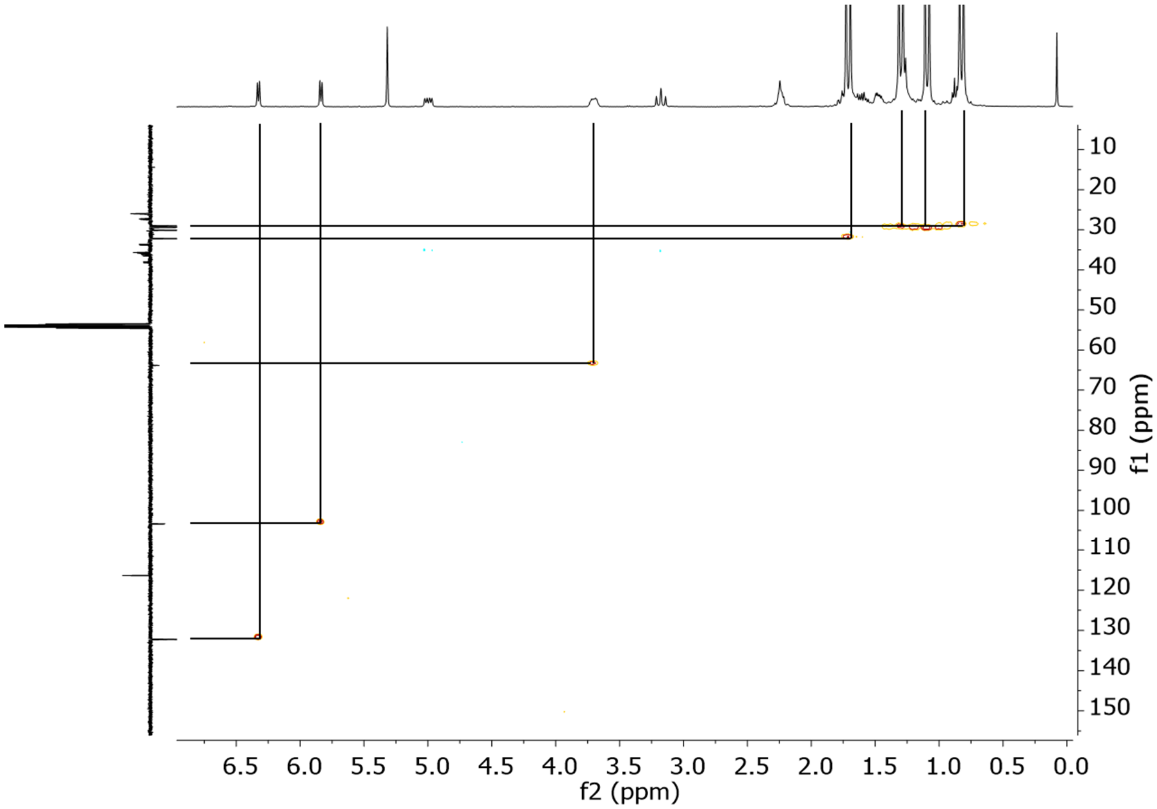

Figure S43: The ^{1}H-^{13}C HSQC NMR spectrum of complex **5** in DCM-d_2 at 25 °C.

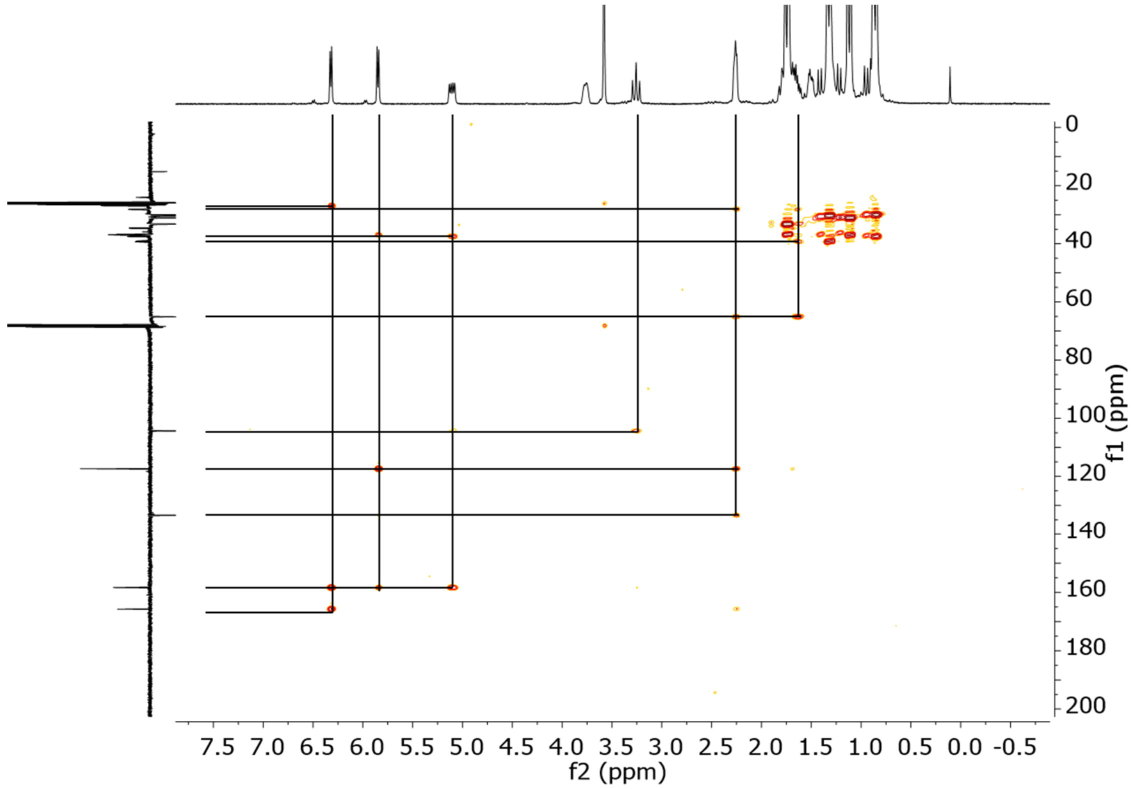

Figure S44: The ^{1}H-^{13}C HMBC NMR spectrum of complex **5** in THF-d_8 at 25 °C.

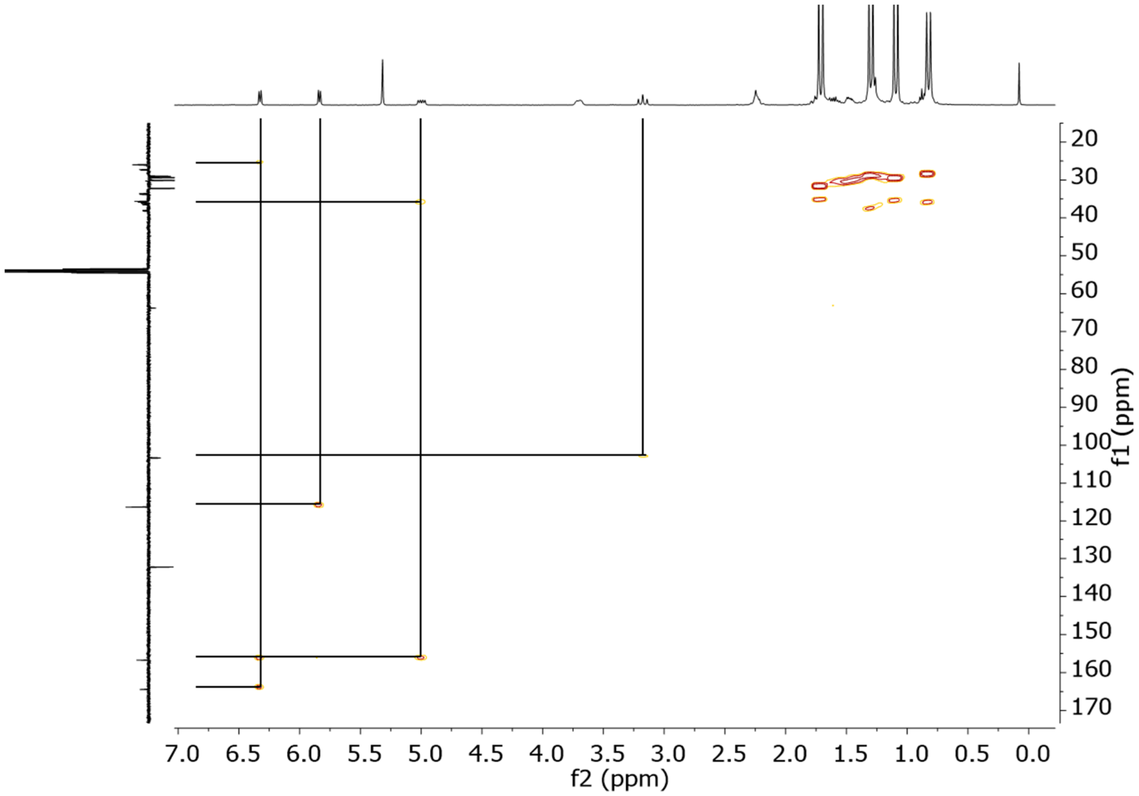

Figure S45: The ^{1}H-^{13}C HMBC NMR spectrum of complex **5** in DCM-d_2 at 25 °C.

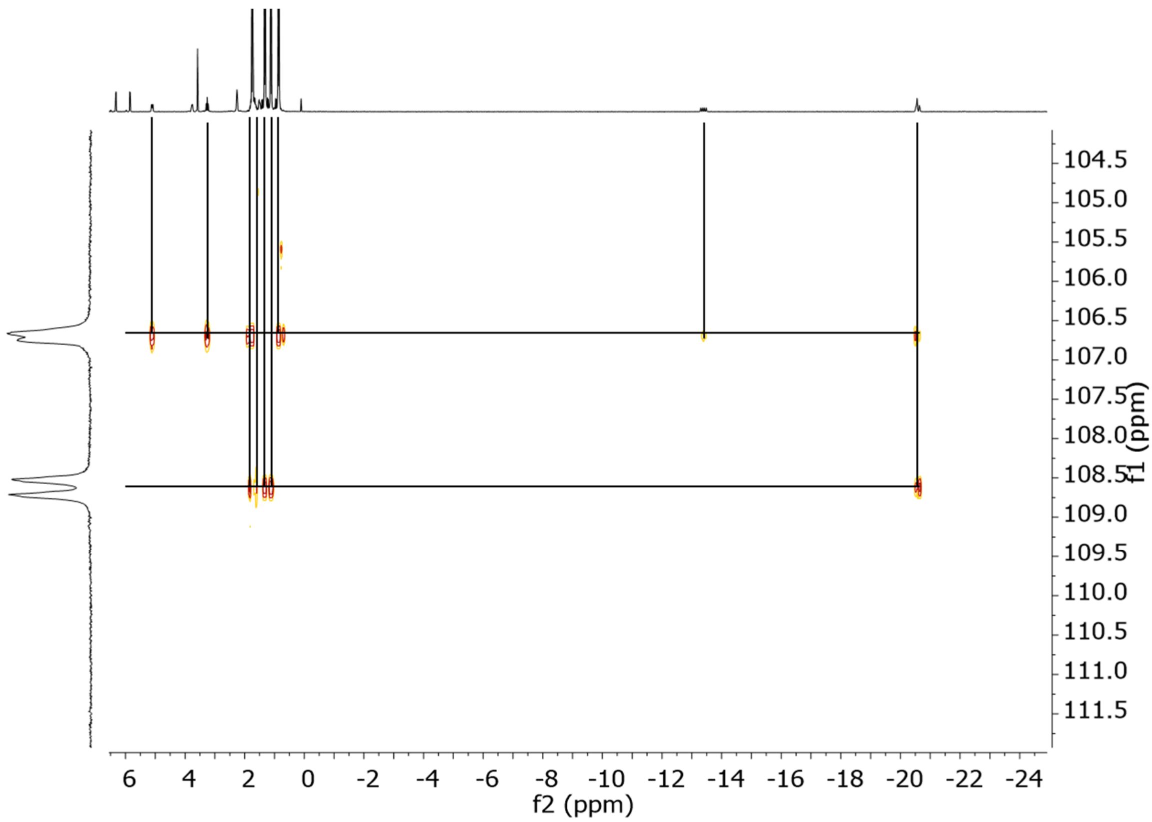

Figure S46: The ^{1}H-^{31}P HMBC NMR spectrum of complex **5** in THF-d_8 at 25 °C.

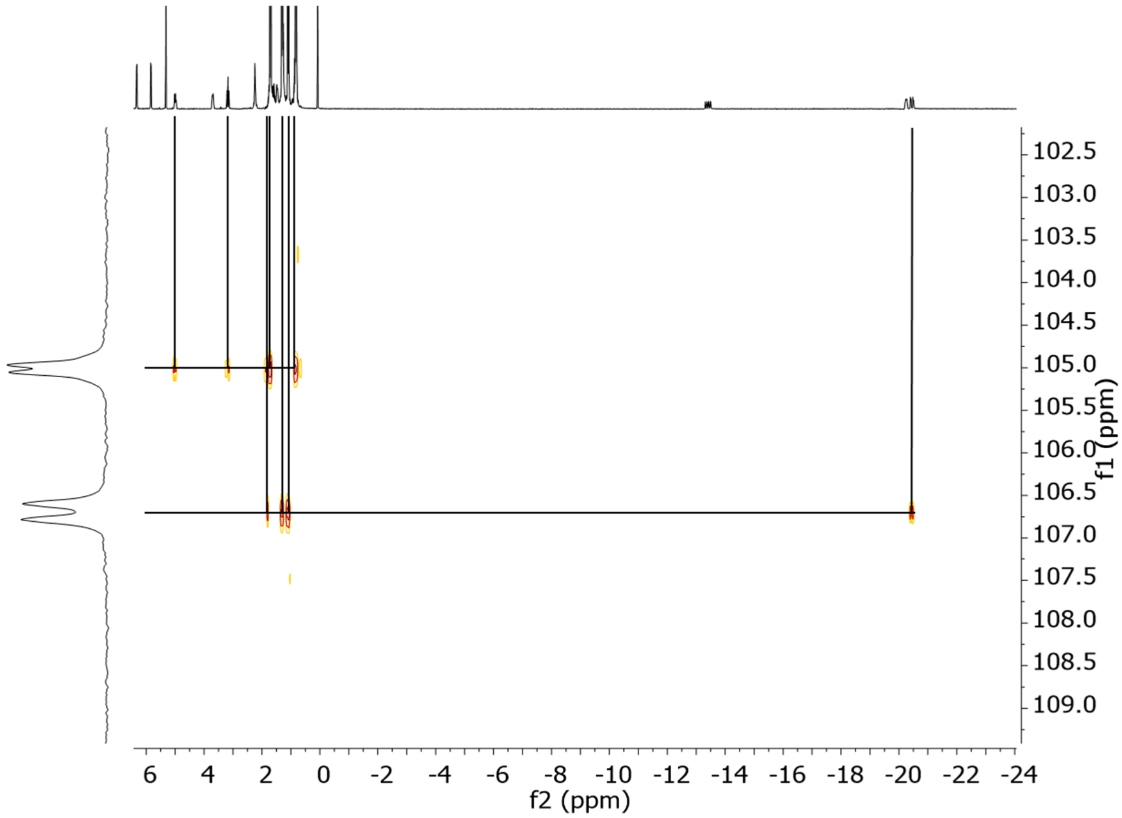

Figure S47: The ^{1}H-^{31}P HMBC NMR spectrum of complex **5** in DCM-d_2 at 25 °C.

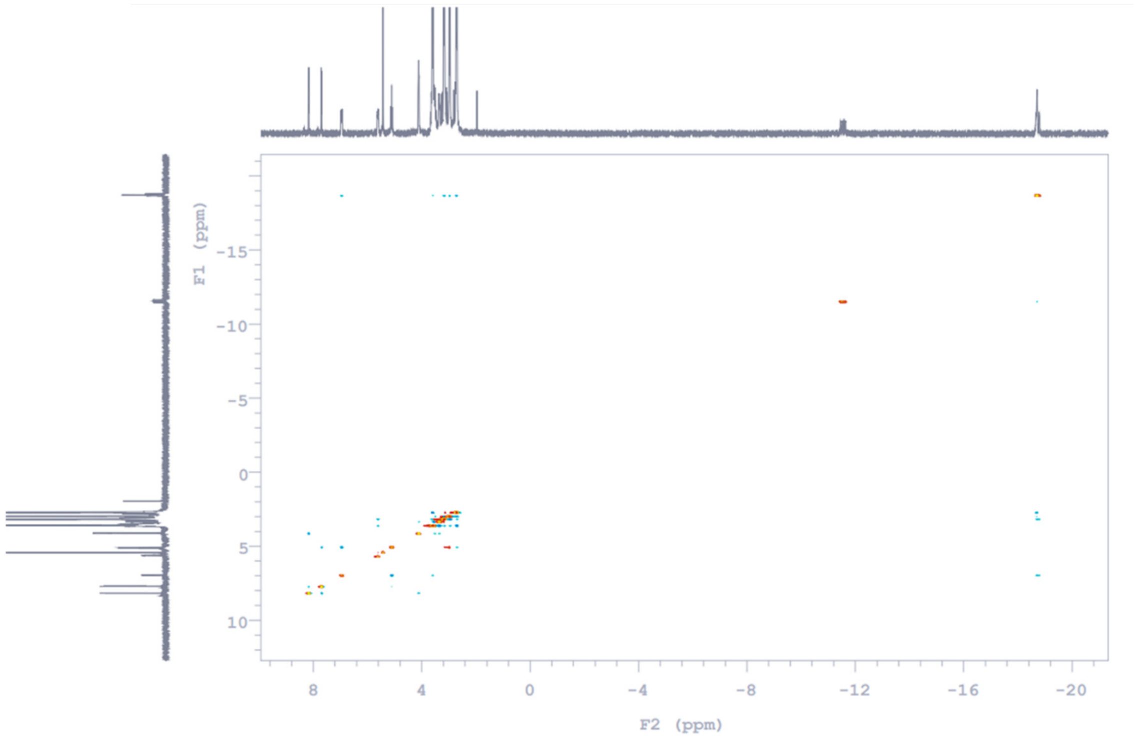

Figure S48: The Hadamard encoded NOESY NMR spectrum of complex **5** in THF-d_8 at 25 °C.

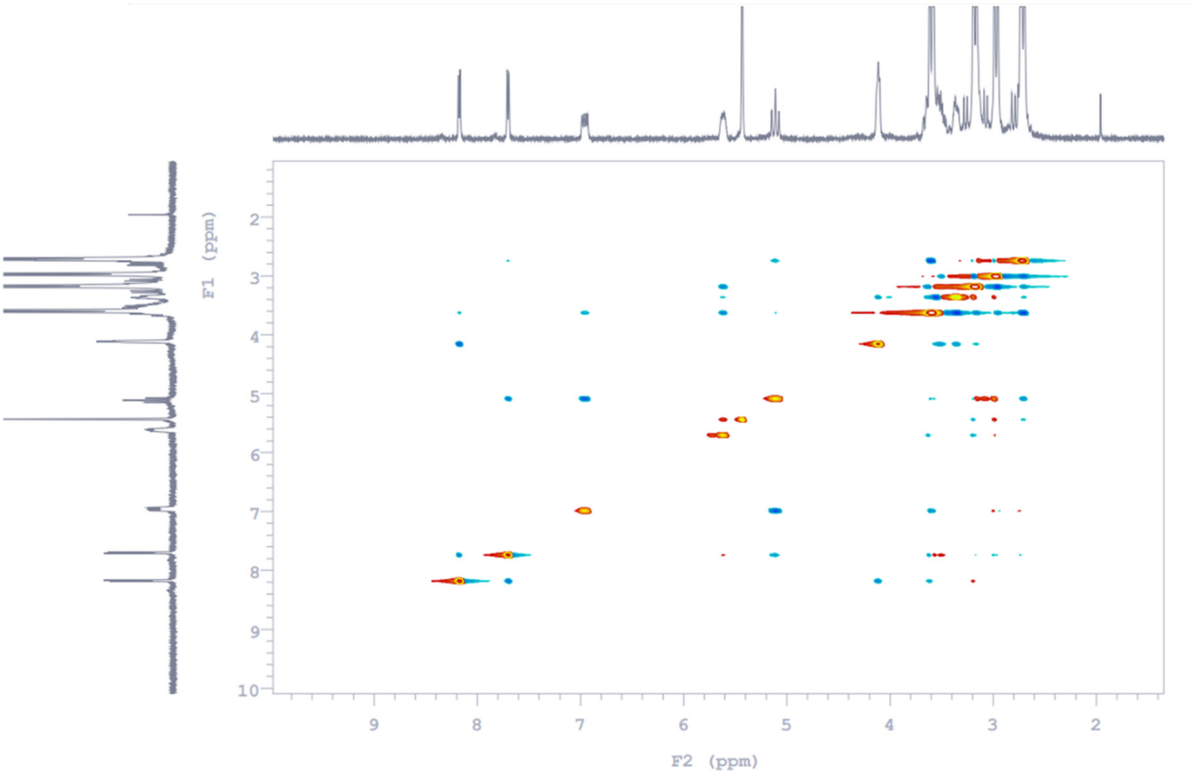

Figure S49: A zoom in of the Hadamard encoded NOESY NMR spectrum of complex **5** in THF-d_8 at 25 °C.

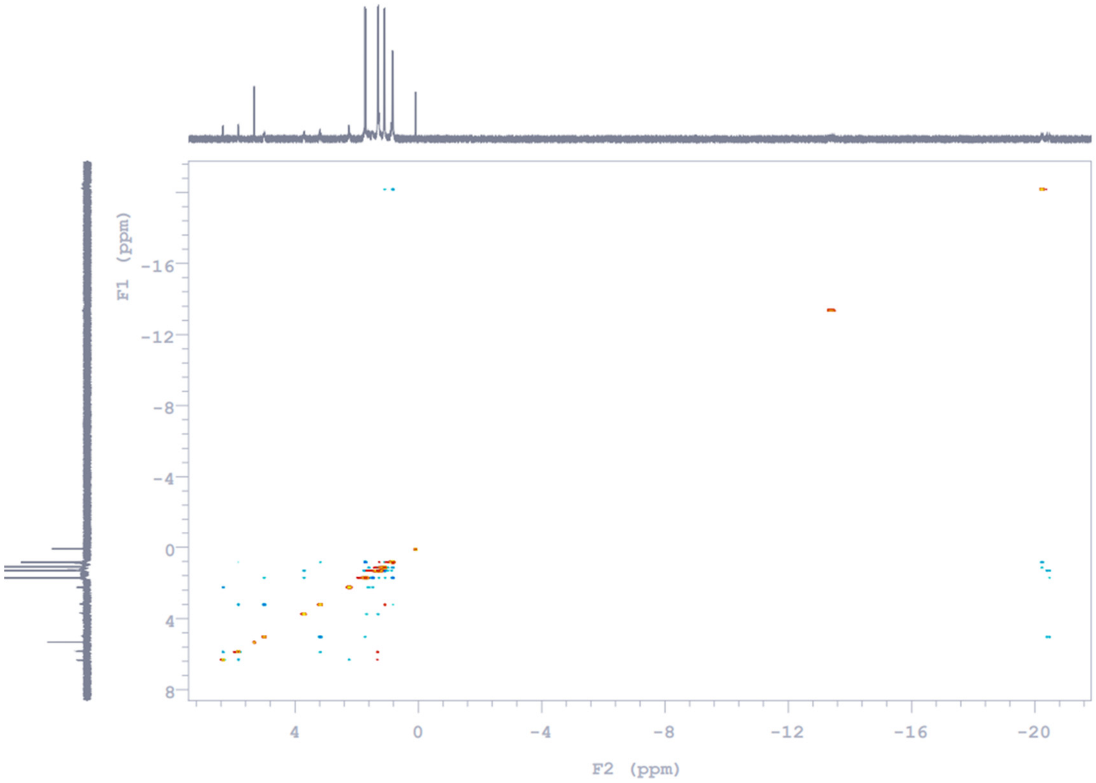

Figure S50: The Hadamard encoded NOESY NMR spectrum of complex **5** in DCM-d_2 at 25 °C.

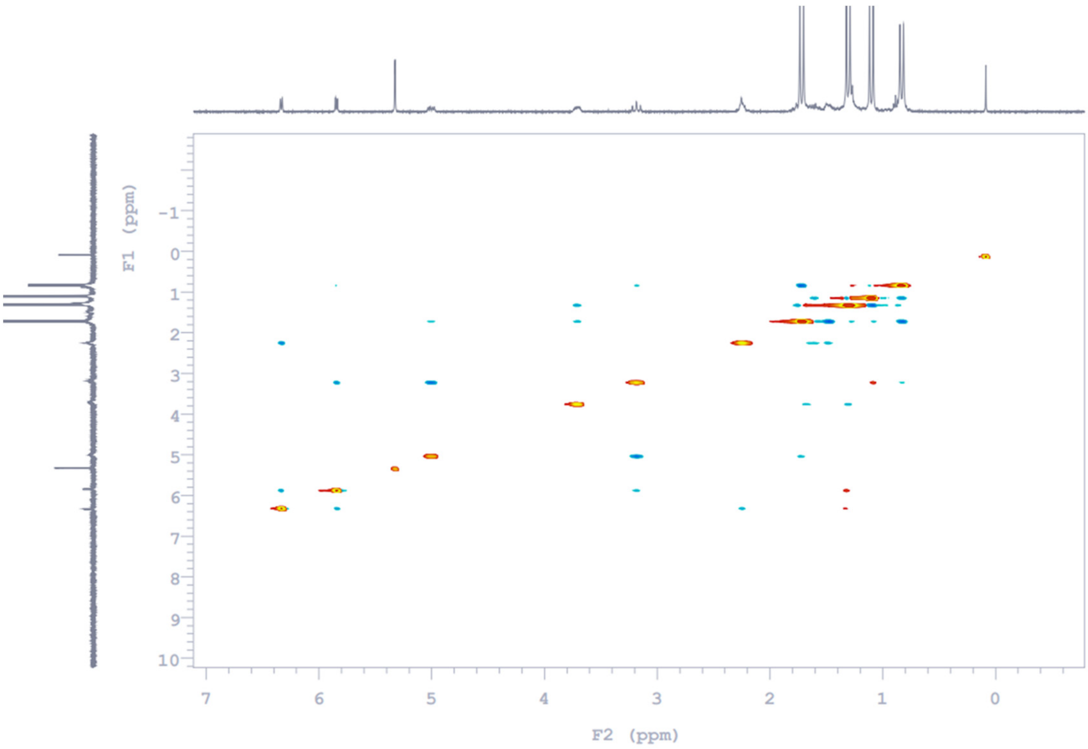

Figure S51: A zoom in of the Hadamard encoded NOESY NMR spectrum of complex **5** in DCM-d_2 at 25 °C.

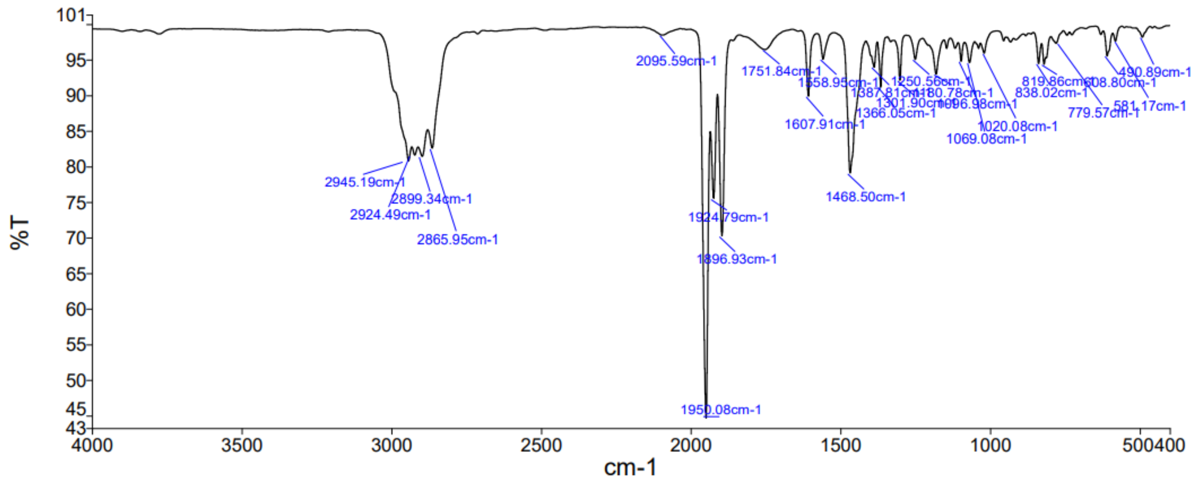

Figure S52: The ATR-IR spectrum of compound **5**, measured as a film under N_2 flow.

Assignment of spectroscopic characterization

X-ray absorption spectroscopy of complex 1

Experimental

The Ru K-edge X-ray absorption spectrum of **1** was measured at the Stanford Synchrotron Radiation Lightsource (SSRL) on the unfocused 20-pole 2T wiggler side-station beam line 7-3 under standard ring conditions of 3 GeV and ~500 mA. A Si(220) double crystal monochromator was used for energy selection. The sample was stored and handled in a dry N_2-filled glovebox. The sample was prepared by grinding 19 mg of the compound with 11 mg cellulose in an agate mortar and pestle to form a uniformly colored, fine powder. The powder was pressed into a 7 mm diameter cylindrical pellet and held in 64 µm Kapton tape. During data collection, the sample was held in a Cryo Industries closed cycle liquid He cryostat at a temperature of 10 K. Spectra were measured to k = 17 Å^{-1} in transmission mode using N_2-filled ionization chambers with simultaneous measurement of a 400 mesh metallic Ru powder for energy calibration. The second inflection point of the metallic Ru standard was fixed at 22117 eV.

Initial data processing was done in the Athena program of the Demeter package.[6] Data presented here were obtained by aligning and merging three replicate scans. A three-region spline of orders 2, 3, 3 was used to model the post-edge background function using the Pyspline program.[7] EXAFS was modeled using the Artemis program of the Demeter package.[6] Theoretical EXAFS signals χ(k) were calculated using FEFF6. Absorber-backscatter scattering paths were generated from the atomic coordinates derived from the DFT-optimized structure. Improvement of the input structure was not required since the model provided accurate theoretical EXAFS signals. The EXAFS model was optimized in *k*-space using k^1, k^2, and k^3 weightings, with the model obeying the Nyquist criterion.[8] Fitting was performed on a *k*-range of 2-17 Å^{-1} and an R-range of 1-3 Å. The structural parameters varied during the fitting process were the bond distance (R) and the bond variance (σ^2), which is related to the Debye-Waller factor resulting from thermal motion and static disorder of the absorbing and scattering atoms. The non-structural parameter, ΔE_0 (E_0 = the energy at which *k* equals 0), was also allowed to vary. Coordination numbers were systematically varied over the course of fitting to assess different models but were fixed during a given fit.

XANES and EXAFS analysis of complex 1

Due to the lack of structural characterization of complex **1**, it was further characterized by X-ray absorption near edge structure (XANES) and extended X-ray absorption fine structure (EXAFS) analysis to obtain more insights into the bonding within complex **1**. The EXAFS plot and fit in *k*-space with k^3 weighting are shown in Figure 2 in the main text. The corresponding table of the EXAFS fitting results is shown in Table S1. These results support the DFT-optimized structure (BP86/def2-TZVP) of complex **1**, see Figure S53.

Table S1. Ru K-edge EXAFS fit parameters for **1**.

Path	Degeneracy	R_{fit} (Å)	R_{DFT} (Å)[c]	σ^2 (Å^2)[a]	ΔE_0 (eV)	R factor
Ru-C	1	1.82 ± 0.02	1.83	150 ± 173	-4.7 ± 6.0	0.18
Ru-N	1	2.16 ± 0.07	2.18	273 ± 140[b]		
Ru-P	1	2.28 ± 0.03	2.25	273 ± 140[b]		
Ru-Cl	1.5	2.48 ± 0.02	2.44	234 ± 137		
Ru-Ru	1	2.81 ± 0.02	2.79	176 ± 58		
Ru-C-O	2	2.99 ± 0.03	2.99	206 ± 245		

[a]The σ^2 values are multiplied by 10^5

[b]Parameters were constrained to be equal

[c] These values were obtained from the DFT-optimized structure of complex **1**

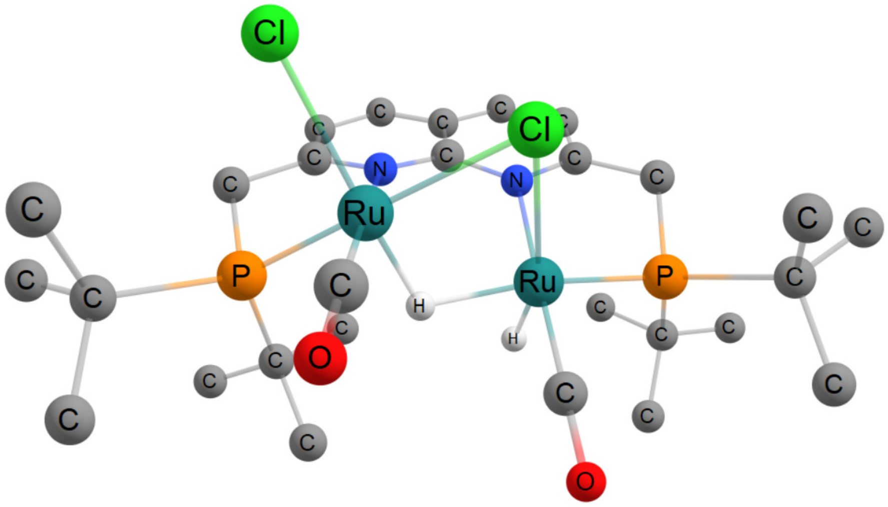

Figure S53: The DFT-optimized structure (BP86/def2-TZVP) of complex **1**.

The ssNMR spectra of complex **1** showed the presence of a second minor species to be present in complex **1**. As described in the main text, the possibility of four different isomers with differing arrangement of the (terminal or bridging) hydride and chloride ligands was evaluated. The relative stability of these isomers were computationally assessed using DFT by a geometry optimization of the isomers (BP86/def2-TZVP level) and are shown in Figure S54. The geometry of the structure of complex **1** and isomers 1 and 2 converged to an optimized geometry. However, the structure of isomer 3 would not converge to an optimized geometry, despite several attempts, suggesting that this is not a stable isomer. The structure of complex **1** was found to be the lowest in energy. Isomer 1 is found at an energy 8.8 kcal·mol^{-1} higher than that of complex **1**. Isomer 2 is found at a lower energy that isomer 1 and is 7.9 kcal·mol^{-1} higher in energy than complex **1**. These computational results indicate that the proposed structure of complex **1** indeed has the arrangement of the hydride and chloride ligands in its lowest energy. Combined with the ssNMR, XAS and IR data we propose that the major product obtained when ***^{tBu}*PNNP** is reacted with two equivalents of $RuHCl(PPh_3)_3(CO)$ has the structure of the proposed structure of complex **1** and the minor product is 2 as drawn in Figure S54.

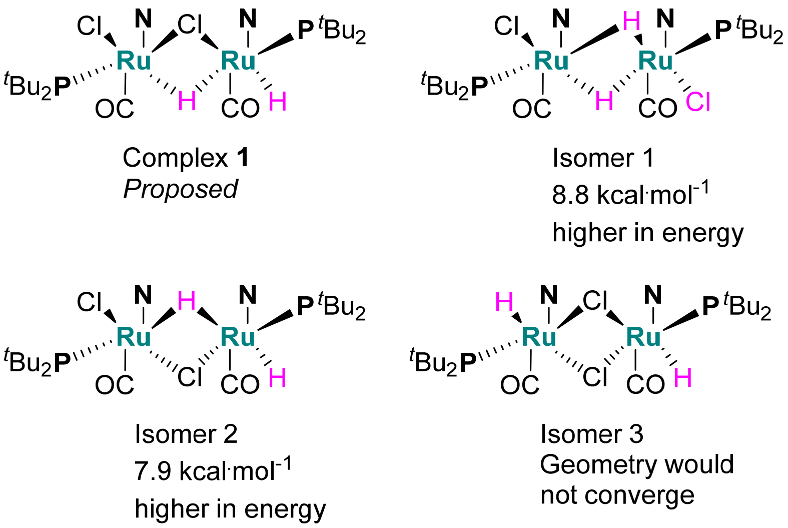

Figure S54: The simplified proposed structure of complex **1** and its three proposed different isomers with only the donor atoms of the ***^{tBu}*PNNP** ligand shown.

Table S2. Ru K-edge EXAFS fit parameters for **1** and averaged bond lengths in geometry optimized isomers.

**Path**	**Degeneracy**	**R_{fit} (Å)**	**$R_{Complex\ 1}$ (Å)^c**	**$R_{isomer\ 1}$ (Å)**	**$R_{isomer\ 2}$(Å)**	**$R_{isomer\ 3}$(Å)**
Ru–C	1	1.82 ± 0.02	1.83	1.83	1.82	NA
Ru–N	1	2.16 ± 0.07	2.18	2.18	2.19	NA
Ru–P	1	2.28 ± 0.03	2.25	2.24	2.27	NA
Ru–Cl	1.5	2.48 ± 0.02	2.44	2.45	2.42	NA
Ru–Ru	1	2.81 ± 0.02	2.79	2.68	2.84	NA
Ru–C-O	2	2.99 ± 0.03	2.99	2.99	2.99	NA

ATR-IR comparison between complex 1 and 2

The proposed structure of complex **1** is similar to the structure of complex **2**. Therefore, a similarity for their ATR-IR spectra is expected. An overlay of the ATR-IR spectra of complex **1** and **2** is shown in Figure S55. Indeed, both spectra display two terminal carbonyl bands at similar energies, although the carbonyl bands of complex **1** are redshifted compared to those of complex **2**. Similarly, both spectra show weak bands around ν = 2100 cm^{-1} that are proposed terminal Ru–H bands. These results show that complex **1** has a structure comparable to that of complex **2**.

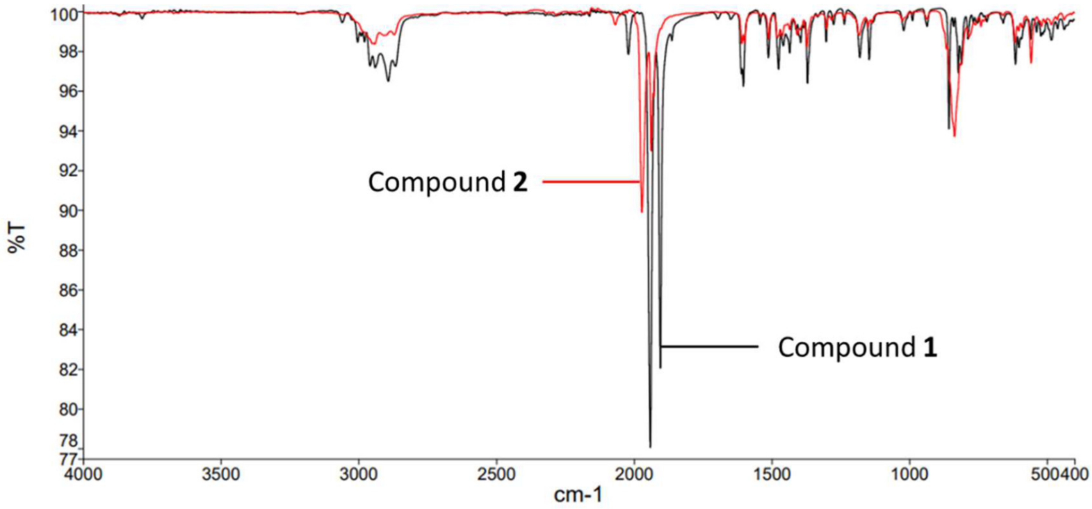

Figure S55: An overlay of the ATR-IR spectra of complex **1** (black) and complex **2** (red).

2D NMR characterization

In the structure of complex **2**, the hydride ligands were not located in the difference-Fourier maps, but were placed in the structure in calculated positions. Therefore, 2D NMR techniques were employed to identify the positioning of these ligands. In the ^{1}H NMR spectrum of complex **2** in THF-d_8 at 25 °C, a doublet (δ = -14.52 ppm) and a doublet of doublets (δ = -15.80 ppm) were observed, corresponding to the two hydrides. In the ^{1}H{^{31}P} NMR spectrum these couplings are no longer present, suggesting the couplings originate only from coupling with phosphorus nuclei. The exact origin of the multiplicity of the hydride signals was investigated using ^{1}H-^{31}P HMBC NMR spectroscopy, which probes ^{1}H-^{31}P coupling over multiple bonds. The ^{1}H-^{31}P HMBC spectrum optimized for a multiple bond $J_{H,P}$-coupling of 8 Hz shows coupling of both hydride signals to the ^{31}P signal at δ = 107.1 ppm (Figure S56, left). When the multiple bond $J_{H,P}$-coupling is optimized for 12 Hz (Figure S56, right), the ^{1}H-^{31}P HMBC spectrum shows coupling of the hydride signal at δ = -15.80 ppm to the ^{31}P signal at δ = 109.4 ppm. These observations are consistent with the presence of a terminal bound hydride (δ = -14.52 ppm) that only couples to one of the phosphorus nuclei with $^2J_{H,P}$ = 31.0 Hz and a bridging hydride (δ = -15.80 ppm) that couples to both phosphorus nuclei with $^2J_{H,P}$ = 14.6 and $^2J_{H,P}$ = 8.1 Hz.

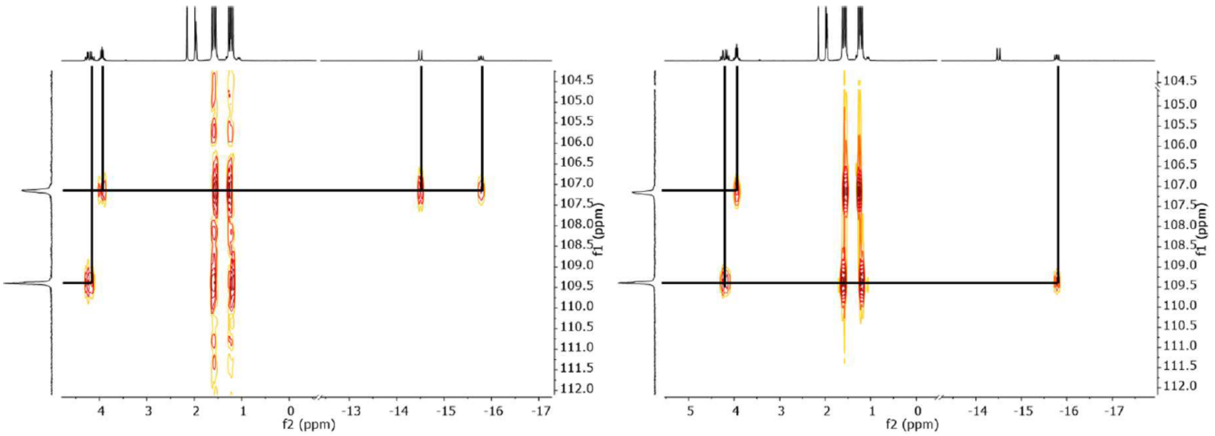

Figure S56: The ^1H-^{31}P HMBC spectrum of complex **2** in THF-d_8 at 25 °C optimized for a multiple bond $J_{H,P}$-coupling of 8 Hz (left) and 12 Hz (right).

Additional evidence for the proposed positioning of the hydrides in complex **2** was obtained with Hadamard 2D-NOESY NMR.[9] The NOESY spectrum shows NOE between both hydride signals (Figure S57). This would be expected, as the distance between the two hydrides in the crystal structure is 2.37 Å, well within the typical distance in which NOE signals are usually observed. Furthermore, the opposite phase of these signals with respect to the signals on the diagonal suggests no exchange between the hydrides.

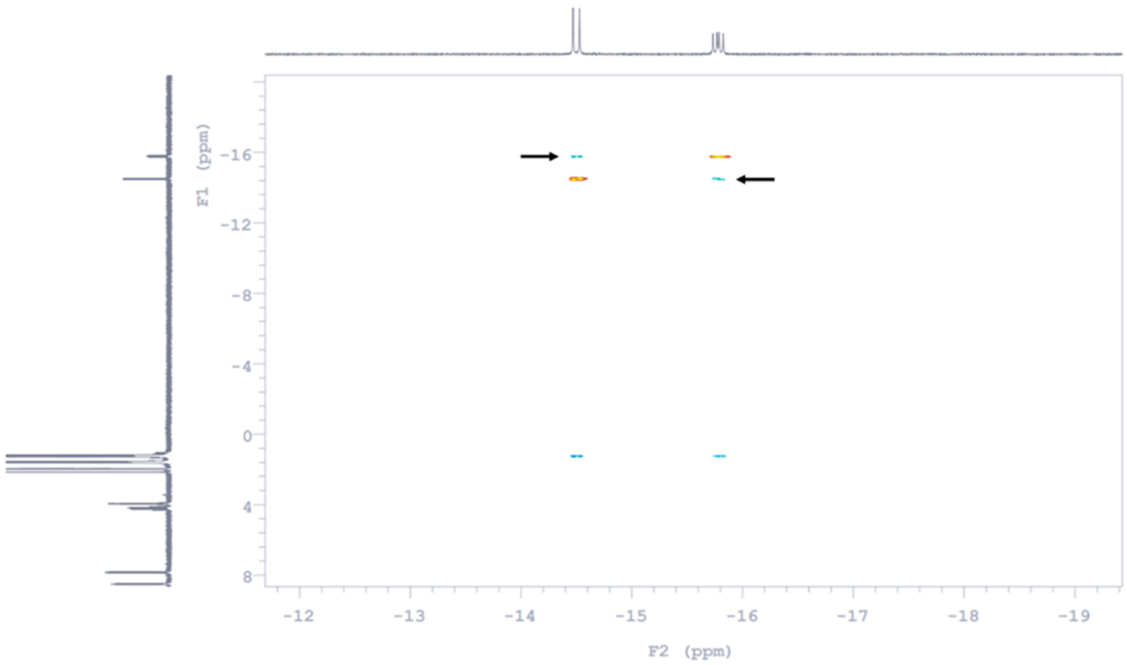

Figure S57: A zoom-in of the Hadamard encoded 2D-NOESY spectrum of complex **2** in THF-d_8 at 25 °C, showing NOE between the two hydrides. The relevant cross-peaks are marked with arrows.

The hydride signals in complex **3** appear as a doublet and a doublet of doublets, like in complex **2**. This indicates that complex **3** contains a bridging and a terminal hydride ligand too. Too confirm this, and to establish which PN pocket is dearomatized in the structure of complex **3**, several 2D NMR

experiments were performed. The ^{1}H-^{31}P HMBC NMR spectrum optimized for a multiple bond $J_{H,P}$-coupling of 8 Hz shows coupling of one phosphorus nucleus (δ = 104.1 ppm) to both hydrides, as well as coupling of the other phosphorus nucleus (δ = 91.4 ppm) to the methine proton. Changing the optimal multiple bond $J_{H,P}$-coupling to 12 Hz reveals coupling of the ^{31}P signal at δ = 91.4 ppm to the hydride signal at δ = -20.97 ppm (Figure 58). Similarly to what was discussed for complex **2**, these results indeed confirm the presence of a bridging and a terminal hydride, as well as show that the ligand is deprotonated on the opposite side from the terminally bound hydride. A Hadamard 2D NOESY experiment showed NOE between both hydride signals, which additionally had an opposite phase to the signals on the diagonal, suggesting proximity and no exchange between the hydrides (Figure 59).

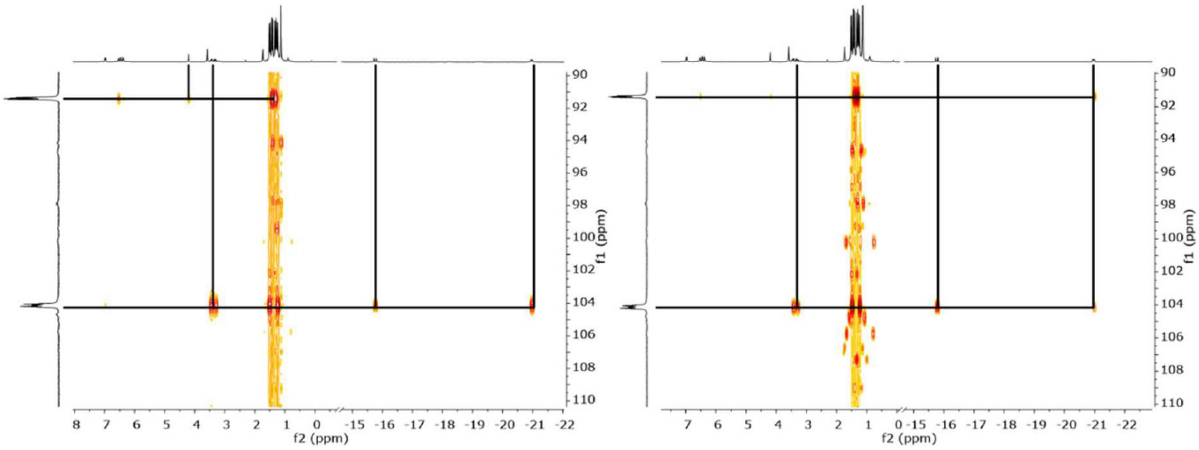

Figure S58: The ^{1}H-^{31}P HMBC spectrum of complex **3** in THF-d_8 at 25 °C optimized for a multiple bond $J_{H,P}$-coupling of 8 Hz (left) and 12 Hz (right).

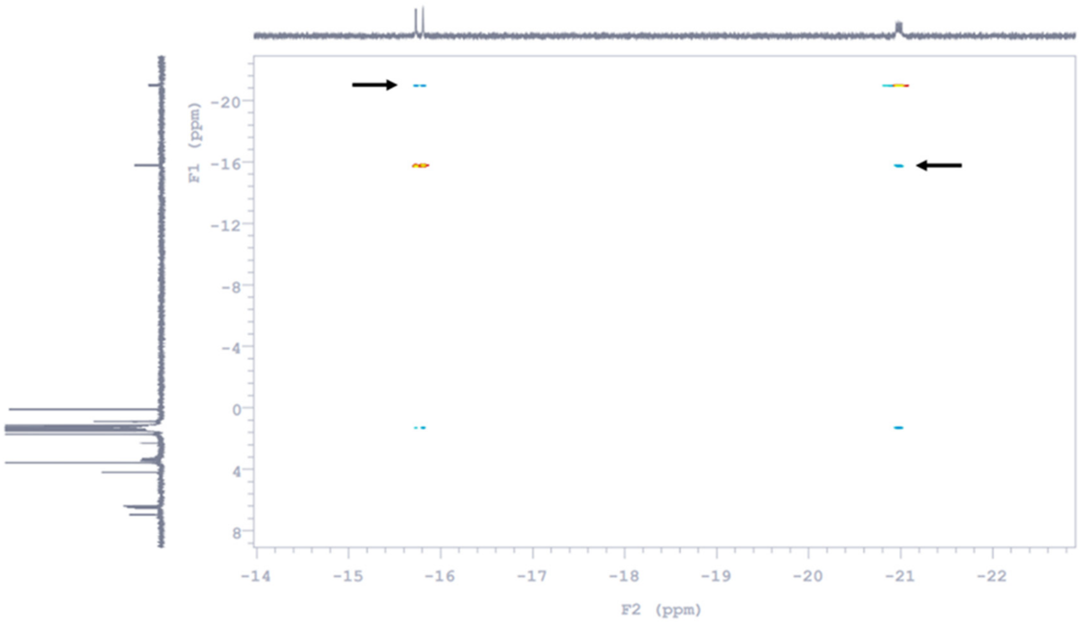

Figure S59: A zoom-in of the Hadamard encoded 2D-NOESY spectrum of complex **3** in THF-d_8 at 25 °C, showing NOE between the two hydrides. The relevant cross-peaks are marked with arrows.

The hydride ligands of complex **5** were not located in the difference-Fourier maps, but the hydrides were placed at calculated positions. To further support the assignment and placement of these hydrides in solution, 2D NMR experiments were performed. In the ^{1}H NMR spectrum of complex **5** in

CD_2Cl_2 at 25 °C, three hydride resonances are present at δ = -13.41, -20.25 and -20.46 ppm. The signal at δ = -13.41 ppm appears as a doublet of doublets; the other two hydride signals show more complex coupling patterns. In the $^{1}H\{^{31P}\}$ NMR spectrum, the signal at δ = -13.41 ppm appears as a singlet. The other two hydride signals retain some of their couplings in the $^{1}H\{^{31}P\}$ NMR spectrum that are likely caused by mutual coupling between the hydrides.

The NOESY NMR spectrum allows for assigning these hydride signals, as well as determining whether the complex is a dimer in solution. A zoom-in of the NOESY NMR spectrum is given in Figure S60. The NOESY NMR spectrum shows NOE between the hydride signal at δ = -20.46 ppm and a signal at δ = 5.00 ppm. This latter signal belongs to one of the methylene protons on the aromatic side of the ligand, as established by other NMR experiments. Also, a weak NOE is present between this hydride and a signal at δ = 3.70 ppm, belonging to the -C*H* proton on the hydrogenated side of the ligand. The simultaneous proximity of one of the hydrides to both these protons implies that the dimer is intact in solution, as visually explained in Figure S60. Another argument for the persistence of the dimer in solution is the large difference in chemical shift between the geminal protons on the methylene linker on the aromatic side of the ligand. These appear at δ = 5.00 ppm and 3.18 ppm. This large difference implies widely different chemical environments, which could be caused by dimerization. For a monomer, geminal protons with a chemical shift difference within a few tenths of ppm would be expected, as is the case for complex **2** and **3**.

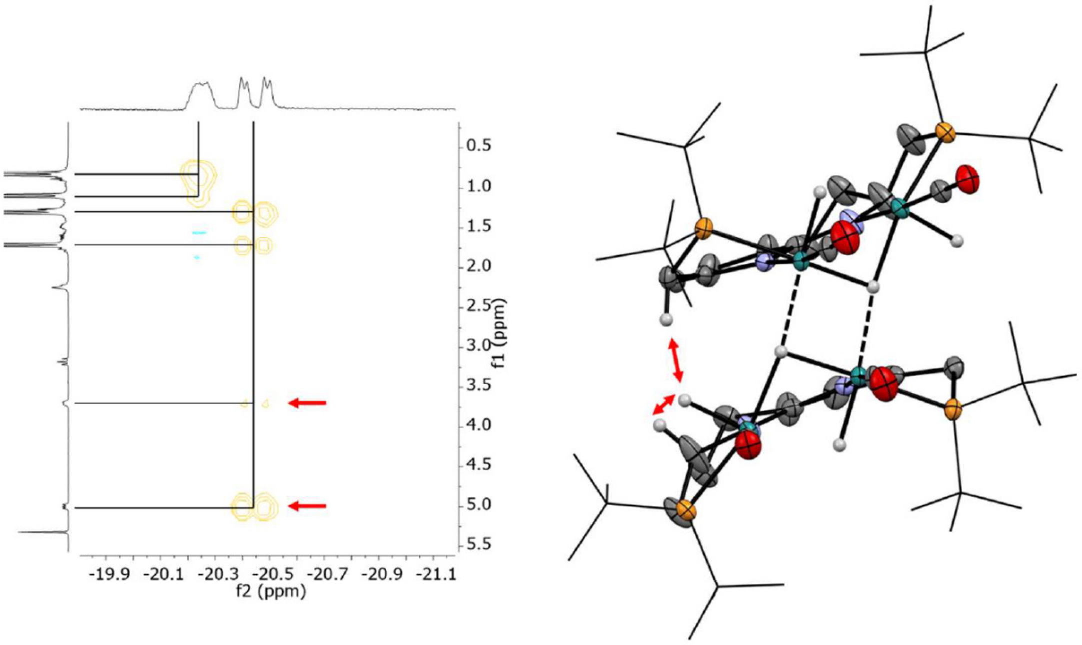

Figure S60: A zoom-in of the NOESY NMR spectrum in CD_2Cl_2 at 25 °C of complex **5** (left) and the displacement ellipsoid plot (50 % probability) of complex **5** from the X-ray crystal structure. Red arrows show relevant NOE signals (right, *tert*-butyl groups are shown as wireframe, most hydrogen atoms are omitted. Only the major disorder component is shown). Positions of the Ru–H hydrogen atoms in the crystal structure are only tentative.

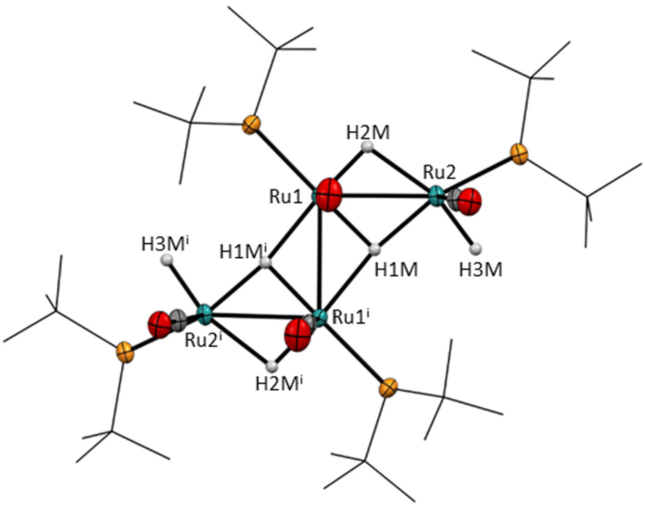

Figure S61: Selected atoms in the displacement ellipsoid plot (50% probability) of complex **5** in the crystal (*tert*-butyl groups are shown as wireframe). Positions of the Ru–H hydrogen atoms in the crystal structure are only tentative. Symmetry code i: 1-x, 1-y, z.

The above is consistent with the signal at δ = - 20.46 ppm corresponding to H3M in the crystal structure (Figure S61), and the signal at δ = -20.25 ppm corresponding to H2M. This is further corroborated by the NOE between the hydrides and the various tert-butyl groups, where both these hydrides are in proximity to two of the tert-butyl groups, but not to the same tert-butyl groups. This also implies the presence of a dimer in solution, since H3M is in proximity to *tert*-butyl groups above and below the naphthyridine plane of the monomer. The doublet of doublets at δ = -13.41 ppm corresponds to H1M, and coupling to both ^{31}P nuclei is present, as is the case for the bridging hydride in complex **2** and **3**.

X-ray crystal structure determinations

X-ray crystal structure determination of complex 1

$[C_{30}H_{49}ClN_3O_2P_2Ru_2](PF_6)\cdot 2C_6H_6$, Fw = 1084.43, orange needle, 0.21 × 0.07 × 0.03 mm^3, triclinic, $P\overline{1}$ (no. 2), a = 12.1901(9), b = 15.1398(13), c = 15.3312(16) Å; α = 115.117(6), β = 103.800(3), γ = 98.937(5) °, V = 2379.7(4) $Å^3$, Z = 2, D_x = 1.513 g/cm^3, μ = 0.85 mm^{-1}. The diffraction experiment was performed on a Bruker Kappa ApexII diffractometer with sealed tube and Triumph monochromator (λ = 0.71073 Å) at a temperature of 150(2) K up to a resolution of $(\sin \theta/\lambda)_{max}$ = 0.61 $Å^{-1}$. The Eval15 software[10] was used for the intensity integration. A numerical absorption correction and scaling was performed with SADABS[11] (correction range 0.72-1.00). A total of 24792 reflections was measured, 8857 reflections were unique (R_{int} = 0.091), 3953 reflections were observed [I>2σ(I)]. The structure was solved with Patterson superposition methods using SHELXT.[12] Structure refinement was performed with SHELXL-2018[13] on F^2 of all reflections. Non-hydrogen atoms were refined freely with anisotropic displacement parameters. One of the co-crystallized benzene molecules was refined with a disorder model. In this weakly diffracting crystal, all hydrogen atoms were introduced in calculated positions. The Ru–H hydrogen atoms were kept fixed on their calculated positions, the C–H hydrogen atoms were refined with a riding model. 600 Parameters were refined with 639 restraints (distances, angles and displacement parameters for PF_6 anion and benzene solvent molecules). R1/wR2 [I > 2σ(I)]: 0.0689 / 0.1555. R1/wR2 [all refl.]: 0.1809 / 0.2076. S = 0.940. Residual electron density between -2.12 and 1.15 e/$Å^3$. Geometry calculations and checking for higher symmetry was performed with the PLATON program.[14]

X-ray crystal structure determination of complex 5

$C_{56}H_{96}N_4O_4P_4Ru_4$, Fw = 1417.52, black block, 0.41 × 0.24 × 0.20 mm^3, tetragonal, $I4_1cd$ (no. 110), a = b = 23.3566(4), c = 22.6554(7) Å, V = 12359.2(6) $Å^3$, Z = 8, D_x = 1.524 g/cm^3, μ = 1.11 mm^{-1}. The diffraction experiment was performed on a Bruker Kappa ApexII diffractometer with sealed tube and Triumph monochromator (λ = 0.71073 Å) at a temperature of 150(2) K up to a resolution of $(\sin \theta/\lambda)_{max}$ = 0.65 $Å^{-1}$. The Eval15 software[10] was used for the intensity integration. The crystal was fragmented in several pieces. Only the non-overlapping reflections of the major fragment were used for structure solution and refinement. A multi-scan absorption correction and scaling was performed with SADABS[11] (correction range 0.37-0.43). A total of 108945 reflections was measured, 7106 reflections were unique (R_{int} = 0.034), 6891 reflections were observed [I>2σ(I)]. The structure was solved with Patterson superposition methods using SHELXT.[12] Structure refinement was performed with SHELXL-2018[13] on F^2 of all reflections. Non-hydrogen atoms were refined freely with anisotropic displacement parameters. The "pincer arm" at C10A/C10B and two *t*-butyl groups were refined with disorder models. The Ru–H hydrogen atoms were tentatively placed on difference-Fourier peaks and kept fixed. The C–H hydrogen atoms were introduced in calculated positions and refined with a riding model. 443 Parameters were refined with 714 restraints (distances, angles and displacement parameters for the disordered moieties). R1/wR2 [I > 2σ(I)]: 0.0174 / 0.0433. R1/wR2 [all refl.]: 0.0183 / 0.0436. S = 1.042. Flack parameter[15] x = -0.018(8). Residual electron density between -0.44 and 1.09 e/$Å^3$. Geometry calculations and checking for higher symmetry was performed with the PLATON program.[14]

CCDC 2160732-2160733 contain the supplementary crystallographic data for this paper. These data can be obtained free of charge from The Cambridge Crystallographic Data Centre via http://www.ccdc.cam.ac.uk/data_request/cif.

Table S3: Selected bond distances (Å) for complex **2** and **5**. In compound **5**, the moiety involving atoms C8, C9 and C10 is disordered. Only the major disorder form is considered, here. Symmetry code i: 1-x, 1-y, z.

Bond	[(^{tBu}PNNP)$Ru_2H_2Cl(CO)_2$MeCN][PF_6] (**2**)	Bond	[(^{tBu}THPNNP)$Ru_2H_3(CO)_2]_2$ (**5**)
Ru11–Ru21	2.8149(10)	Ru1-Ru2	2.7364(3)
Ru11–Cl1	2.482(3)	Ru1-Ru1^{i}	2.8627(4)
Ru11–P11	2.277(4)	Ru1-P1	2.3016(8)
Ru11–N11	2.169(6)	Ru1-N1	2.165(3)
Ru11–N31	2.101(7)	Ru1-C27	1.828(4)
Ru11–C271	1.844(9)	Ru2-P2	2.2466(8)
Ru21–Cl1	2.528(2)	Ru2-N2	2.149(3)
Ru21–P21	2.275(3)	Ru2-C28	1.833(3)
Ru21–N21	2.214(6)	P1-C1	1.839(4)
Ru21-C281	1.807(9)	P2-C10A	1.782(7)
P11-C11	1.827(9)	N1-C2	1.366(4)
P21–C101	1.821(8)	N1-C6	1.369(4)
N11-C21	1.364(12)	N2-C6	1.355(4)
N11-C61	1.370(10)	N2-C9A	1.426(7)
N21-C61	1.394(11)	C1-C2	1.500(5)
N21-C91	1.335(10)	C9A-C10A	1.414(9)
C11-C21	1.498(12)	C2-C3	1.382(5)
C9–C10	1.500(12)	C3-C4	1.375(6)
C21–C31	1.417(12)	C4-C5	1.376(5)
C31–C41	1.370(13)	C5-C6	1.432(4)
C41–C51	1.423(13)	C5-C7	1.448(5)
C51–C61	1.411(10)	C7-C8A	1.325(8)
C51–C71	1.385(12)	C8A-C9A	1.528(9)
C71–C81	1.355(12)		
C81–C91	1.401(10)		

Monitoring the diphenylacetylene hydrogenation by ^1H NMR spectroscopy

The catalytic activity of Milstein's monometallic 16 VE count [Ru(^{tBu}PNP*)H(CO)] complex[1] as a catalyst for the semi-hydrogenation of diphenylacetylene was investigated. The kinetic plot of the semi-hydrogenation of diphenylacetylene using Ru(^{tBu}PNP*)H(CO)] (5 mol%) in C_6D_6 solution under a H_2 atmosphere (1 atm) is shown in Figure S62. The major product in this reaction is 1,2-diphenylethane.

An *in situ* prepared C_6D_6 stock solution of [Ru(^{tBu}PNP*)H(CO)] (2.6 µmol/0.60 mL C_6D_6) was made by treating [Ru(^{tBu}PNP)HCl(CO)] with an equimolar amount of KO*t*Bu. 0.60 mL of this stock solution was added to diphenylacetylene (9.2 mg, 51.7 µmol) and mesitylene (3.0 µL, 2.59 mg, 21.5 µmol) was added as internal standard. The sample was transferred to a J Young valved NMR tube and degassed by three freeze-pump-thaw cycles and filled with H_2 (1 atm), after which the sample was monitored by ^1H NMR spectroscopy.

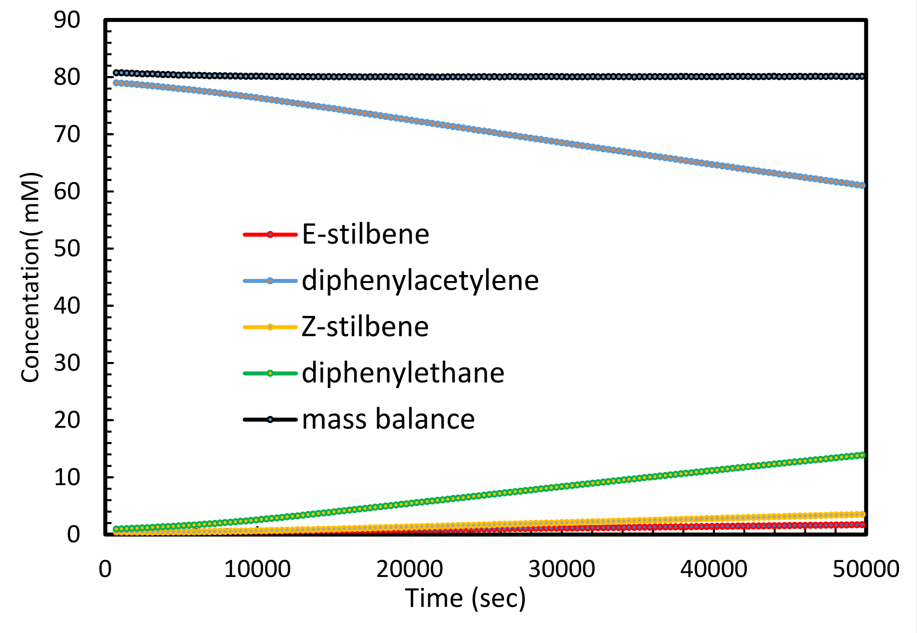

Figure S62: Kinetic plot of the hydrogenation of diphenylacetylene catalyzed by [Ru(^{tBu}PNP*)H(CO)] (5 mol%) showing the concentration of the various compounds based on integrals of the ^1H NMR resonances versus time with mesitylene as internal standard (relaxation time set at 20 sec) at 25 °C. The first data point was collected after 740 sec after addition of H_2.

Isomerization catalysis

Since the kinetic profile in Figure 3 in the main text suggests that hydrogenation of diphenylacetylene is followed by *Z*-to-*E* isomerization, the catalytic activity of **3** and **5** towards the isomerization of *Z*-stilbene to *E*-stilbene was studied. C_6D_6 solutions (2.5 µmol complex/0.60 mL C_6D_6) of either complex **3** or complex **5** were treated with *Z*-stilbene (8.9 µL, 9.0 mg, 50 µmol) and mesitylene (internal standard, 3 µL) inside a N_2-filled glovebox, and quickly transferred to an NMR spectrometer. The reaction mixtures were analyzed 10 min after mixing by ^{1}H NMR spectroscopy, which showed full conversion of *Z*-stilbene to *E*-stilbene. This shows that catalytic isomerization is rapid under these conditions. In similar experiments, stock solutions (0.60 mL) of complex **3** or **5** (2.5 µmol complex/0.60 mL C_6D_6, 5 mol%) and mesitylene (internal standard, 3 µL) inside J Young valved NMR tubes were mixed with *Z*-stilbene (8.9 µL, 9.0 mg, 50 µmol), mixed and immediately frozen in liquid N_2. These samples were degassed by removing the headspace under vacuum and backfilled with H_2 (1 atm). Upon thawing these samples, the mixtures were analyzed by ^{1}H NMR spectroscopy, showing that rapid isomerization of *Z*-stilbene to *E*-stilbene was observed, indicating that the isomerization of *Z*-stilbene to *E*-stilbene is not affected by the changing a N_2 atmosphere for a H_2 atmosphere.

Subsequently, the NMR tube that was filled with *Z*-stilbene and complex **3** was degassed by three freeze-pump-thaw cycles and filled with H_2 (1 atm) for 16 h at ambient temperature. Minor conversion of *E*-stilbene formed by isomerization of *Z*-stilbene to diphenylethane is observed (~5%).

Additionally, mixtures containing equimolar amounts of diphenylacetylene and *Z*-stilbene and either complex **3** or **5** (5 mol%) were prepared under N_2 atmosphere. In these samples, no formation of *E*-stilbene was observed indicating that the *Z*-to-*E* isomerization was inhibited by the alkyne substrate under these conditions.

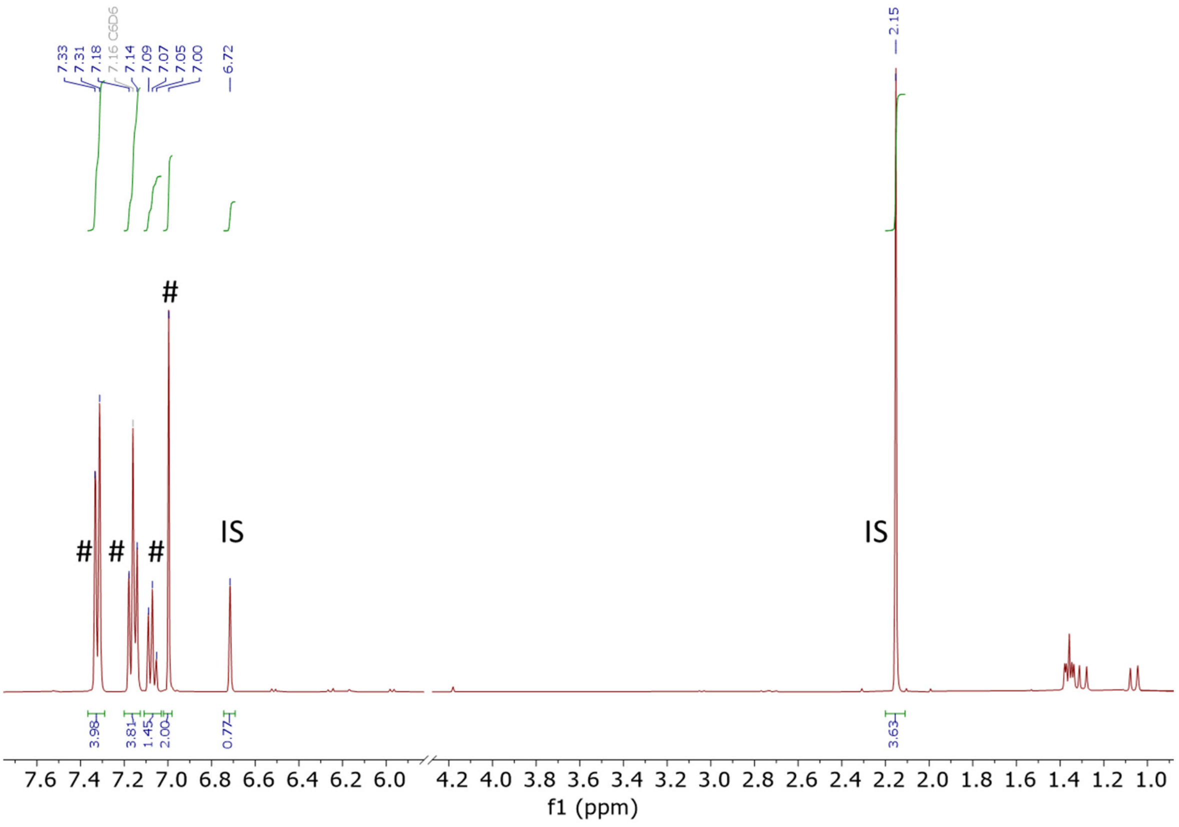

Figure S63: The ^{1}H NMR spectrum measured in C_6D_6 at 25 °C after 10 minutes of mixing *Z*-stilbene with complex **3** (5 mol%) and mesitylene as internal standard (IS) in N_2 atmosphere. Full conversion to *E*-stilbene (#) is observed.

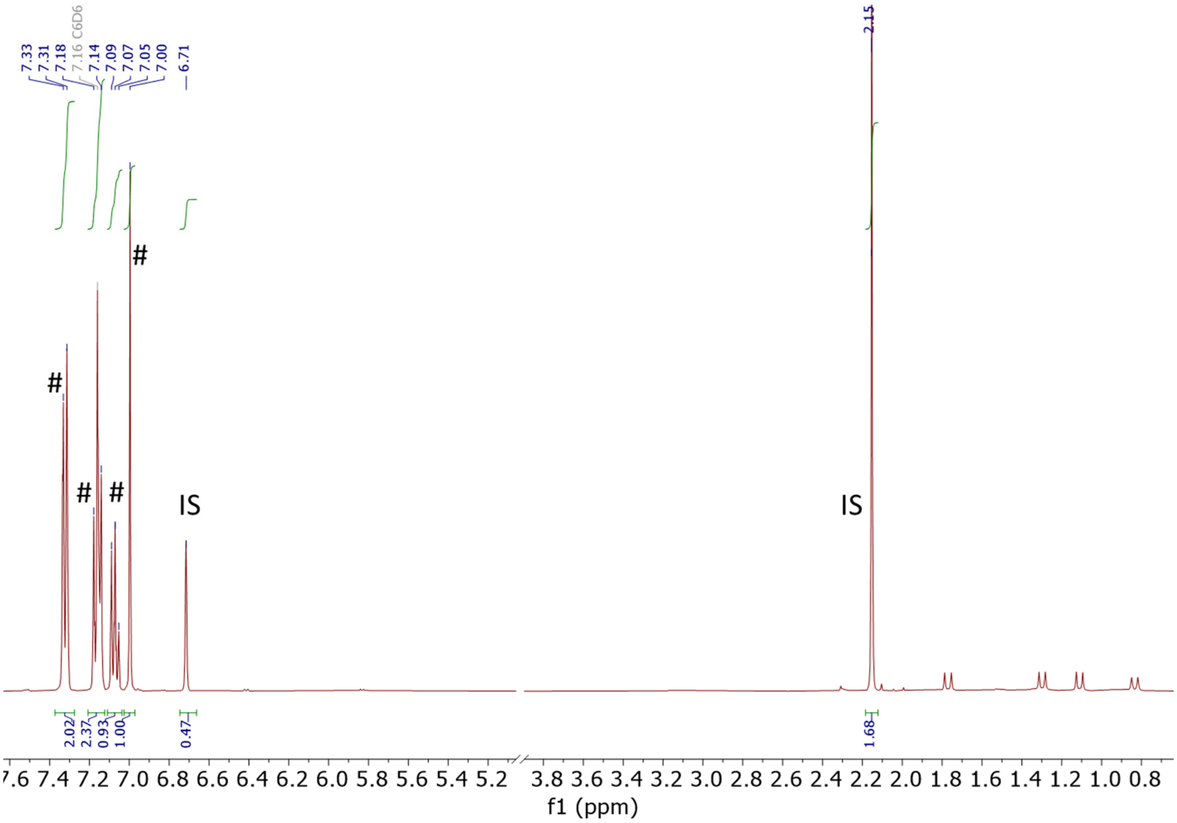

Figure S64: The ^1H NMR spectrum measured in C_6D_6 at 25 °C after 10 minutes of mixing *Z*-stilbene with complex **5** (5 mol%) and mesitylene as internal standard (IS) in N_2 atmosphere. Full conversion to *E*-stilbene (#) is observed.

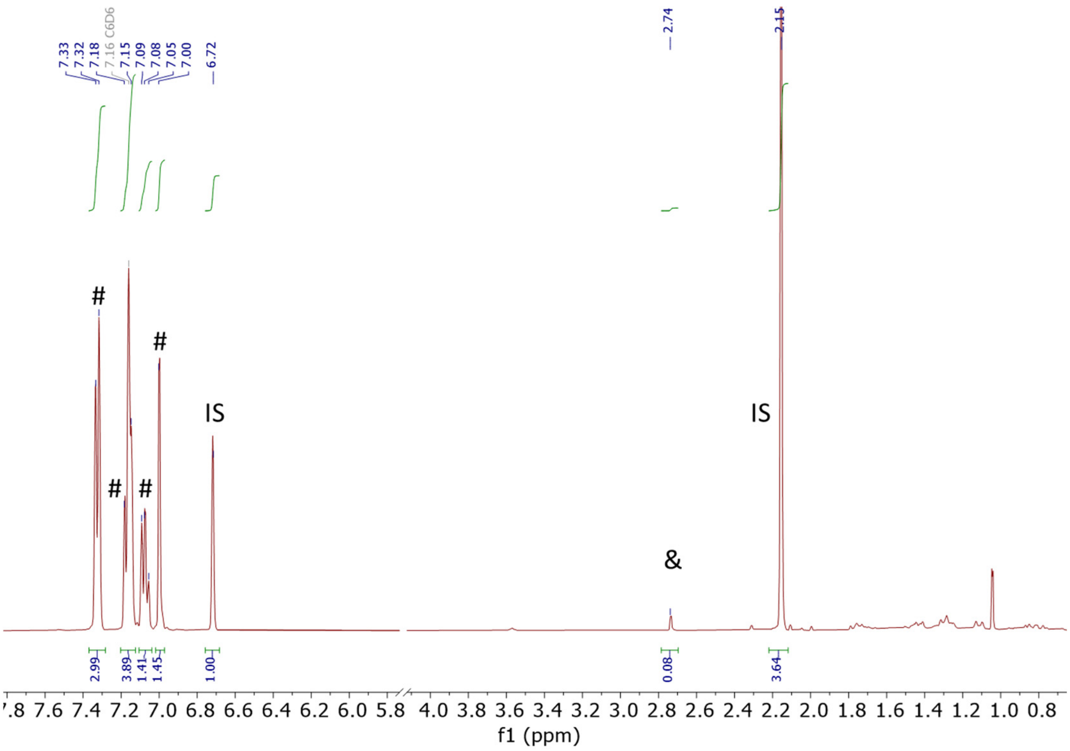

Figure S65: The ^1H NMR spectrum measured in C_6D_6 at 25 °C after exposing a sample filled with *Z*-stilbene and complex **3** (5 mol%) and mesitylene as internal standard (IS) in a H_2 (1 atm) atmosphere for 16 h at ambient temperature. Minor conversion of *E*-stilbene (#) formed by isomerization of *Z*-stilbene to diphenylethane (&) is observed (~5%).

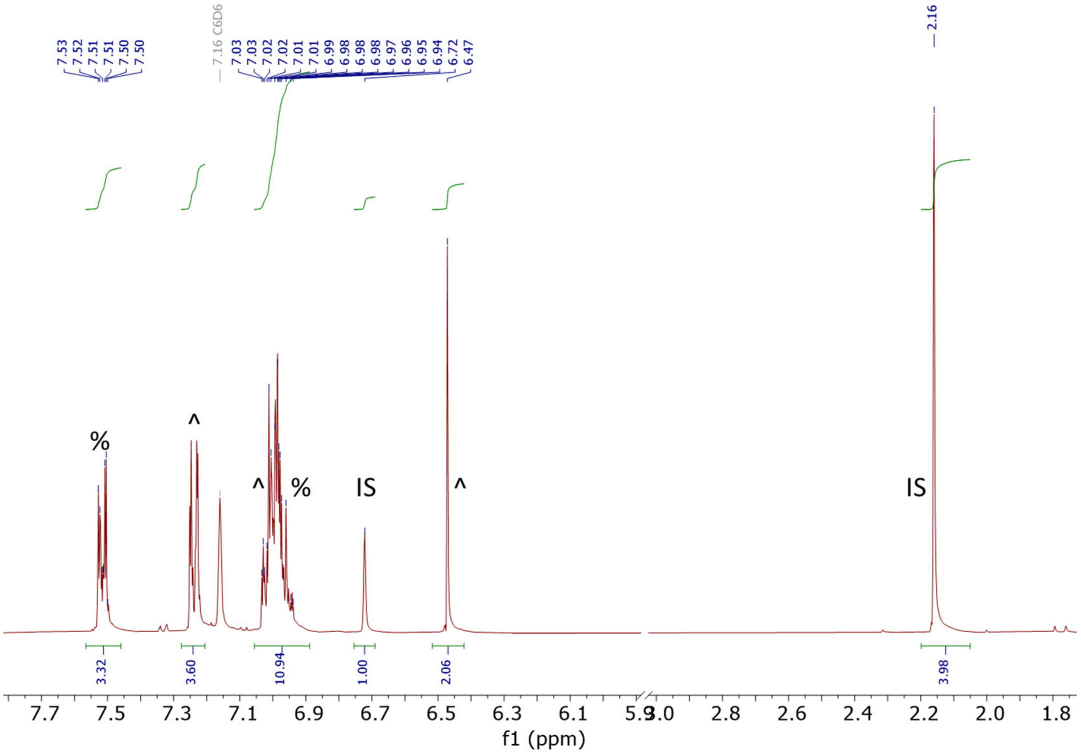

Figure S66: The ^{1}H NMR spectrum measured in C_6D_6 at 25 °C after mixing a sample filled with equimolar *Z*-stilbene (^) and diphenylacetylene (%) and complex **5** (5 mol%) and mesitylene as internal standard (IS) in a N_2 atmosphere after 18 h at ambient temperature. No traces of *E*-stilbene were observed, formed by isomerization of *Z*-stilbene.

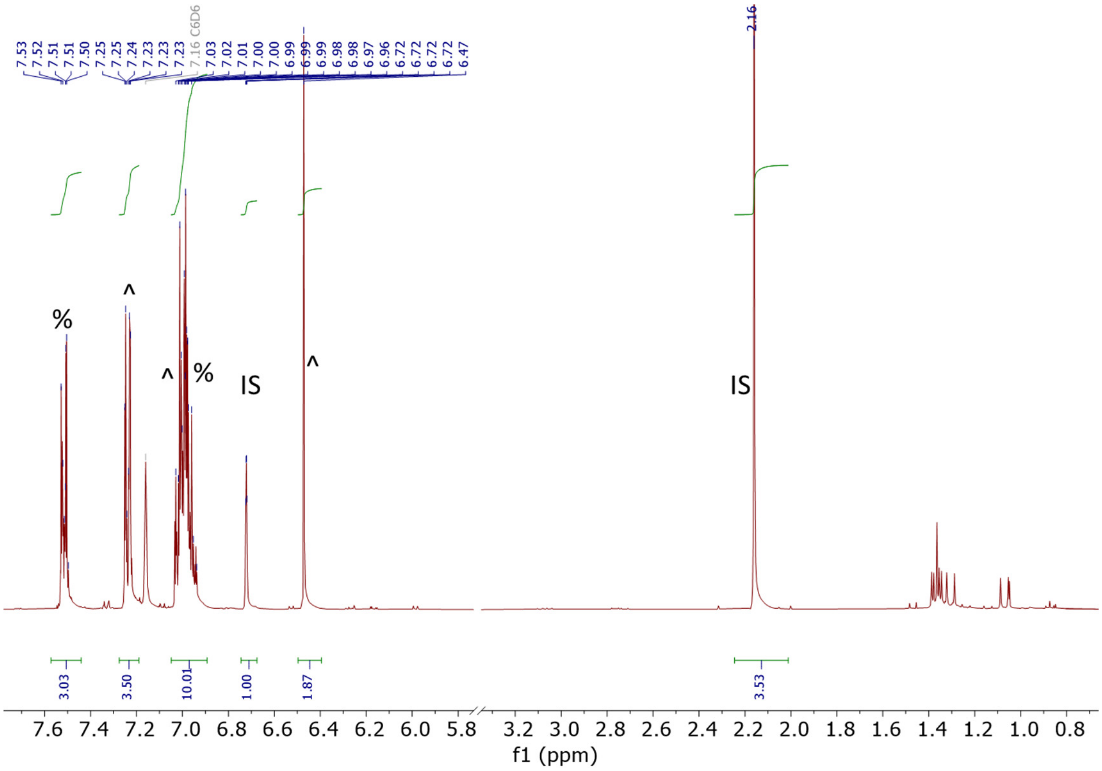

Figure S67: The ^{1}H NMR spectrum measured at 25 °C in C_6D_6 after mixing a sample filled with equimolar *Z*-stilbene (^) and diphenylacetylene (%) and complex **3** (5 mol%) and mesitylene as internal standard (IS) in a N_2 atmosphere after 5.5 h at ambient temperature. No traces of *E*-stilbene were observed, formed by isomerization of *Z*-stilbene.

Semi-hydrogenation of diphenylacetylenes

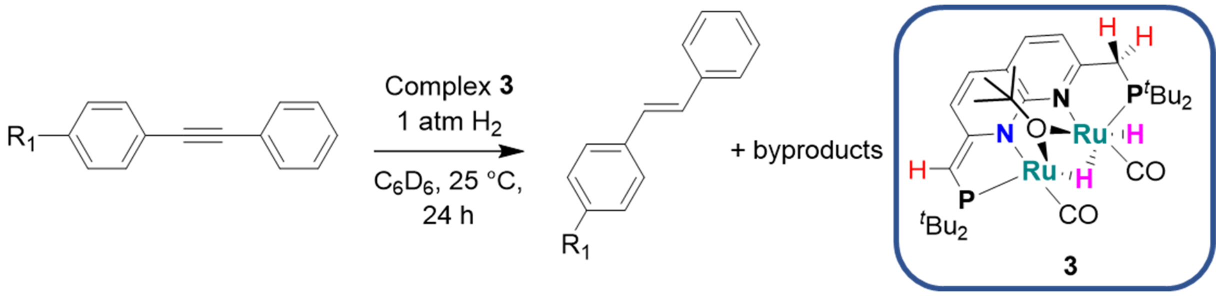

Figure S68: Semi-hydrogenation of diphenylacetylene derivatives in C_6D_6 using hydrogen (1 atm) at 25 °C using complex **3**.

Having identified that complex **3** is a catalyst for the semi-hydrogenation of diphenylacetylene under ambient H_2 pressure and temperature, we investigated the functional group tolerance of *E*-selective semi-hydrogenation of diphenylacetylene derivatives. For these experiments, a stock solution of complex **3** (2.5 µmol/0.60 mL) in C_6D_6 was prepared. 0.60 mL of this stock solution was added to the alkyne substrate (50 µmol, 20 equivalents). The solutions were then transferred to J Young valved NMR tubes and mesitylene (internal standard, 3.0 µL) was added. The mixtures were degassed by three freeze-pump-thaw cycles and filled with H_2 (1 atm). The mixtures were placed in an oil bath at 25 °C (or 40 °C for some experiments) and the quantitative NMR spectra were collected after 24 h or 48 h (using an acquisition time of 5 sec and a relaxation time between scans set at 20 sec). Spectroscopic yields were determined with mesitylene as the internal standard. Assignment of the products is based on chemical shift, (the ratio of) integrals and comparison with reported NMR spectra in the literature. For some of the starting materials and products the ^{1}H NMR spectra have not been reported in C_6D_6. In those cases, identification of the products was achieved by evaporation of the reaction mixture and redissolving the mixture in $CDCl_3$. For most of the compounds, reference spectra in $CDCl_3$ have been reported. *Z*- and *E*-alkenes of the diphenylacetylene derivatives can be distinguished using the *J*-coupling constants[16] of the alkene resonances and their chemical shifts. *E*-alkenes have larger *J*-coupling constants between the alkene protons than *Z*-alkenes. Furthermore, *E*-alkene protons are found more downfield than *Z*-alkene protons.

Diphenylacetylene

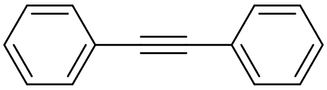

The ^{1}H NMR spectra of the semi-hydrogenation of diphenylacetylene before and after H_2 addition are shown in Figures S69-S70. Full conversion of diphenylacetylene is observed and *E*-stilbene and 1,2-diphenylethane are identified. The ^{1}H NMR resonances match those reported in literature.[17]

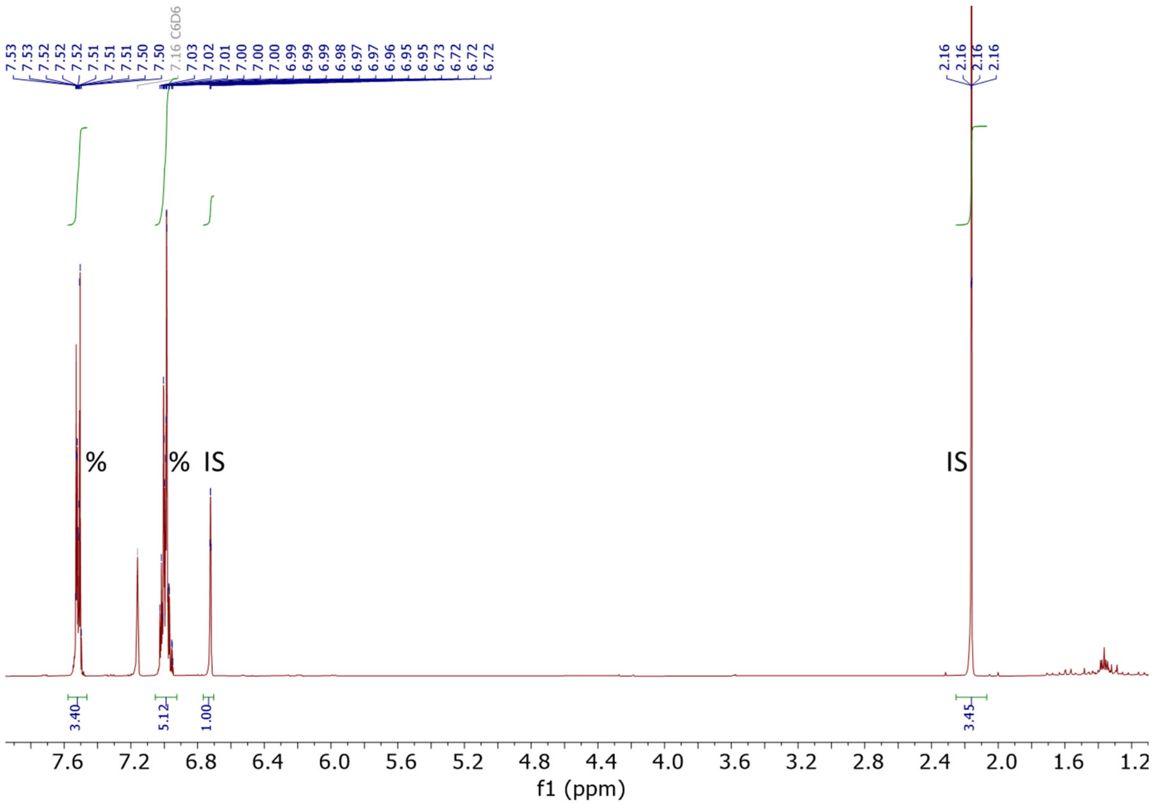

Figure S69: The ^{1}H NMR spectrum measured at 25 °C in C_6D_6 of the semi-hydrogenation diphenylacetylene (%) with mesitylene as internal standard (IS) before the addition of a H_2 atmosphere.

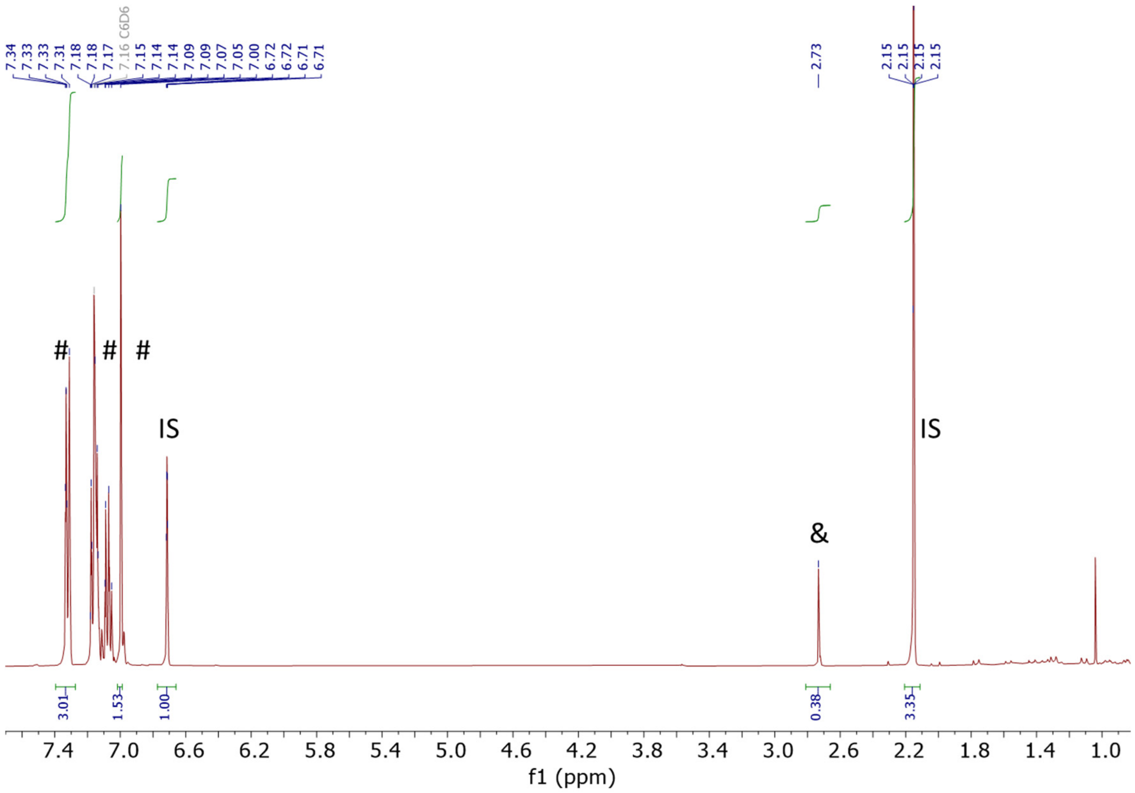

Figure S70: The ^{1}H NMR spectrum measured at 25 °C in C_6D_6 of the semi-hydrogenation diphenylacetylene (%) with mesitylene as internal standard (IS) after 24 h at 25 °C. The corresponding *E*-alkene is assigned by # and the corresponding alkane is assigned by &.

Methyl 4-(phenylethynyl)benzoate

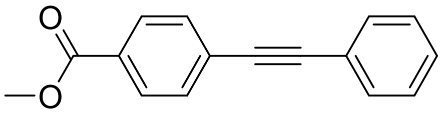

The ^{1}H NMR spectra of the semi-hydrogenation of methyl 4-(phenylethynyl)benzoate before and after H_2 addition are shown in Figures S71-S72. Full conversion of the starting material is observed after 24 h. Its corresponding *E*-alkene and alkane products are identified. The products of this reaction have not been reported in C_6D_6. However, the *J*-coupling constants observed for the alkene protons are consistent of that of an *E*-alkene (^3J = 16.4 Hz). To confirm, the reaction mixture was analyzed additionally in $CDCl_3$ and its spectrum matches reported spectra for the *E*-alkene product[18] and the alkane product.[19]

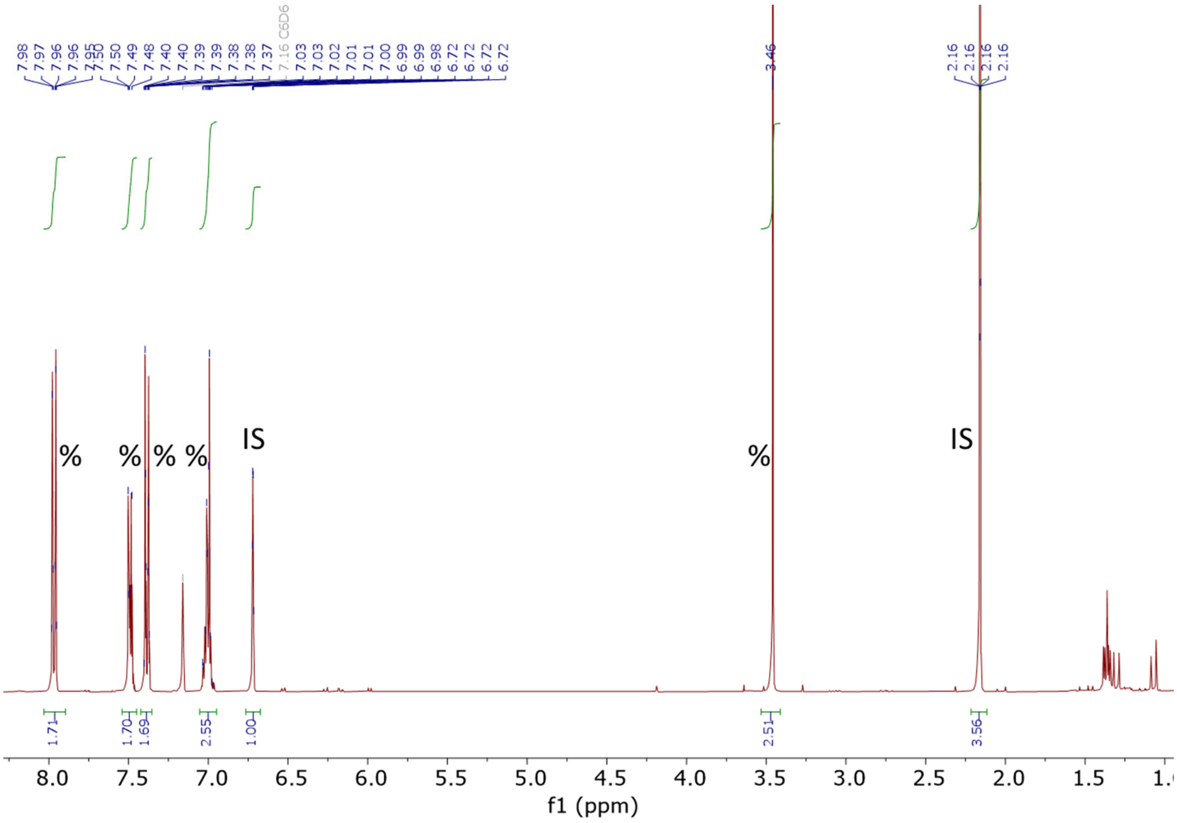

Figure S71: The ^{1}H NMR spectrum measured at 25 °C in C_6D_6 of the semi-hydrogenation of methyl 4-(phenylethynyl)benzoate (%) with mesitylene as internal standard (IS) before the addition of a H_2 atmosphere.

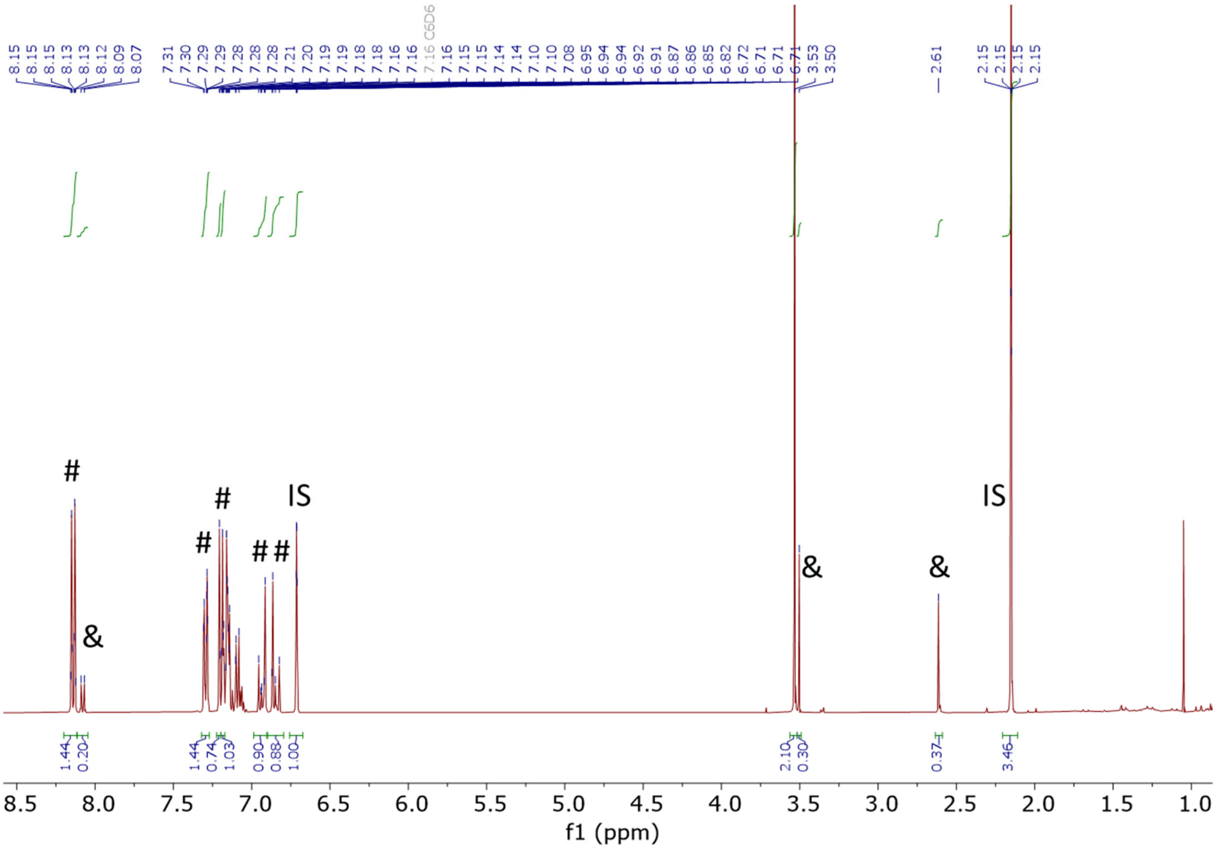

Figure S72: The ^{1}H NMR spectrum measured at 25 °C in C_6D_6 of the semi-hydrogenation of methyl 4-(phenylethynyl)benzoate (%) with mesitylene as internal standard (IS) after 24 h at 25 °C. The corresponding *E*-alkene is assigned by # and the corresponding alkane is assigned by &.

4-(2-phenylethynyl)benzonitrile

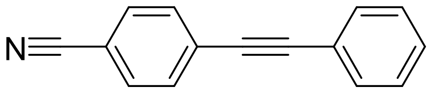

The ^{1}H NMR spectra of the semi-hydrogenation of 4-(2-phenylethynyl)benzonitrile before and after H_2 addition are shown in Figures S73-S75. The substrate is not fully consumed after 24 or 48 h at 25 °C. After 24 h, the starting material, the *Z*-alkene and alkane product could be identified. Performing the semi-hydrogenation at 40 °C for 24 h results in full conversion of the starting material and the *E*-alkene and alkane product could be identified. The *J*-coupling constant of ^3J = 12.2 Hz corresponds to the *Z*-alkane product that is formed and the *J*-coupling constant of ^3J = 16.5 Hz corresponds to the *E*-alkene. The spectra match reported spectra for the *Z*-alkene product,[20] the *E*-alkene product.[18] An ^{1}H NMR spectra of a $CDCl_3$ solution of the reaction mixture containing the alkane product matches that of reported spectra of the alkane product.[21]

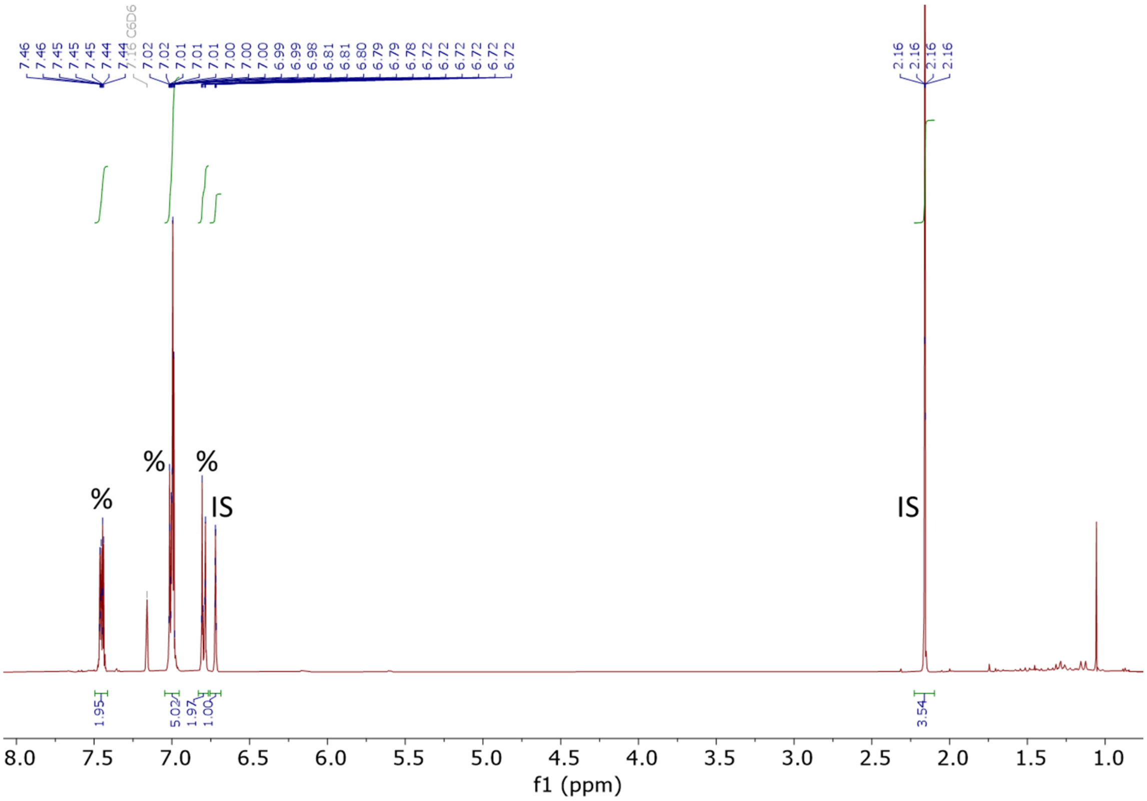

Figure S73: The ^{1}H NMR spectrum measured at 25 °C in C_6D_6 of the semi-hydrogenation of 4-(2-phenylethynyl)benzonitrile (%) with mesitylene as internal standard (IS) before the addition of a H_2 atmosphere.

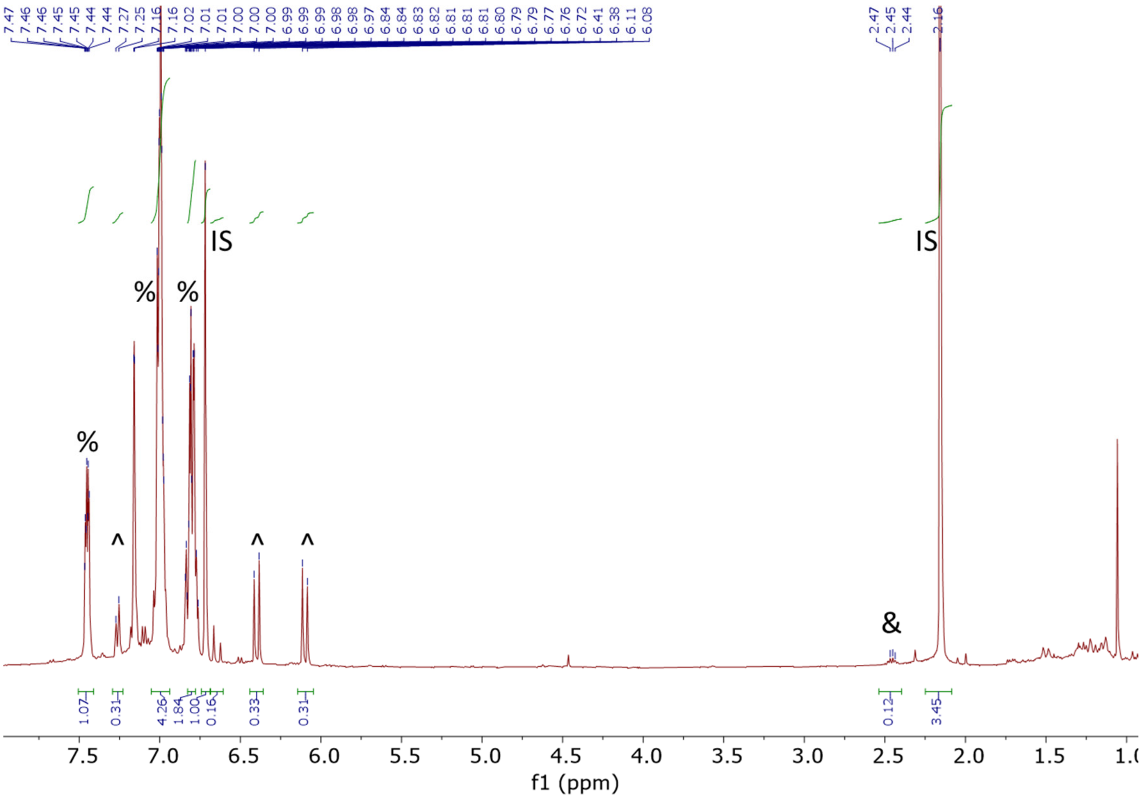

Figure S74: The ^{1}H NMR spectrum measured at 25 °C in C_6D_6 of the semi-hydrogenation of 4-(2-phenylethynyl)benzonitrile (%) with mesitylene as internal standard (IS) after 24 h at 25 °C. The corresponding *Z*-alkene is assigned by ^, the corresponding *E*-alkene is assigned by # and the corresponding alkane is assigned by &.

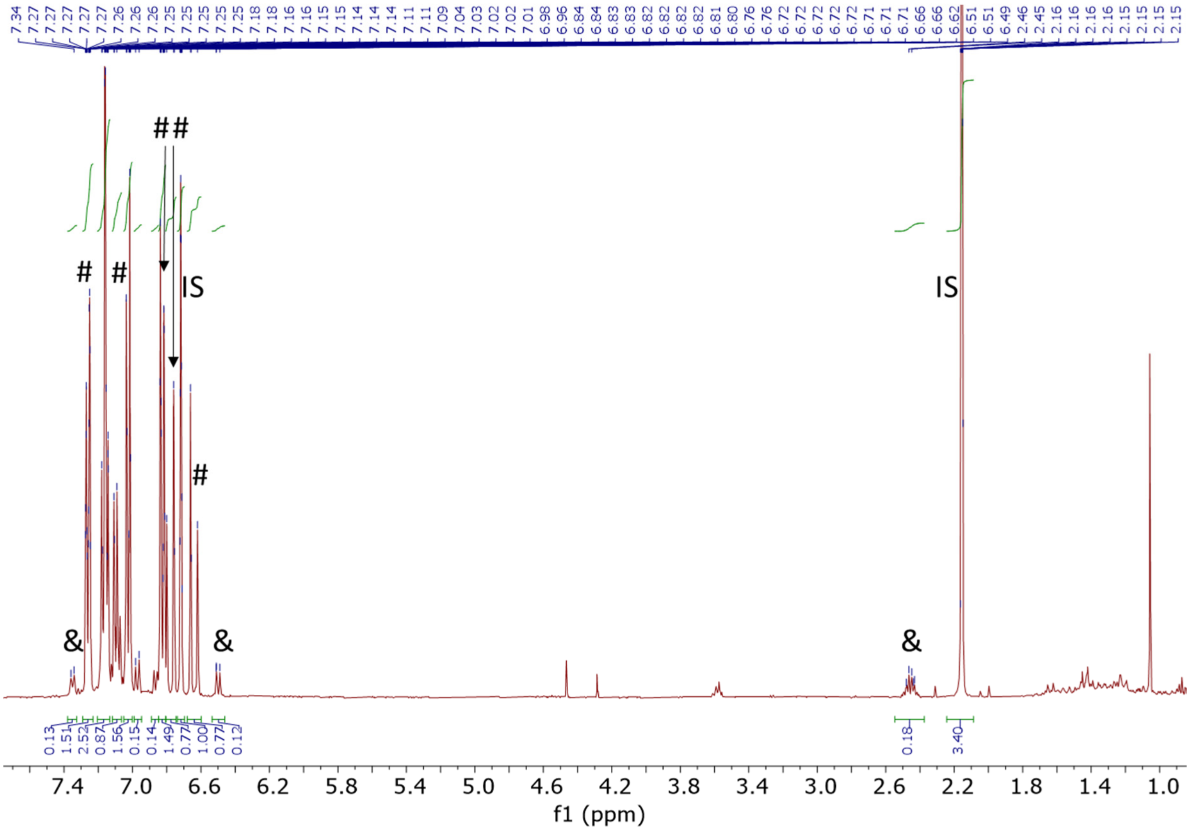

Figure S75: The ^{1}H NMR spectrum measured at 25 °C in C_6D_6 of the semi-hydrogenation of 4-(2-phenylethynyl)benzonitrile (%) with mesitylene as internal standard (IS) after 24 h at 40 °C. The corresponding *Z*-alkene is assigned by ^, the corresponding *E*-alkene is assigned by # and the corresponding alkane is assigned by &.

4-(phenylethynyl)benzaldehyde

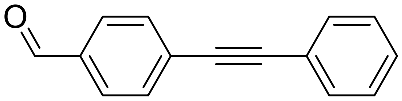

The ^{1}H NMR spectra of the semi-hydrogenation of 4-(phenylethynyl)benzaldehyde before and after H_2 addition are shown in Figures S76-S79. The substrate is not fully consumed after 24 or 48 h at 25 °C. After 24 h, the starting material, the *Z*-alkene, *E*-alkane, alkane and alcohol product(s) could be identified. Performing the semi-hydrogenation at 40 °C for 24 h results in full conversion of the starting material and the *E*-alkene and alkane product could be identified. However, the mass balance was poor for this reaction due to precipitation of presumably *E*-alkene alcohol which is formed in the reaction. The *Z*- and *E*- alkene products could be distinguished by their *J*-coupling constants (^{3}J = 12.2 Hz for *Z*-alkene and ^{3}J = 16.3 Hz for *E*-alkene). The main product formed after performing the semi-hydrogenation at 40 °C is proposed to be the *E*-alkene alcohol (aldehyde hydrogenated), based on the singlet at δ = 7.02 ppm, assigned to its alkene protons. An ^{1}H NMR spectrum of this reaction mixture in $CDCl_3$ containing the proposed *E*-alkene alcohol closely resembles that previously reported.[22] Additionally, the spectrum in $CDCl_3$ matches that for the *E*-alkene (aldehyde)[18] and for the alkane as previously reported.[21]

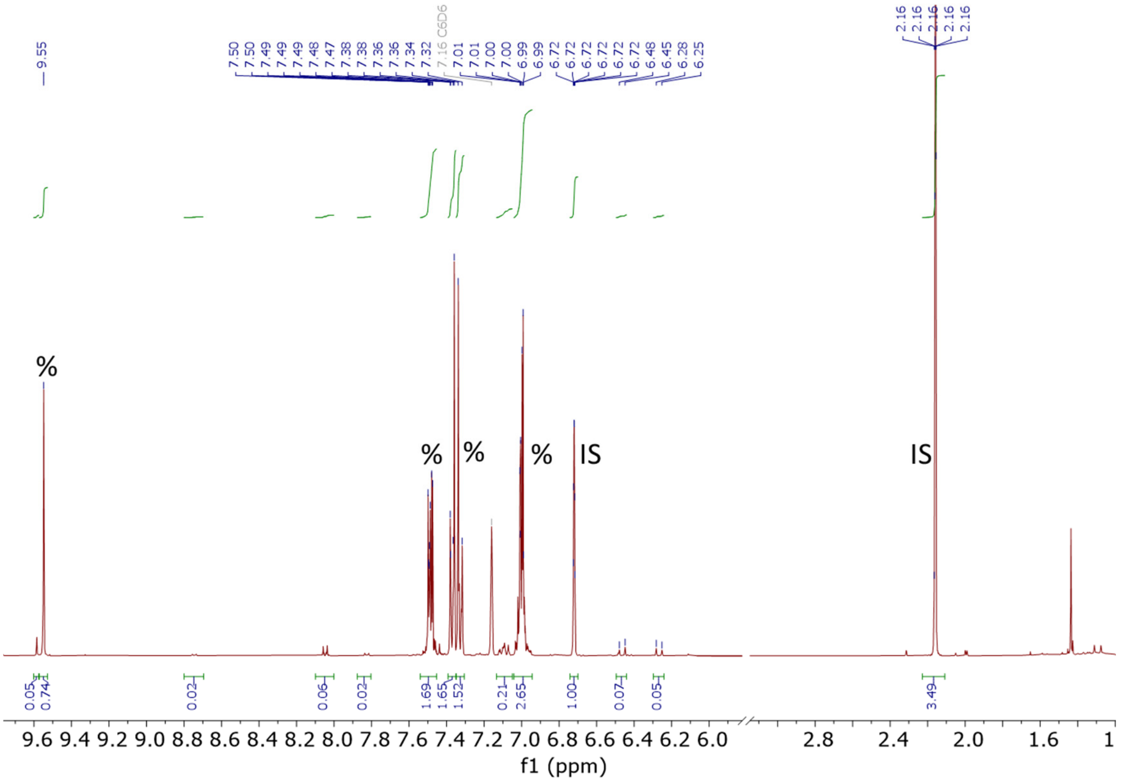

Figure S76: The ^{1}H NMR spectrum measured at 25 °C in C_6D_6 of the semi-hydrogenation of 4-(phenylethynyl)benzaldehyde (%) with mesitylene as internal standard (IS) before the addition of a H_2 atmosphere.

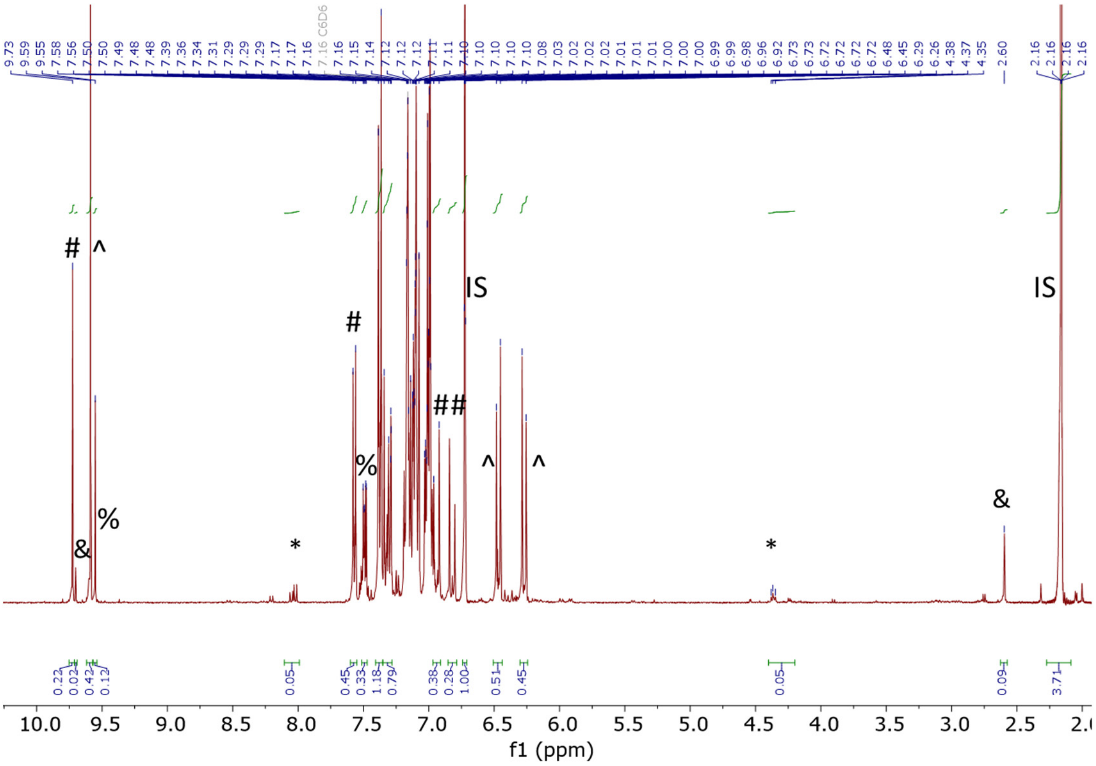

Figure S77: The ^{1}H NMR spectrum measured at 25 °C in C_6D_6 of the semi-hydrogenation of 4-(phenylethynyl)benzaldehyde (%) with mesitylene as internal standard (IS) after 24 h at 25 °C. The corresponding *Z*-alkene is assigned by ^, the corresponding *E*-alkene is assigned by # and the corresponding alkane is assigned by &. The resonances indicated by a * are assigned to an alcohol product.

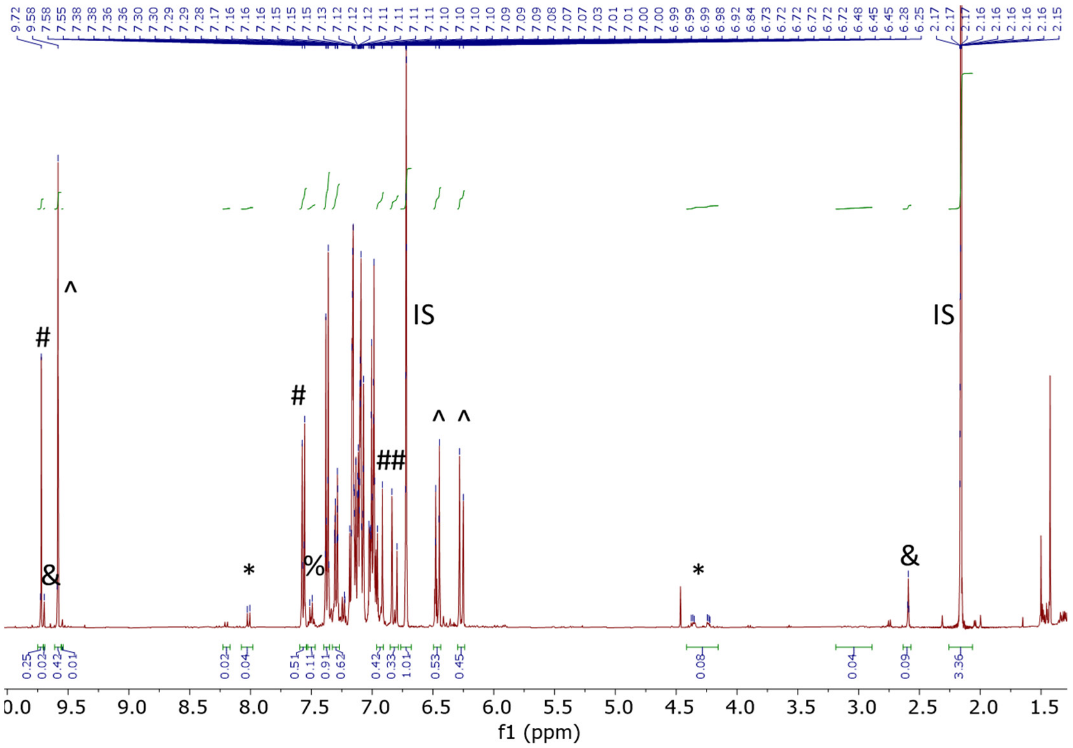

Figure S78: The ^{1}H NMR spectrum measured at 25 °C in C_6D_6 of the semi-hydrogenation of 4-(phenylethynyl)benzaldehyde (%) with mesitylene as internal standard (IS) after 48 h at 25 °C. The corresponding *Z*-alkene is assigned by ^, the corresponding *E*-alkene is assigned by # and the corresponding alkane is assigned by &. The resonances indicated by a * are assigned to (an) alcohol product(s).

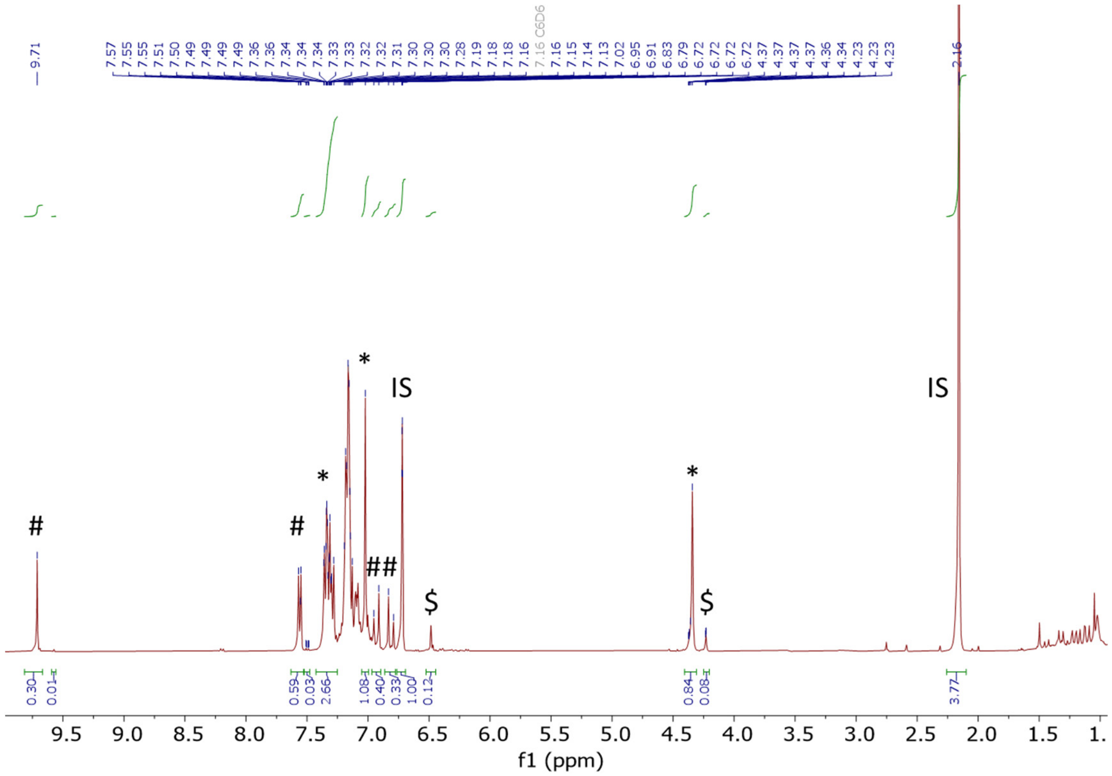

Figure S79: The ^{1}H NMR spectrum measured at 25 °C in C_6D_6 of the semi-hydrogenation of 4-(phenylethynyl)benzaldehyde (%) with mesitylene as internal standard (IS) after 24 h at 25 °C. The corresponding *Z*-alkene is assigned by ^, the corresponding *E*-alkene is assigned by # and the corresponding alkane is assigned by &. The resonances indicated by a * are assigned to (an) alcohol product(s), which resembles the *E*-alkene alcohol product due to the singlet at 7.02 ppm assigned to its alkene protons.

N-phenyl-1-(4-(phenylethynyl)phenyl)methanimine

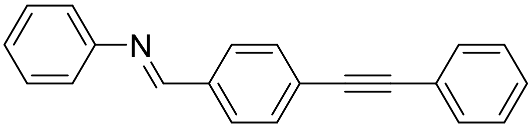

The ^{1}H NMR spectra of the semi-hydrogenation of *N*-phenyl-1-(4-(phenylethynyl)phenyl)methanimine before and after H_2 addition are shown in Figures S80-S81. The substrate is fully consumed after 24 h at 25 °C. After 24 h, *E*-alkane, alkane and amine product(s) could be identified. A poor mass balance is observed for this reaction due to the precipitation of (presumably) amine containing products. Due to a lack of previous reports on the expected products of this reaction, the assignment is based on chemical shifts, *J*-coupling constants and integral ratios. The *E*-alkene (imine) product has alkene resonances with *J*-coupling constants of ^3J = 16.3 Hz. The alkane product is assigned based on the resonance at δ = 2.69 ppm and other minor resonances that have matching integrals. The presence of an amine product is proposed based on the resonances observed at δ = 3.42 and 3.95 ppm that couple with each other and correspond to one and two protons, respectively, as expected for an amine product.

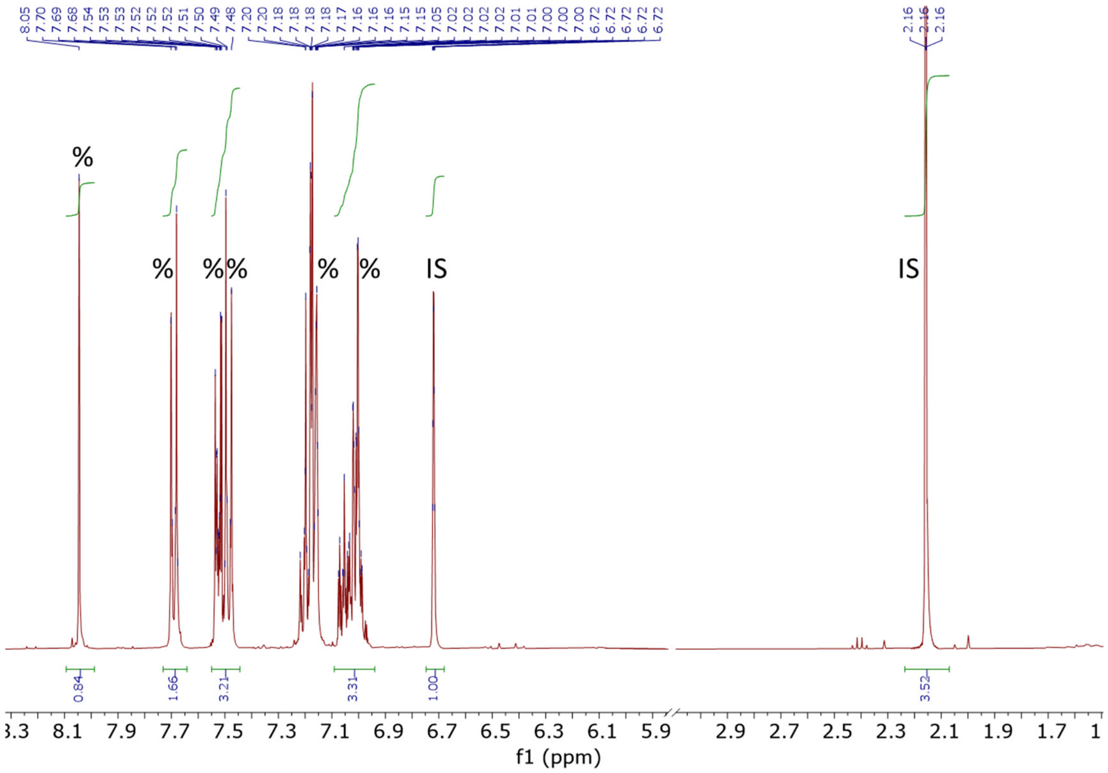

Figure S80: The ^{1}H NMR spectrum measured at 25 °C in C_6D_6 of the semi-hydrogenation of *N*-phenyl-1-(4-(phenylethynyl)phenyl)methanimine (%) with mesitylene as internal standard (IS) before the addition of a H_2 atmosphere.

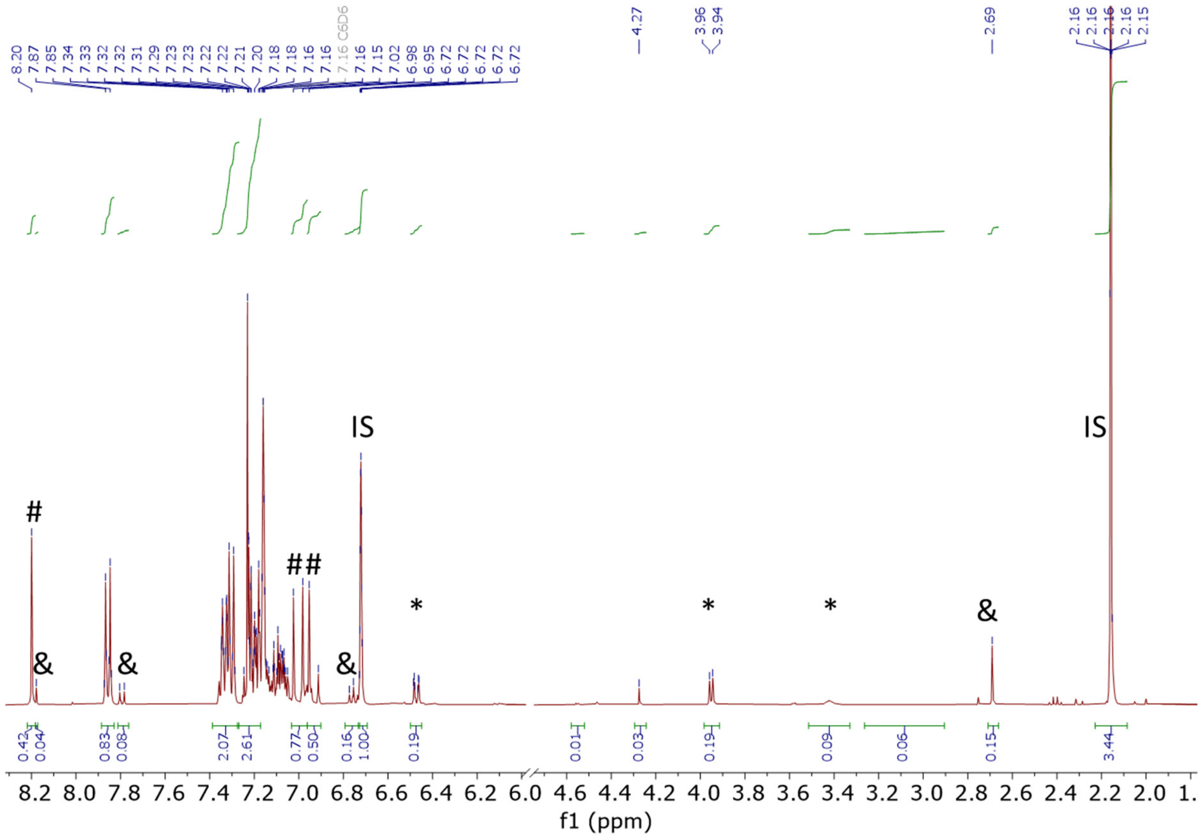

Figure S81: The ^{1}H NMR spectrum measured at 25 °C in C_6D_6 of the semi-hydrogenation of *N*-phenyl-1-(4-(phenylethynyl)phenyl)methanimine (%) with mesitylene as internal standard (IS) after 24 h at 25 °C. The corresponding *E*-alkene is assigned by # and the corresponding alkane is assigned by &. The resonances indicated by a * are assigned to unidentified amine products.

4-(phenylethynyl)anisole

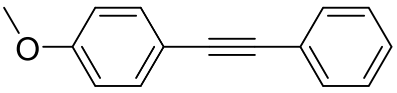

The ^{1}H NMR spectra of the semi-hydrogenation of methyl 4-(phenylethynyl)anisole before and after H_2 addition are shown in Figures S82-S84. Full conversion of the starting material is observed after 48 h. Its corresponding *E*-alkene and alkane products are identified. After 24 h, some starting material is still present as well as a *Z*-alkene product (^{3}J = 12.3 Hz). The products of this reaction have not been reported in C_6D_6. However, the *J*-coupling constants observed for the alkene protons are consistent of that of an *E*-alkene (^{3}J = 16.4 Hz). The ^{1}H NMR spectrum of the reaction mixture in $CDCl_3$ matches that of the *E*-alkene[18] and the alkane product.[23]

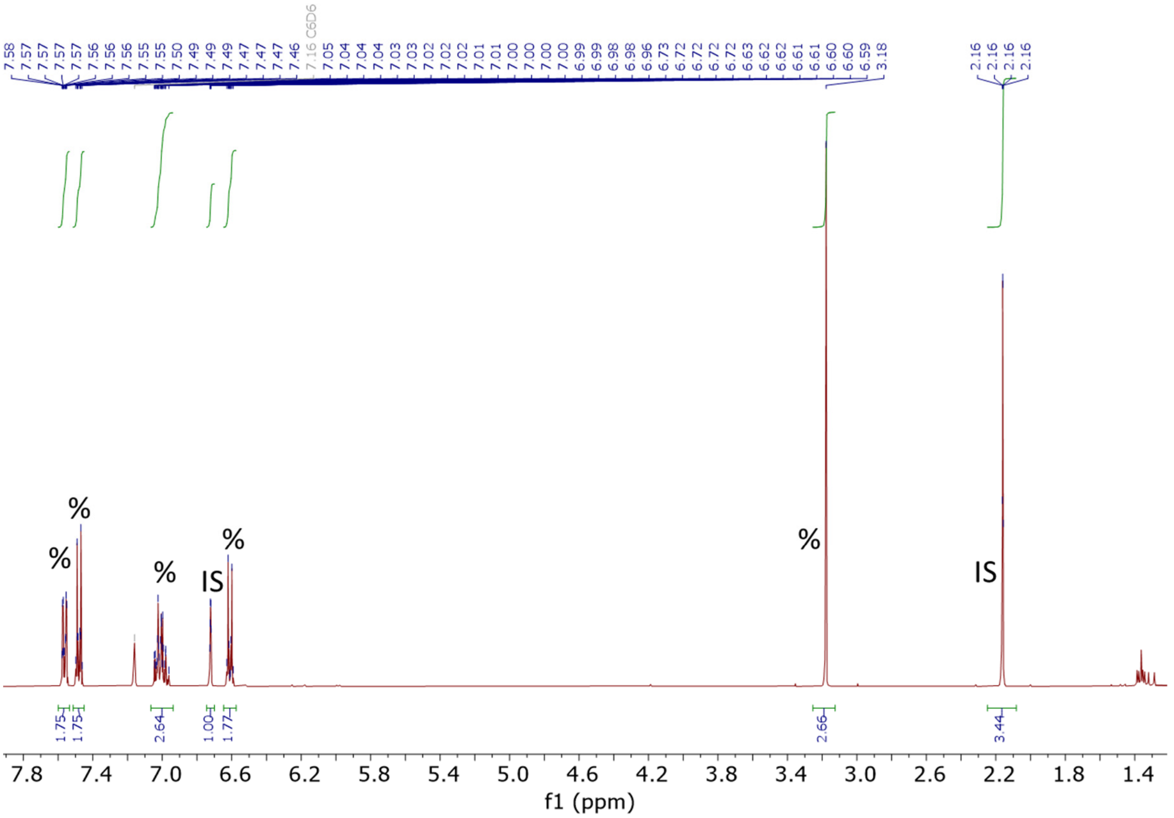

Figure S82: The ^{1}H NMR spectrum measured at 25 °C in C_6D_6 of the semi-hydrogenation of 4-(phenylethynyl)anisole (%) with mesitylene as internal standard (IS) before the addition of a H_2 atmosphere.

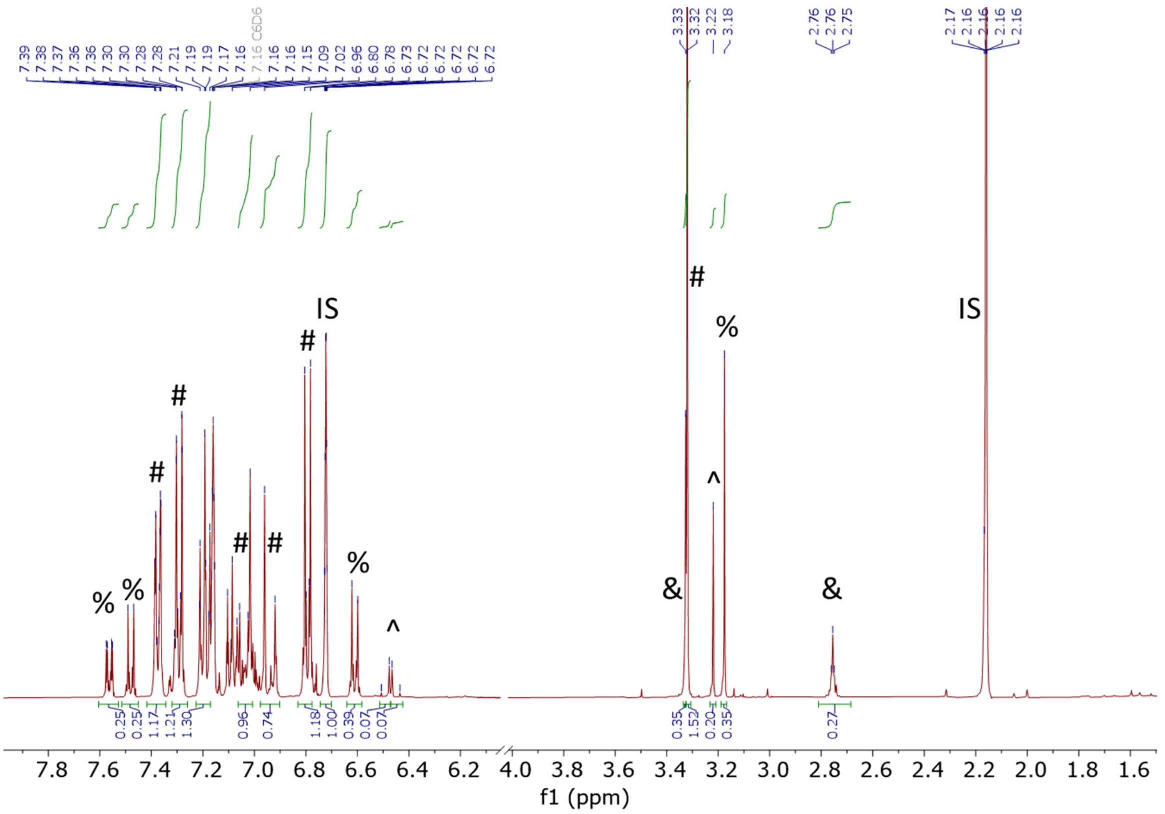

Figure S83: The ^{1}H NMR spectrum measured at 25 °C in C_6D_6 of the semi-hydrogenation of 4-(phenylethynyl)anisole (%) with mesitylene as internal standard (IS) after 24 h at 25 °C. The corresponding *Z*-alkene is assigned by ^, the corresponding *E*-alkene is assigned by # and the corresponding alkane is assigned by &.

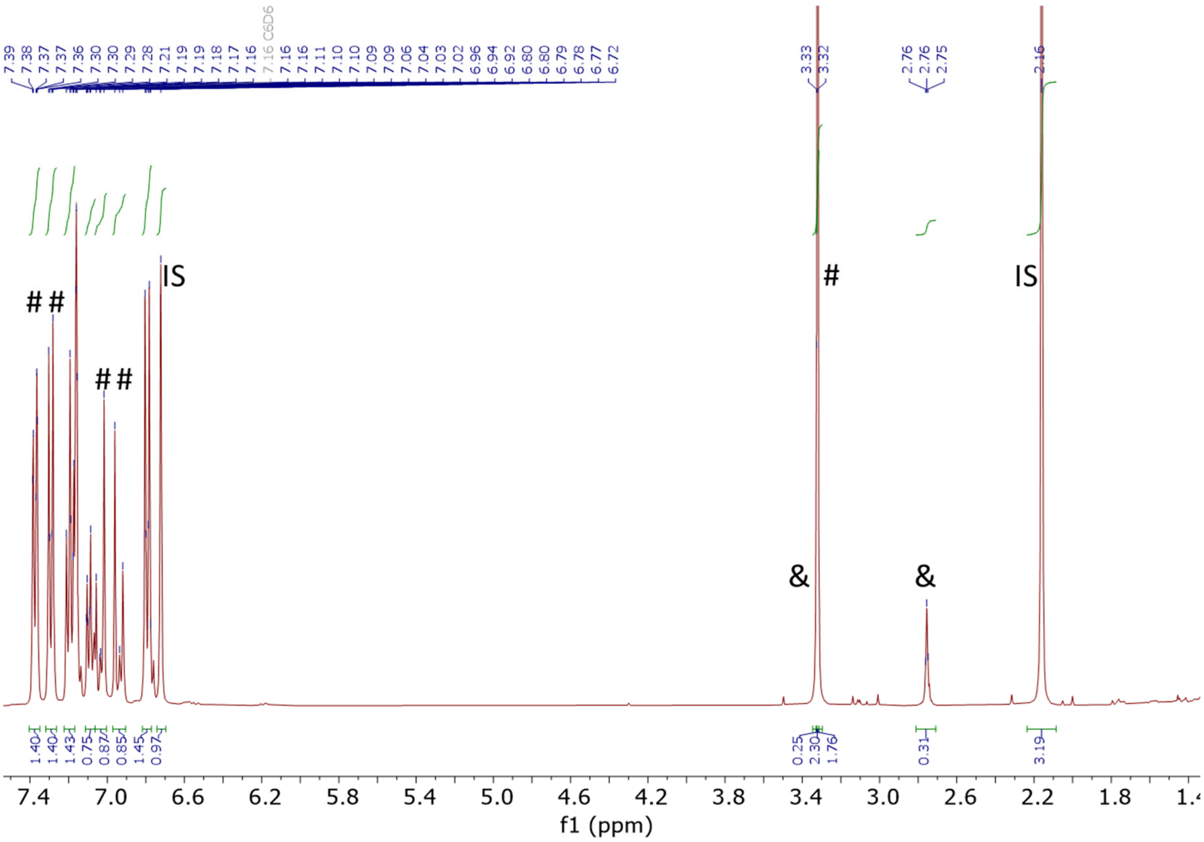

Figure S84: The ^{1}H NMR spectrum measured at 25 °C in C_6D_6 of the semi-hydrogenation of 4-(phenylethynyl)anisole (%) with mesitylene as internal standard (IS) after 48 h at 25 °C. The corresponding *Z*-alkene is assigned by ^, the corresponding *E*-alkene is assigned by # and the corresponding alkane is assigned by &.

Semi-hydrogenation of alkyl substituted alkynes

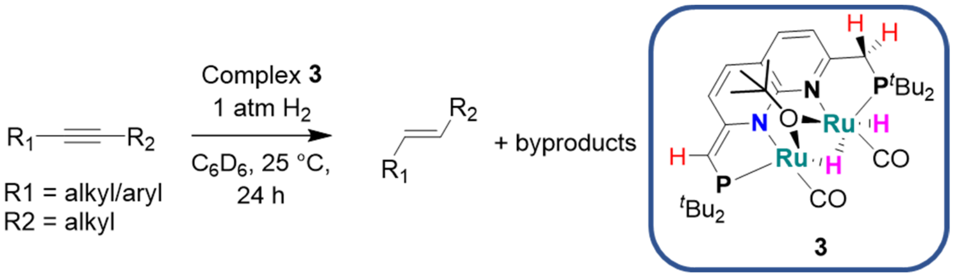

E-selective semi-hydrogenation of alkyl-substituted alkynes are often more challenging than diaryl-substituted substrates due to the tendency for over-reduction to alkanes or giving mixtures of both *E*- and *Z*-alkenes. Therefore, we explored how an aryl-alkyl and an alkyl-alkyl substrate perform under identical catalytic conditions compared to diphenylacetylene (aryl-aryl) substrates.

1-phenyl-1-propyne

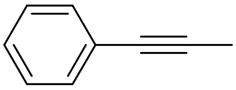

The ^{1}H NMR spectra of the semi-hydrogenation of methyl 4-(phenylethynyl)anisole before and after H_2 addition are shown in Figures S85 and S86. Full conversion of the starting material is observed after 24 h. The reference spectra of all expected products have been reported before in C_6D_6. The major product is the *E*-alkene[24] (61%) and traces of the *Z*-alkene[24] (2%). Interestingly, significant amount of the fully hydrogenated propylbenzene (38%) is observed, indicating that there is a higher activity for this aryl-alkyl substrate compared to aryl-aryl substrates.

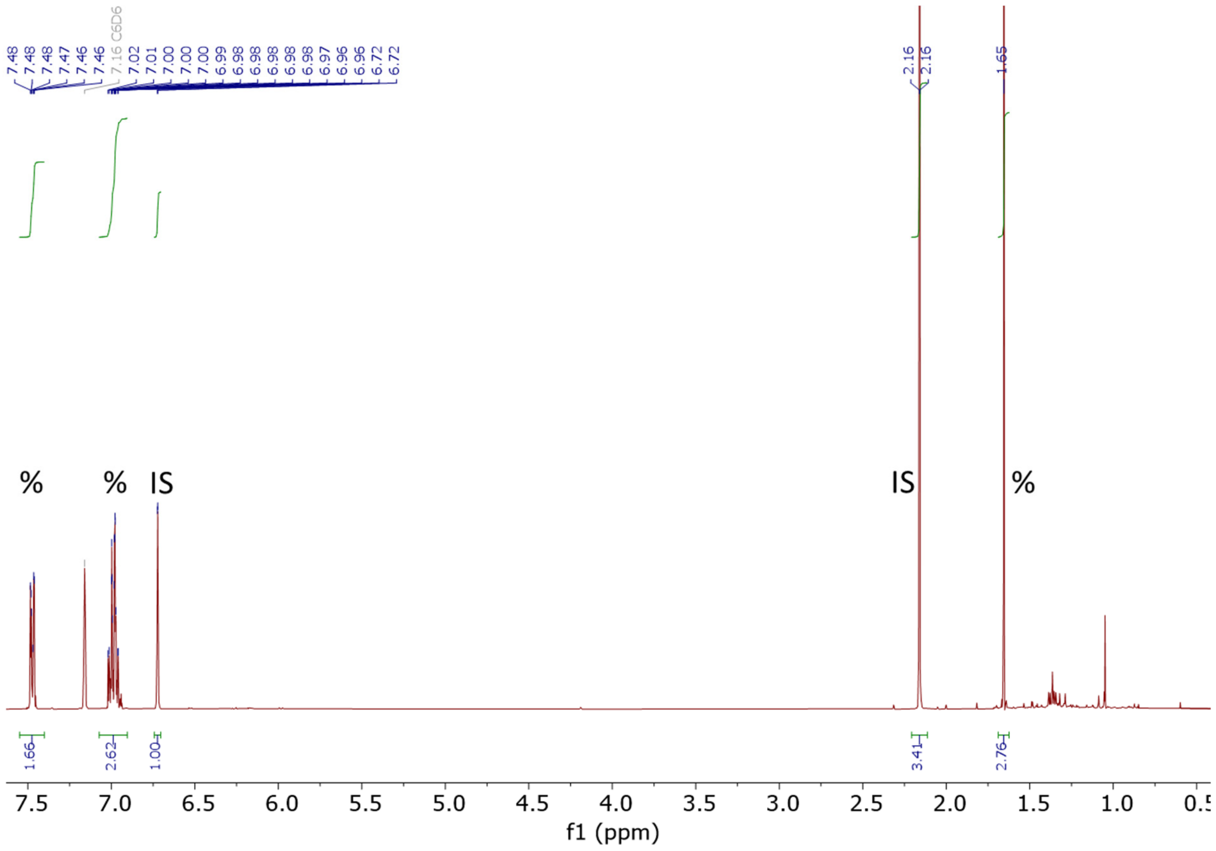

Figure S85: The ^{1}H NMR spectrum measured at 25 °C in C_6D_6 of the semi-hydrogenation of 1-phenyl-1-propyne (%) with mesitylene as internal standard (IS) before the addition of a H_2 atmosphere.

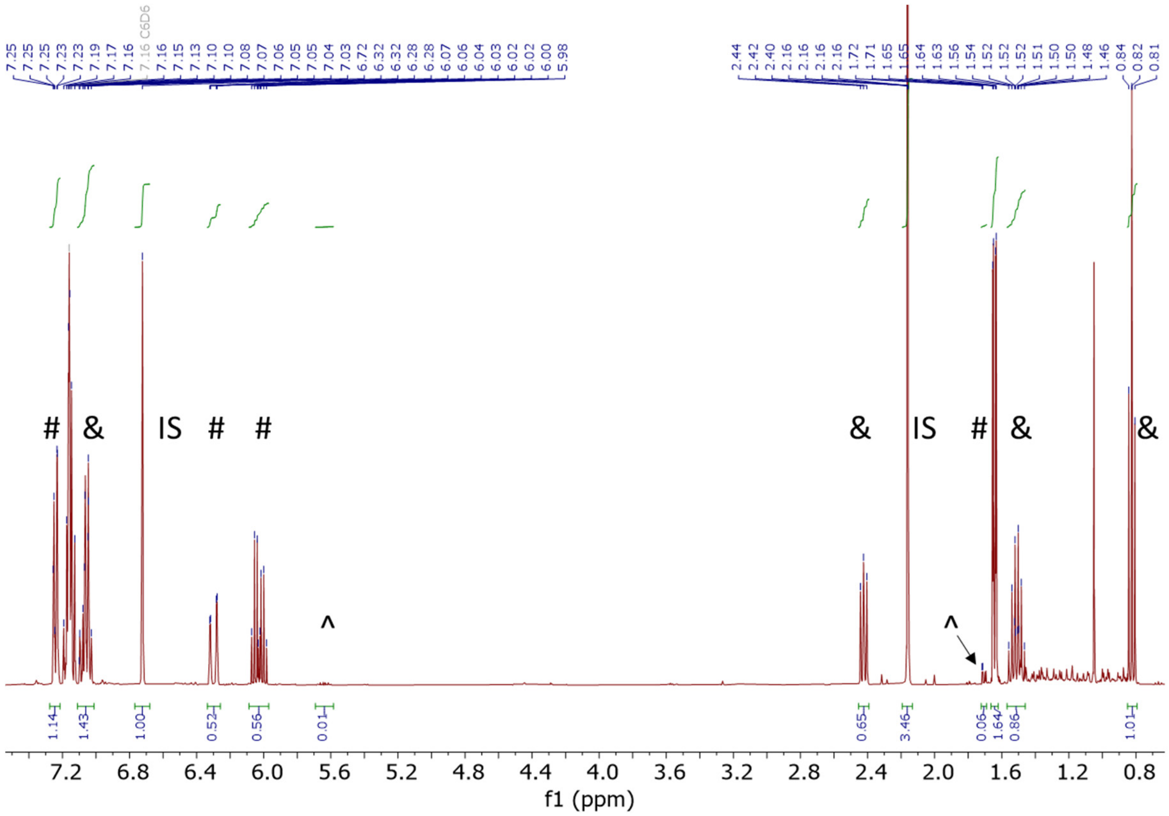

Figure S86: The ^{1}H NMR spectrum measured at 25 °C in C_6D_6 of the semi-hydrogenation of 1-phenyl-1-propyne (%) with mesitylene as internal standard (IS) after 24 h at 25 °C. The corresponding *Z*-alkene is assigned by ^, the corresponding *E*-alkene is assigned by # and the corresponding alkane is assigned by &.

3-propyne

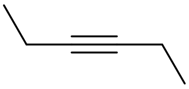

The ^{1}H and ^{13}C{^{1}H} NMR spectra of the semi-hydrogenation of methyl 4-(phenylethynyl)anisole before and after H_2 addition are shown in Figures S87-S89. Full conversion of the starting material is observed after 24 h with the fully hydrogenated product hexane as major product (73%). Interestingly, a mixture of three different hexenes are observed in the ^{1}H and ^{13}C NMR spectra of the reaction mixture after 24 h. Both the expected product *E*-3-hexene (11%), as a mixture of *E*-2-hexene (11%) and *Z*-2-hexene (4%) were identified and their resonances in the NMR spectra match with previously reported data.[25] This result indicates that the double bond isomerizes from 3-hexene to the two different 2-hexene products.

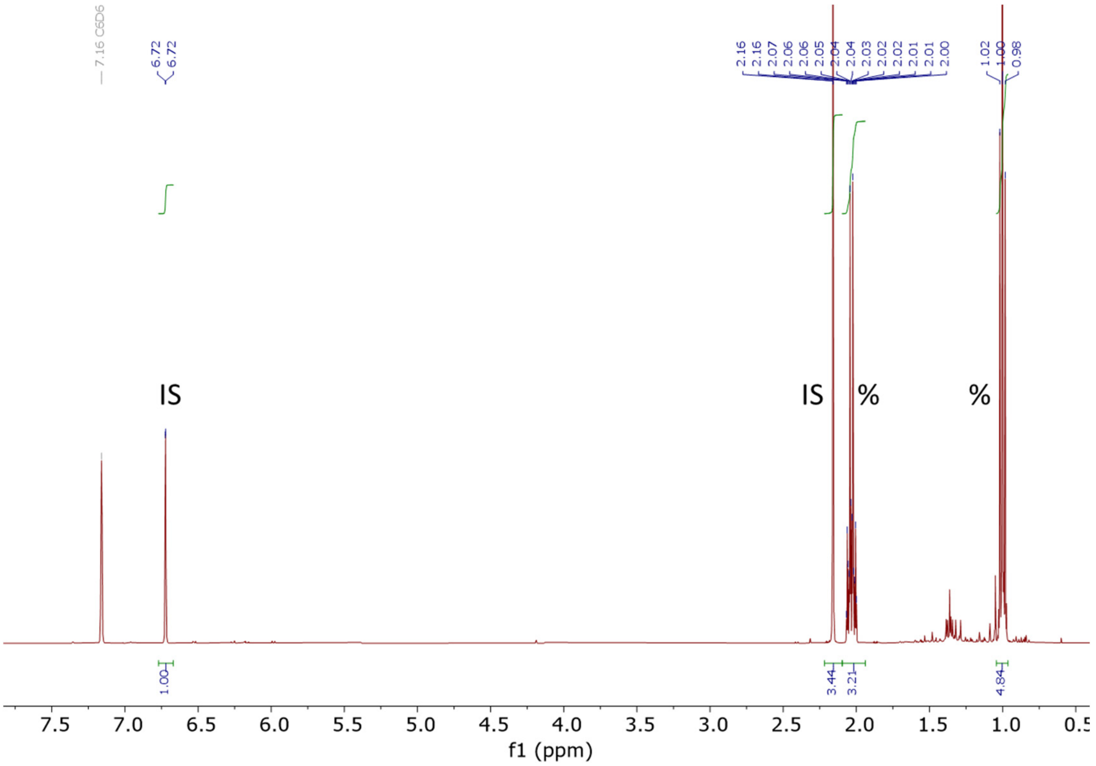

Figure S87: The ^{1}H NMR spectrum measured at 25 °C in C_6D_6 of the semi-hydrogenation of 3-propyne (%) with mesitylene as internal standard (IS) before the addition of a H_2 atmosphere.

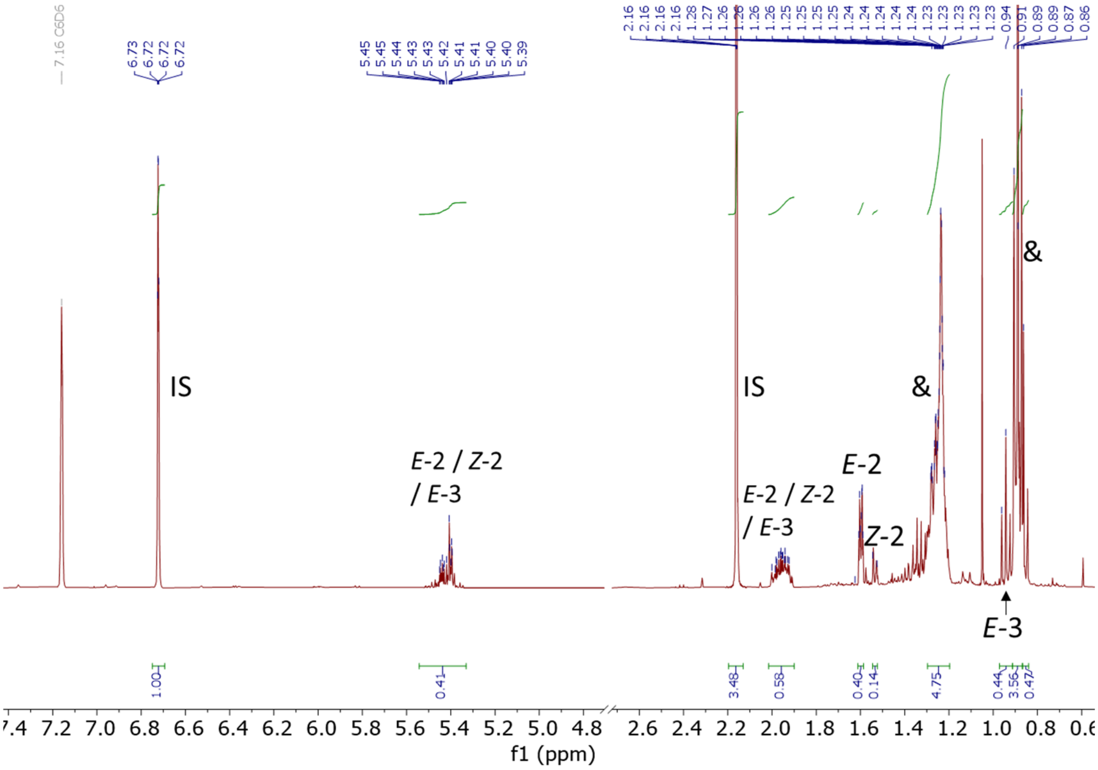

Figure S88: The ^{1}H NMR spectrum measured at 25 °C in C_6D_6 of the semi-hydrogenation of 3-propyne (%) with mesitylene as internal standard (IS) after 24 h at 25 °C. Full conversion of the starting material to the major product hexane, assigned by &, is observed. Additionally, a mixture of *Z*-2-hexene assigned by *Z*-2, *E*-2-hexene assigned by *E*-2, and *E*-3-hexene assigned by *E*-3, is observed as minor species.

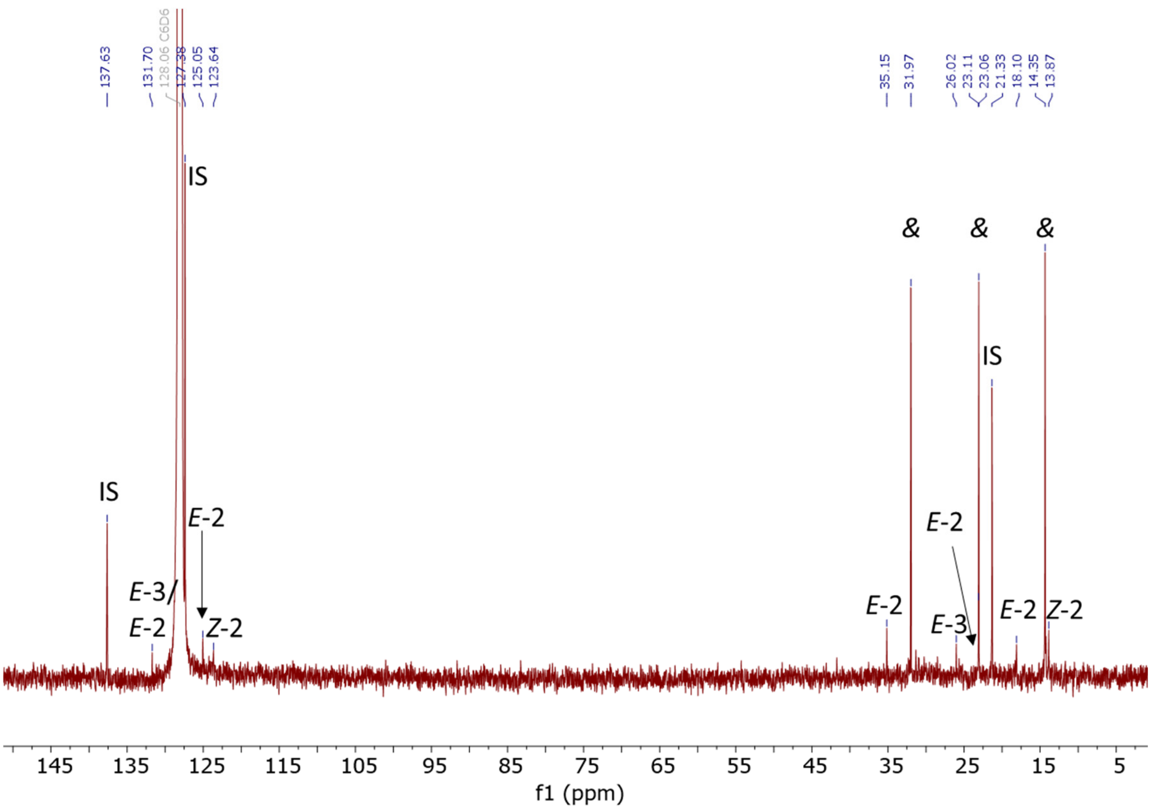

Figure S89: The $^{13}C\{^1H\}$ NMR spectrum measured at 25 °C in C_6D_6 of the semi-hydrogenation of 3-propyne with mesitylene as internal standard (IS) after 24 h at 25 °C. Full conversion of the starting material to the major product hexane, assigned by &, is observed. Additionally, a mixture of *Z*-2-hexene assigned by *Z*-2, *E*-2-hexene assigned by *E*-2, and *E*-3-hexene assigned by *E*-3, is observed as minor species.

Computational methods

All calculations were performed using DFT with the ORCA program package, version 4.2.1.[26] All calculations were performed using the BP86[27] or the B3LYP[28] functional in combination with the scalar relativistically recontracted version of the Ahlrichs triple-ζ basis set def2-TZVP[29] on all atoms in the molecule. Additionally, the atom-pairwise dispersion correction with the Becke-Johnson damping scheme (D3BJ)[30] was used for all atoms in every calculation. For all structures, the geometry was optimized using the BP86 functional. The atom coordinates used for geometry optimizations for complexes **2** and **5** were taken from the single crystal X-ray structures and for complexes **1** and **3** based on the X-ray diffraction structure of **2**. The keyword 'TightSCF' was included for tightly converging SCF calculations. Geometry optimizations were performed with the keyword 'TightOpt' to tightly converge the optimization. The numerical frequency of the optimized geometries were calculated (except for proposed isomers 1 and 2 of complex **1**) to establish if the geometry converged to a minimum or a saddle point.

Example of an input file for the geometry optimization of complex **2** (excluding PF_6^-)**:**

```
! RKS BP86 Opt NumFreq def2-TZVP def2/J Grid4 NoFinalGrid
! TightSCF D3BJ TightOpt

%pal nprocs 24 end

%scf MaxIter 500 end


* xyz 1 1
[Cartesian coordinates here]
*
```

Comparison of selected parameters for complexes 1, 2, 3 and 5

Table S4: Comparison of structural and physical parameters of complexes **1**, **2**, **3** and **5**.

Complex	Ru–Ru (Å)	Bench stable?	ν (Ru–H) (cm^{-1})	ν (CO) (cm^{-1})	δ Hydride ligands (ppm)	Hydride coordination
**1**	EXAFS: 2.81	yes	2021	1942, 1905	-14.3, -16.8 (ssNMR)	1 terminal 1 bridging
**2**	2.8149(10)	yes	2069	1972, 1938	-14.52 (d), -15.80 (dd)	1 terminal 1 bridging
**3**	N/A	no	N/A	1923, shoulder at ~1890	-15.76 (d), -20.97 (dd)	1 terminal 1 bridging
**5**	Ru1–Ru2 2.7364(3) Ru1–Ru1^{i} 2.8627(4)	no	~2100 (br)	1950, 1925, 1897	THF: -13.39 (dd), -20.48- -20.68 (m, 2H) DCM: -13.40 (dd), -20.18 – -20.31 (m), -20.45 (dd)	1 terminal 2 bridging

References

[1] Gnanaprakasam, B.; Zhang, J.; Milstein, D. Direct Synthesis of Imines from Alcohols and Amines with Liberation of H2. *Angew. Chem. Int. Ed.* **2010**, *49* (8), 1468–1471.
[2] Sletten, E. M.; Bertozzi, C. R. A Bioorthogonal Quadricyclane Ligation. *J. Am. Chem. Soc.* **2011**, *133*, 44, 17570–17573.
[3] Fuji, K.; Morimoto, T.; Tsutsumi, K.; Kakiuchi, K. Rh(i)-catalyzed CO gas-free cyclohydrocarbonylation of alkynes with formaldehyde to α,β-butenolides. *Chem. Commun*. **2005**, 3295-3297
[4] Ahmed, M. A.; Akitoshi, S.; Kentaro, M.; Atsunori, M. Aqueous Ammonia as a New Activator for Sonogashira Coupling. *Bull. Chem. Soc. Jpn* **2005**, *78*, 160-168.
[5] Senkala, S.; Malecki, J. G.; Vasylieva, M.; Labuz, A.; Nosek, K.; Piwowarczyk, K.; Czyz, J.; Schab-Balcerzak, W.; Janeczek, H.; Korzec, M. Hydrolysis of Schiff bases with phenyl-ethynyl-phenyl system: The importance for biological and physicochemical studies. *J. Photochem. Photobiol. B: Biol.* **2020**, *212*, 112020.
[6] Ravel, B.; Newville, M. Athena, artemis, Hephaestus: data analysis for X-ray absorption spectroscopy using IFEFFIT. *J. Synchrotron Radiat.* **2005**, *12*, 537-541.
[7] Tenderholt, A.; Hedman, B.; Hodgson, K. O. PySpline: A Modern, Cross-Platform Program for the Processing of Raw Averaged XAS Edge and EXAFS Data. *AIP Conference Proceedings*. **2007**, *882*, 105-107.
[8] Bunker, G. Introduction to XAFS: A Practical Guide to X-ray Absorption Fine Structure Spectroscopy. Cambridge University Press (2010).
[9] Kupce, E.; Freeman, R. Frequency-domain Hadamard spectroscopy. *J. Mag. Res.* **2003**, *162*, 158-165.
[10] Schreurs, A. M. M.; Xian, X.; Kroon-Batenburg, L. M. J. "*EVAL15*: a diffraction data integration method based on *ab initio* predicted profiles". *J. Appl. Cryst.* **2010**, *43*, 70-82.
[11] Sheldrick, G. M. SADABS and TWINABS, **2014**. Universität Göttingen, Germany.
[12] Sheldrick, G. M. "*SHELXT* - Integrated space-group and crystal-structure determination". *Acta Cryst.* **2015**, *A71*, 3-8.
[13] Sheldrick, G. M. "Crystal structure refinement with *SHELXL*". *Acta Cryst.* **2015**, *C71*, 3-8.
[14] Spek, A. L. "Structure validation in chemical crystallography". *Acta Cryst.* **2009**, *D65*, 148-155.
[15] Parsons, S.; Flack, H. D.; Wagner, T. "Use of intensity quotients and differences in absolute structure refinement". *Acta Cryst.* **2013**, *B69*, 249-259.
[16] The solvent dependence on the *J*-coupling constants in alkenes is marginal, see: Barfield, M.; Johnston, M. D. Solvent dependence of nuclear spin-spin coupling constants. *Chem. Rev.* **1973**, *73*, 1, 53–73.
[17] Martin, J.; Knüpfer, C.; Eyselein, J.; Färber, C.; Grams, S.; Langer, J.; Thum, K.; Wiesinger, M.; Harder, S. Highly Active Superbulky Alkaline Earth Metal Amide Catalysts for Hydrogenation of Challenging Alkenes and Aromatic Rings. *Angew. Chem. Int. Ed.* **2020**, *59*, 9102-9112.
[18] Fu, S.; Chen, N.-Y.; Liu, X.; Shao, Z.; Luo, S.-P.; Liu, Q. Ligand-Controlled Cobalt-Catalyzed Transfer Hydrogenation of Alkynes: Stereodivergent Synthesis of Z- and E-Alkenes. *J. Am. Chem. Soc.* **2016**, *138*, 27, 8588–8594.
[19] Rushworth, P. J.; Hulcoop, D. G.; Fox, D. J. Iron/Tetramethylethylenediamine-Catalyzed Ambient-Temperature Coupling of Alkyl Grignard Reagents and Aryl Chlorides. *J. Org. Chem.* **2013**, *78*, 18, 9517–9521.
[20] Brzozowska, A.; Azofra, L. M.; Zubar, V.; Atodiresei, I.; Cavallo, L.; Rueping, M.; El-Sepelgy, O. Highly Chemo- and Stereoselective Transfer Semihydrogenation of Alkynes Catalyzed by a Stable, Well-Defined Manganese(II) Complex. *ACS Catal.* **2018**, *8*, 5, 4103–4109.
[21] Zhi, M.-L.; Chen, B.-Z.; Deng, W.; Chu, X.-Q.; Loh, T.-P.; Shen, Z.-L. Preparation of Alkyl Indium Reagents by Iodine-Catalyzed Direct Indium Insertion and Their Applications in Cross-Coupling Reactions. *J. Org. Chem.* **2019**, *84*, 5, 3017–3023.
[22] Takemoto, S.; Kitamura, M.; Saruwatari, S.; Isono, A.; Takda, Y.; Nishimori, R.; Tsujiwaki, M.; Sakaue, N.; Matsuzaka, H. Bis(bipyridine) ruthenium(ii) bis(phosphido) metalloligand: synthesis of heterometallic complexes and application to catalytic (*E*)-selective alkyne semi-hydrogenation. *Dalton Trans.,* **2019**, *48*, 1161-1165.
[23] Jiao, K.-J.; Ma, H.-X.; Qiu, H.; Fang, P.; Mei, T.-S. Nickel-Catalyzed Electrochemical Reductive Relay Cross-Coupling of Alkyl Halides to Aryl Halides. *Angew. Chem. Int. Ed*. **2020**, *59*, 6520 –6524.
[24] Suslick, B. A.; Tilley, T. D. Mechanistic Interrogation of Alkyne Hydroarylations Catalyzed by Highly Reduced, Single-Component Cobalt Complexes. *J. Am. Chem. Soc.* **2020**, *142*, 25, 11203–11218.

[25] Schwieger, S.; Herzog, R.; Wagner, C.; Steinborn, D. Platina-β-diketones as Catalysts for Hydrosilylation and Their Reactivity towards Hydrosilanes. *J. Organomet. Chem.* **2009**, *694*, 3548-3558.

[26] Neese, F. The ORCA program system. *Wiley Interdiscip. Rev. Comput. Mol. Sci.* **2012**, *2*, 73–78.

[27] (a) A. D. Becke, A.D. Density functional calculations of molecular bond energies. *J. Chem. Phys.* **1986**, *84*, 4524-4529. (b) Perdew, J. P. Density-functional approximation for the correlation energy of the inhomogeneous electron gas. *Phys. Rev. B* **1986**, *33*, 8822–8824.

[28] Becke, A. D. Density-functional thermochemistry. III. The role of exact exchange. *J. Chem Phys.* **1993**, *98*, 5648-5652.

[29] (a) Pantazis, D. A.; Chen, X.-Y. ; Landis, C. R.; Neese, F. All-Electron Scalar Relativistic Basis Sets for Third-Row Transition Metal Atoms. *J. Chem. Theory Comput.* **2008**, *4*, 908–919. (b) Weigend, F.; Ahlrichs, R. Balanced basis sets of split valence, triple zeta valence and quadruple zeta valence quality for H to Rn: Design and assessment of accuracy. *Phys. Chem. Chem. Phys.* **2005**, *7*, 3297–3305.

[30] (a) Grimme, S.; Antony, J.; Ehrlich, S.; Krieg, H. A consistent and accurate ab initio parametrization of density functional dispersion correction (DFT-D) for the 94 elements H-Pu. *J. Chem. Phys.* **2010**, *132*, 154104. (b) Grimme, S.; Ehrlich, S.; Goerigk, L. Effect of the damping function in dispersion corrected density functional theory. *J. Comput. Chem.* **2011**, *32*, 1456–1465.
